# Supplementary material for: The CST complex mediates a post-resection non-homologous end joining repair pathway and promotes local deletions in Saccharomyces cerevisiae
Source: Cell Genom. 2025 Jul 16;5(10):100947. doi: 10.1016/j.xgen.2025.100947 (PMC12791002; doi:10.1016/j.xgen.2025.100947)
Supplement: Document S1. Figures S1–S8 and Tables S1–S3 [file mmc1.pdf]

Cell Genomics, Volume *5*

Supplemental information

The CST complex mediates a post-resection non-homologous end joining repair pathway and promotes local deletions in *Saccharomyces cerevisiae*

Oana Ilioaia, Liébaut Dudragne, Clémentine Brocas, Léa Meneu, Romain Koszul, Karine Dubrana, and Zhou Xu

SUPPLEMENTAL INFORMATION

Title: The CST complex mediates a post-resection non-homologous end-joining repair pathway and promotes local deletions in *Saccharomyces cerevisiae*

Authors: Oana Ilioaia[1], Liébaut Dudragne[1], Clémentine Brocas[2], Léa Meneu[3,4], Romain Koszul[3], Karine Dubrana[2] & Zhou Xu[1,*]

Affiliations:

[1]Sorbonne Université, CNRS, UMR7238, Institut de Biologie Paris-Seine, Laboratory of Computational, Quantitative and Synthetic Biology, CQSB, 75005 Paris, France.

[2]Université Paris Cité, Inserm, CEA, Stabilité Génétique Cellules Souches et Radiations, F-92260 Fontenay-aux-Roses, France.

[3]Institut Pasteur, CNRS UMR3525, Université Paris Cité, Unité Régulation Spatiale des Génomes, 75015 Paris, France.

[4]Sorbonne Université, Collège Doctoral.

*Lead contact

Correspondence:

zhou.xu@sorbonne-universite.fr

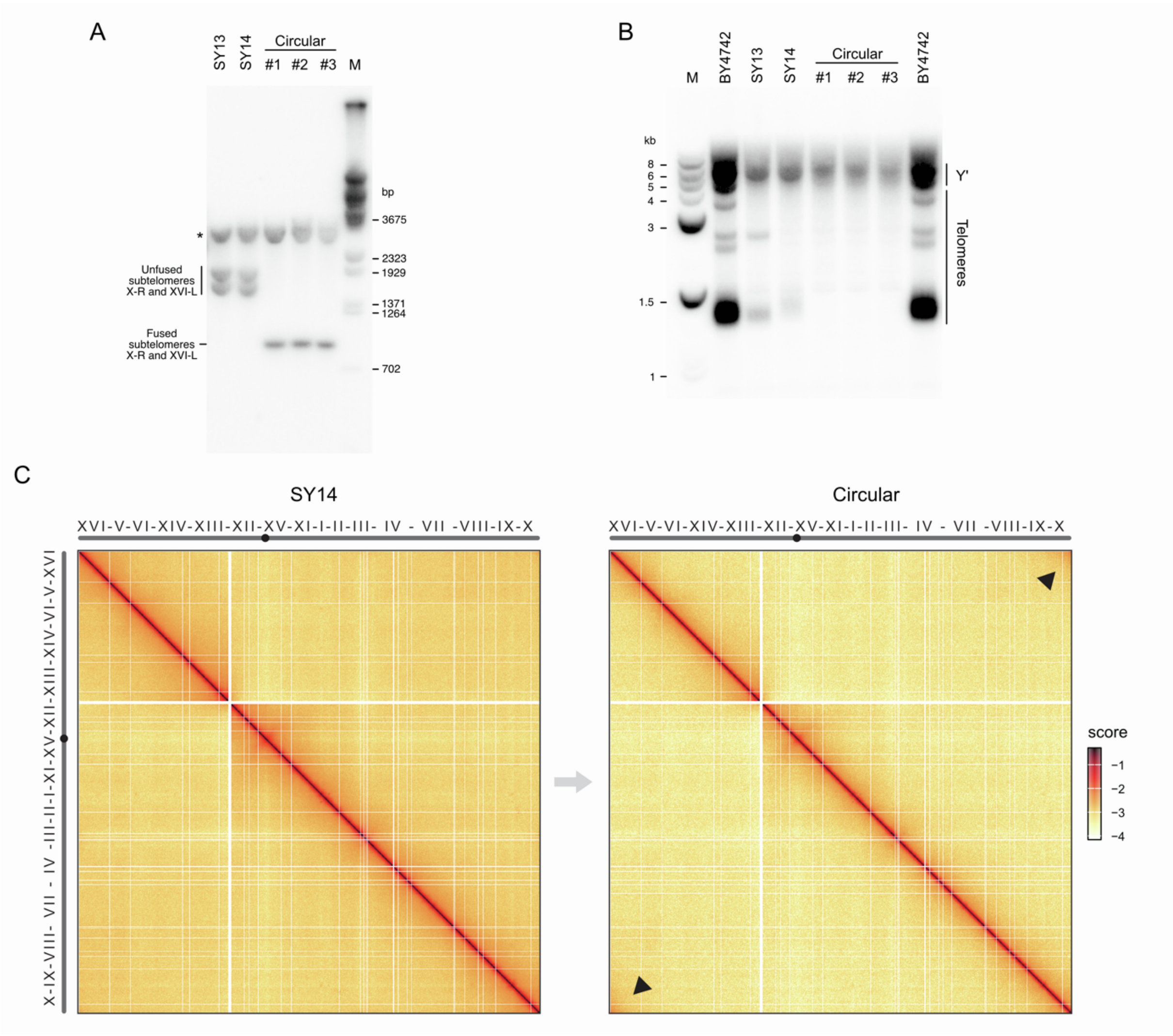

Supplemental Figure S1. Single chromosome circularization. Related to Figure 1.

(A) Southern blot for detecting the fusion between the two subtelomeres of SY14. HindIII- and NdeI-digested genomic DNA of the indicated strains was migrated, transferred and probed with a chimeric radiolabeled oligonucleotide complementary to both subtelomeres. Three independent cultures of the circular chromosome strain were tested. M: molecular weight marker (λ DNA, BstEII digest). *: non-specific band.

(B) Terminal restriction fragment Southern blot. XhoI-digested genomic DNA of the indicated strains was migrated, transferred and probed with a telomeric probe. M: molecular weight marker.

(C) Normalized Hi-C contact maps of the linear strain SY14 (left) and the single circular chromosome strain (right) with 16-kb resolution. Low to high interaction frequencies are depicted by a color

spectrum from light yellow to red. Arrows indicate contacts associated with chromosome circularization.

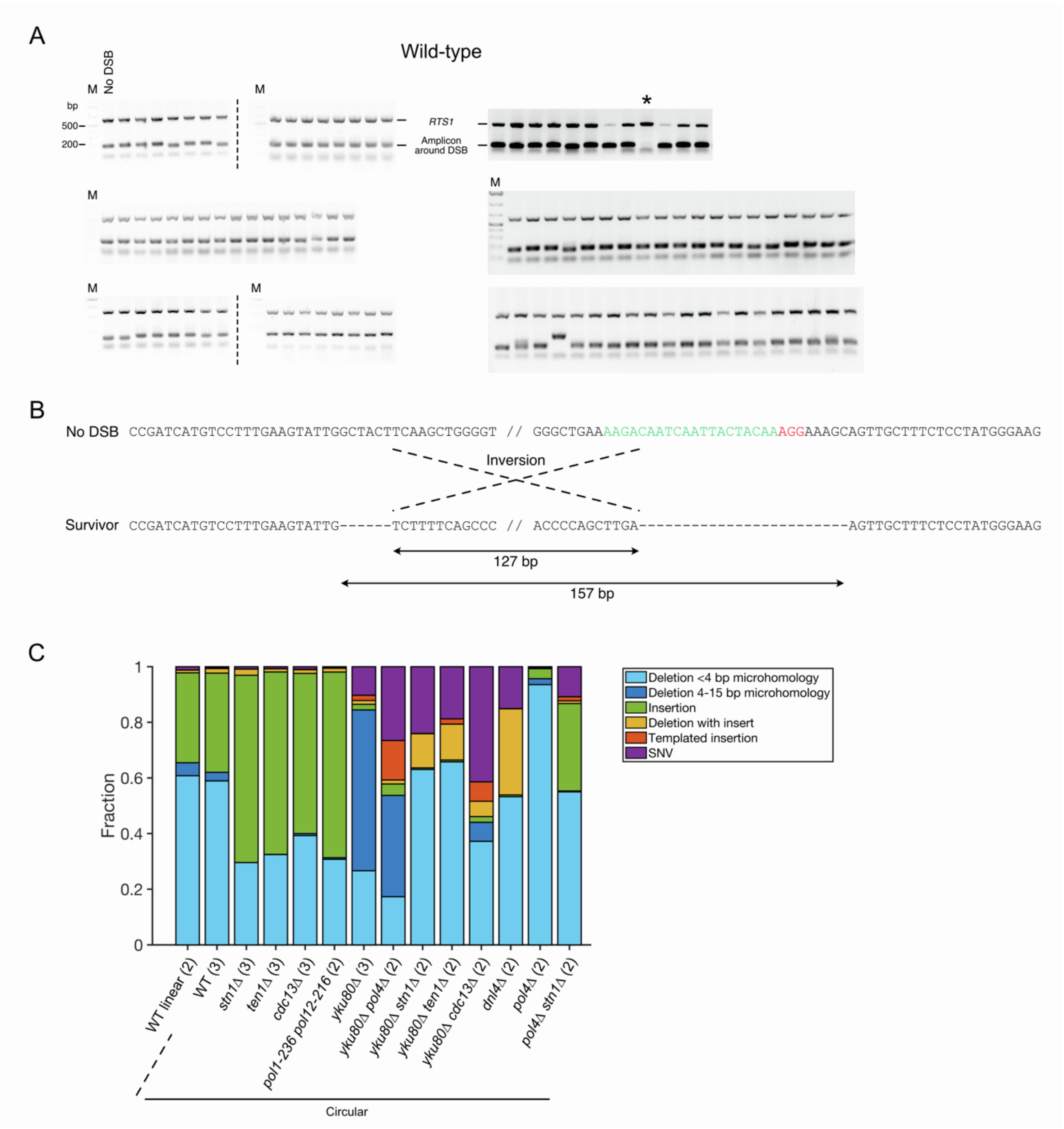

Supplemental Figure S2. Sequencing of an amplicon around DSB captures nearly all repair event. Related to Figure 2.

(A) Multiplex PCR showing a 176-bp fragment around the DSB ("Amplicon around DSB") and a fragment in *RTS1*. *: unproductive PCR around the DSB. M: molecular weight marker.

(B) Schematic representation of the single event not captured by PCR in (A). PCR mapping and sequencing of the junction revealed an inversion.

(C) Mutation signature of the indicated strains as shown in Figure 2A, but represented in fraction of each type, without normalization by survival rate.

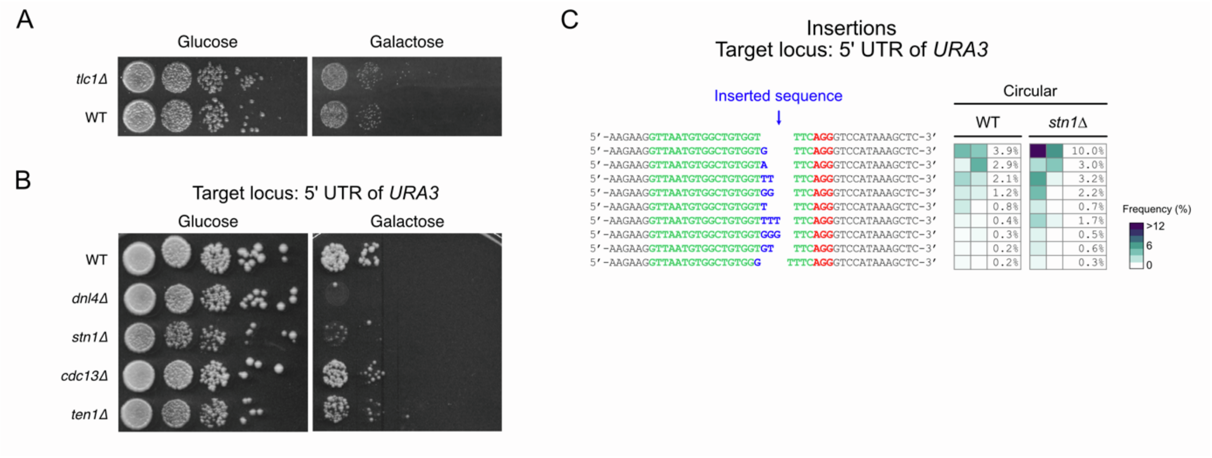

Supplemental Figure S3. CST's role is telomerase-independent and verified at another DSB locus. Related to Figure 3.

(A) Spot assay as in Figure 1D with the indicated strains. The image comes from the same plate as Figure 5A and thus shares the same WT.

(B) Spot assay as in Figure 1D with the indicated strains and with Cas9 targeted to the 5' UTR of *URA3* with a specific guide RNA.

(C) Heatmap of the frequency of each insertion outcome for Cas9 targeted at the 5' UTR of *URA3*, for individual experiments in WT and *stn1Δ* strains. Representation as in Figure 2B, except that only events with frequency > 0.002 are shown. See Supplemental Data 1 and 2 for the unfiltered data.

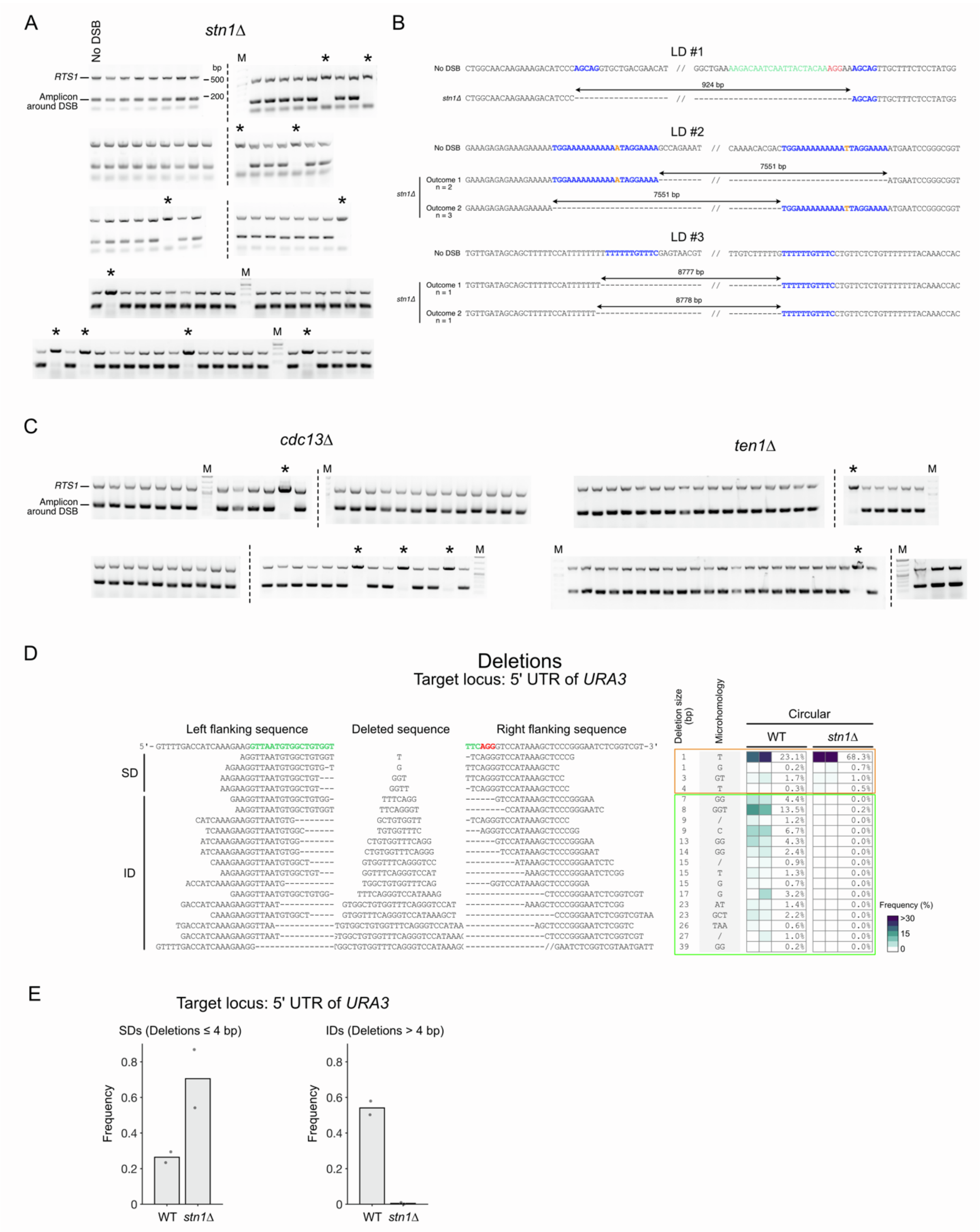

Supplemental Figure S4. LDs in CST mutants and mutation signature analysis at the DSB targeted to the 5’ UTR of *URA3*. Related to Figure 4.

(A) Multiplex PCR in *stn1Δ* strain showing a 176-bp fragment around the DSB (“Amplicon around DSB”) and a fragment in *RTS1*. M: molecular weight marker. * indicate unproductive PCRs around the cut site.

(B) Sequences at the junctions of 8 large deletions detected in (A), revealing LDs of 4 different sizes. The microhomologies used are shown in blue. For LD #2, the microhomology is 22-bp long with a mismatch (in orange). Mismatch repair would eventually resolve the mispairing, leading to the 2 observed outcomes. For LD #3, additional deletion of 1 or 2 bp leads to the 2 observed outcomes.

(C) Multiplex PCR as in (A) but performed on surviving colonies of *cdc13Δ* and *ten1Δ* mutants.

(D) Heatmap of the frequency of each repair outcome with a deletion for individual experiments (in columns) with the indicated strains, for Cas9 targeted at the 5’UTR of *URA3*. Only deletions that appear with a frequency of > 0.018 in at least one experiment are shown. See Supplemental Data 1 and 2 for the unfiltered data.

(E) Frequency of SDs (left) and IDs (right) for WT and *stn1Δ*, derived from (C), when the DSB is induced at the 5’UTR of *URA3*. Each dot represents an independent experiment.

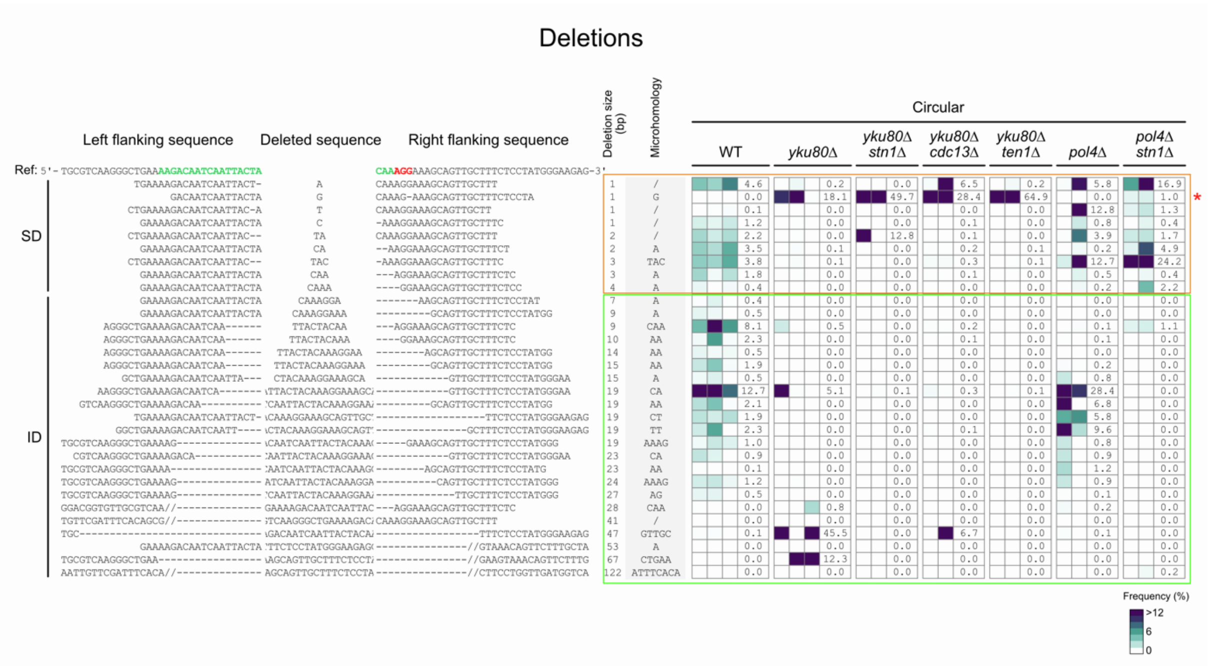

Supplemental Figure S5. CST is required for IDs present in *yku80Δ* and *pol4Δ* mutants. Related to Figure 4.

Heatmap of the frequency of each repair outcome with a deletion for individual experiments (in columns) with the indicated strains. For each strain, a column indicates the average frequency (in %) of each deletion. Red asterisk: deletion of one G located 4-5 bp away from the cut site, likely not due to the repair of the DSB. The data for the WT, *yku80Δ* and *pol4Δ* strains are reused from Figure 2C, but with a different frequency scale. See Supplemental Data 1 and 2 for the unfiltered data.

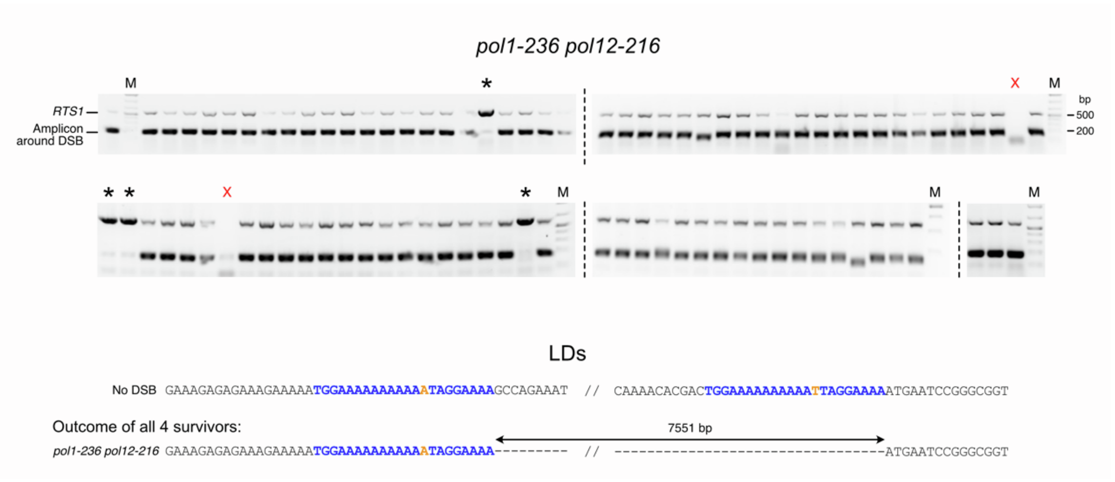

Supplemental Figure S6. Multiplex PCR assay in *pol1-236 pol12-216* mutant. Related to Figure 4.

Multiplex PCR in *pol1-236 pol12-216* strain showing a 176-bp fragment around the DSB ("Amplicon around DSB") and a fragment in *RTS1*. * indicate unproductive PCRs around the DSB. M: molecular weight marker. Red Xs show failed PCR amplification for the control site; the corresponding samples are thus removed from analysis. (Lower part) All 4 unproductive PCRs corresponded to the same LD as in outcome 1 of LD #2 in *stn1Δ* survivor clones, in Figure S4B.

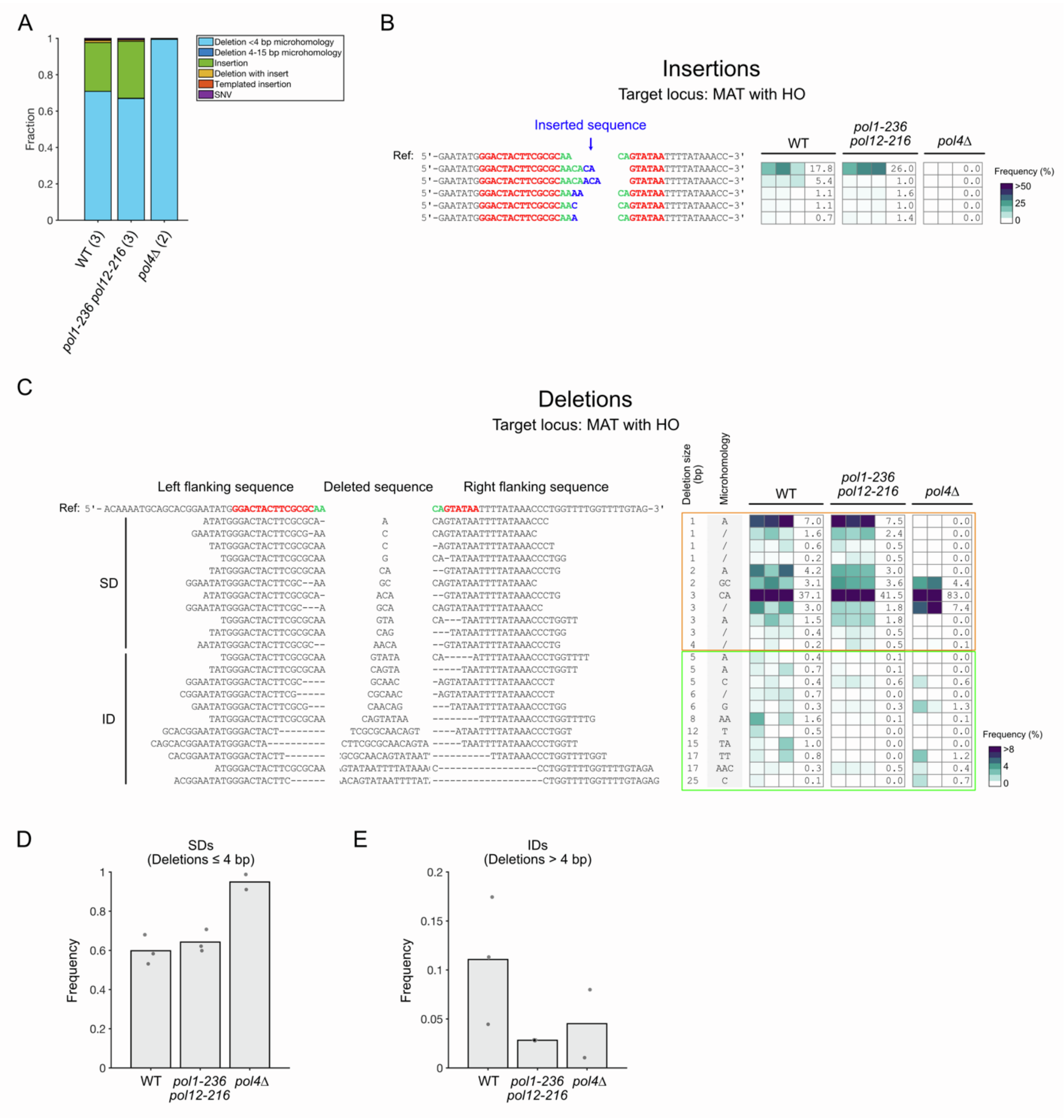

Supplemental Figure S7. CST's interaction with Polα-primase specifically affects the IDs formed after repair of an HO cut. Related to Figure 6.

(A) Mutation signature of the indicated strains in the JKM179 background allowing the induction of an HO cut at the MAT locus. Representation as in Supplemental Figure S2C.

(B) Heatmap of the frequency of each repair outcome after an HO cut with an insertion for individual experiments (in columns) with the indicated strains, represented as in Figure 2B. For each strain, a column indicates the average frequency (in %) of each insertion. HO's recognition site is colored in red

and green, with the green part corresponding to the staggered cut region. See Supplemental Data 1 and 2 for the unfiltered data.

(C) Heatmap of the frequency of each repair outcome after an HO cut with a deletion for individual experiments (in columns) with the indicated strains, represented as in Figure 2C, except for the color bar which uses a different scaling. For each strain, a column indicates the average frequency (in %) of each deletion. See Supplemental Data 1 and 2 for the unfiltered data.

(D) Frequency of SDs, *i.e.* deletions ≤ 4 bp, for the indicated strains. Each dot represents an independent experiment.

(E) Frequency of IDs, *i.e.* 5-85 bp deletions, for the indicated strains.

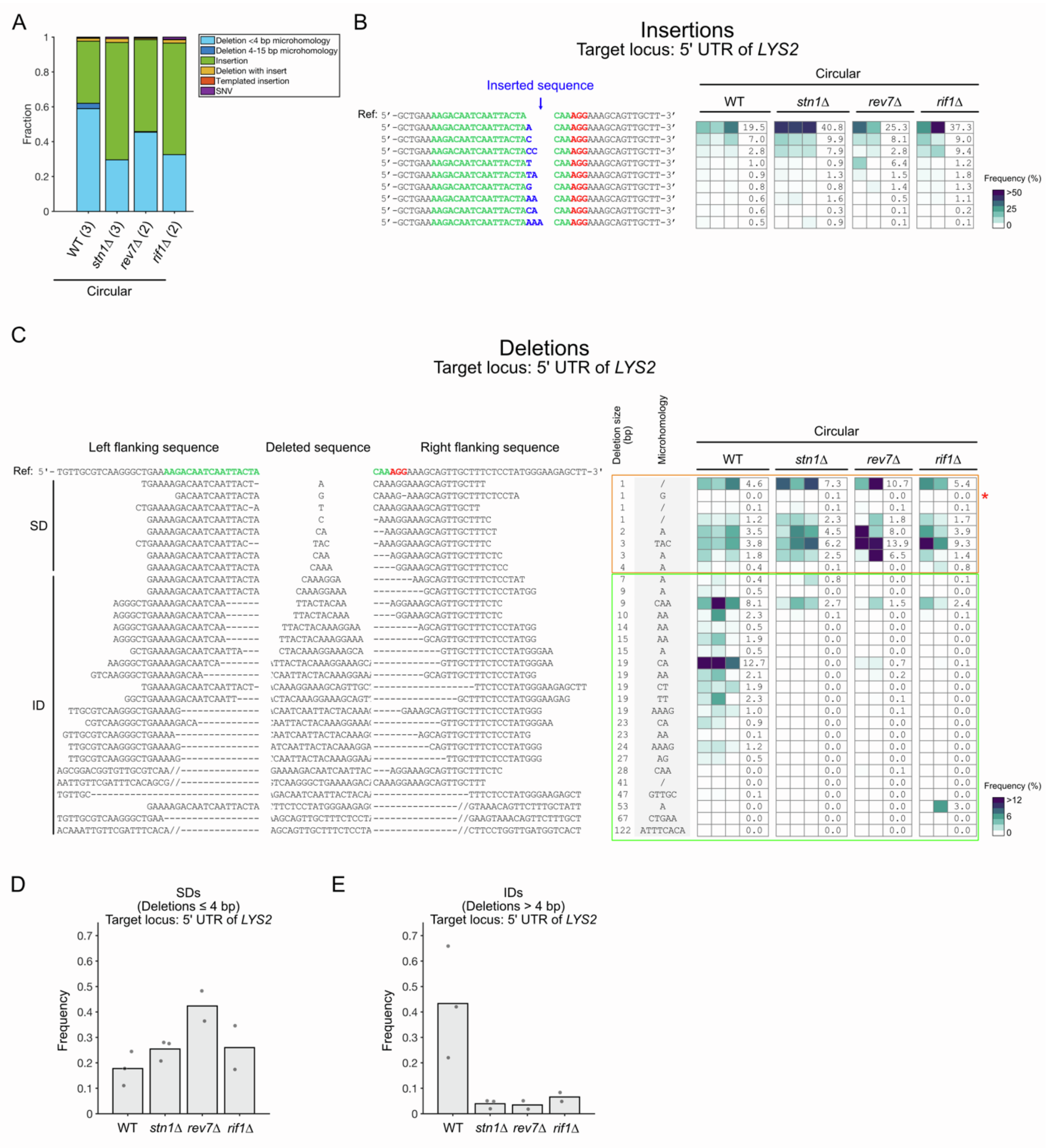

Supplemental Figure S8. Rev7 and Rif1 participate in ID formation. Related to Figure 6.

(A) Mutation signature of the indicated strains in the circular chromosome strain background. Representation as in Supplemental Figure S2C.

(B) Heatmap of the frequency of each repair outcome with an insertion for individual experiments (in columns) with the indicated strains, represented as in Figure 2B. For each strain, a column indicates the average frequency (in %) of each insertion. Data for WT and *stn1Δ* are reused from Figure 3C. See Supplemental Data 1 and 2 for the unfiltered data.

(C) Heatmap of the frequency of each repair outcome with a deletion for individual experiments (in columns) with the indicated strains, represented as in Figure 2C, except for the color bar which uses a different scaling. For each strain, a column indicates the average frequency (in %) of each deletion. Data for WT and *stn1Δ* are reused from Figure 4C. See Supplemental Data 1 and 2 for the unfiltered data.

(D) Frequency of SDs, *i.e.* deletions ≤ 4 bp, for the indicated strains. Each dot represents an independent experiment.

(E) Frequency of IDs, *i.e.* 5-85 bp deletions, for the indicated strains.

Supplemental Table S1. List of strains. Related to STAR Methods.

Strain	Genotype	Figures	Reference
BY4742	*MATα his3Δ1 leu2Δ0 lys2Δ0 ura3Δ0*; 16 linear chromosomes	6, S1	Shao et al., 2018
SY13	*MATα his3Δ1 leu2Δ0 lys2Δ0 ura3Δ0*; 2 linear chromosomes	S1	Shao et al., 2018
SY14	*MATα his3Δ1 leu2Δ0 lys2Δ0 ura3Δ0*; single linear chromosome	1-3, 6, S1-S2	Shao et al., 2018
yZX168	*MATα his3Δ1 leu2Δ0 lys2Δ0 ura3Δ0*; single circular chromosome	1-6, S1-S6, S8	This work
yZX170	yZX168 *stn1::HIS3*	1-6, S2-S4, S8	This work
yZX226	yZX168 *cdc13::HIS3*	1-4, S2-S4	This work
yZX384	yZX168 *ten1:::HIS3*	1-4, S2-S4	This work
yZX207	yZX168 *dnl4::LEU2*	1-3, S2-S3	This work
yZX208	yZX168 *stn1::HIS3 dnl4::LEU2*	1, 3	This work
yZX285	yZX168 *tlc1::HIS3*	1, S3	This work
yZX283	yZX168 *pol4::HIS3*	1-2, S2, S5	This work
yZX274	yZX168 *yku80::LEU2*	1-2, 5, S2, S5	This work
yZX271	yZX168 *stn1::HIS3 yku80::LEU2*	1-4, S2, S5	This work
yZX292	yZX168 *pol1-D236N pol12-G325D*	1-2, 4-6, S2, S6	This work
yZX206	SY14 *dnl4::LEU2*	3	This work
yZX320	yZX168 *mre11-H125N*	5	This work
yZX321	yZX168 *stn1::HIS3 mre11-H125N*	5	This work
yZX408	yZX168 *sae2::LEU2*	5	This work
yZX415	yZX168 *stn1::HIS3 sae2::LEU2*	5	This work
yZX353	yZX168 *ChrII:460851-460853::LEU2[nt1-559] ChrII:480990-480988::LEU2[nt478-1089] **	5	This work
yZX368	yZX168 *stn1::HIS3 ChrII:460851-460853::LEU2[nt1-559] ChrII:480990-480988::LEU2[nt478-1089] **	5	This work
yZX455	yZX168 *pol1-D236N pol12-G325D ChrII:460851-460853::LEU2[nt1-559] ChrII:480990-480988::LEU2[nt478-1089] **	5	This work
yZX421	yZX168 *stn1::HIS3 pol1-D236N pol12-G325D*	6	This work
yZX308	SY14 *pol1-D236N pol12-G325D*	6	This work
yZX392	BY4742 *pol1-D236N pol12-G325D*	6	This work
yZX485	yZX168 *cdc13::HIS3 yku80::LEU2*	S2, S5	This work
yZX481	yZX168 *ten1::HIS3 yku80::LEU2*	S2, S5	This work
yZX483	yZX168 *pol4::HIS3 yku80::LEU2*	2, S2	This work
yZX490	yZX168 *pol4::HIS3 stn1::kanMX6*	S2, S5	This work
yZX486	yZX168 *rif1::LEU2*	S8	This work
yZX488	yZX168 *rev7::kanMX6*	S8	This work
JKM179	*MATα ura3-52 trp1::hisG' leu2-3,112 lys5 ade3::GAL10:HO hoΔ hmlΔ::ADE1 hmrΔ::ADE1 ade1-100MX6*	S7	Lee et al., 1998
yZX386	JKM179 *pol1-D236N pol12-G325D*	S7	This work
yKD2176	JKM179 *pol4::kanMX6 leu2-3::lacI-GFP-LEU2 4.4kb-MAT::lacOp-TRP1 NUP49::NUP49-mCherry-URA3*	S7	This work

Supplemental Table S2. List of plasmids. Related to START Methods.

Plasmid	Vector	Selection marker	Insert	Purpose	Reference for vector
pZX010	bRA66	*HPH1*	Guide RNA sequence: GTTAATGTGGCTGTGGTTTC	GAL1-driven Cas9 expression targeting 5' UTR of *URA3*	Anand et al. 2017
pZX013	bRA66	*HPH1*	Guide RNA sequence: AAGACAATCAATTACTACAA	GAL1-driven Cas9 expression targeting 5' UTR of *LYS2*	Anand et al. 2017
pZX026	pJH2970	*HIS3*	2 guide RNA sequences: AGCCATAATAGCATCCAGAT and TGAAACGCTGCCGTAAGCAG	Cas9 cut at subtelomeres of ChrX-R and ChrXVI-L for SY14 circularization	Anand et al. 2017
pRS426		*URA3*	None	Plasmid religation assay	Christianson et al. 1992

Supplemental Table S3. List of primers. Related to STAR Methods.

Primer name	Forward/Reverse	Sequence	Target locus	Use
oT1721	F	GGAAAGTTTCCACCAGACGCTAAGTGGTAGCCATAATAGCATCCACTTACGGCAGCGTTTCACTTTGTTTGGAGAACGGTTGTTAACTTG	Chimera between subtelomeres X-R and XVI-L.	Repair donor sequence for chromosome circularization
oT1722	R	CAAGTTAACAACCGTTCTCCAAACAAAGTGAAACGCTGCCGTAAGTGGATGCTATTATGGCTACCACTTAGCGTCTGGTGGAAACTTTCC	Chimera between subtelomeres X-R and XVI-L.	Repair donor sequence for chromosome circularization
oT1735	F	GCTTATTCTCAAATGGTGAC	XVI-L subtelomere (17.5 kb from end)	PCR to verify chromosome circularization and generate Southern blot probe
oT1736	R	ACTTCCCAATCATGAGGATC	X-R subtelomere (2.1 kb from end)	PCR to verify chromosome circularization and generate Southern blot probe
oZX513	F	CCACCTTGTCAGATTCGAAGG	Locus 0.95 kb away from Cas9 cut site at 5' UTR of LYS2	qPCR
oZX514	R	GCATTTACCGAAGTTTACTCCG	Locus 0.95 kb away from Cas9 cut site at 5' UTR of LYS2	qPCR
oT976	F	CTGGTATGTGTAAAGCCGGT	ACT1	qPCR
oT977	R	ACGTAGGAGTCTTTTTGACCCA	ACT1	qPCR
oZX108	F	CGCAACAGCCATCACAATCTC	RTS1	Control PCR for deletion mapping
oZX099	R	ATGTTCACCACATGAGCGTA	RTS1	Control PCR for deletion mapping
oZX526	F	AAGTATGCTCATCAATCGTTCGG	Flanking Cas9 cut site at 5' UTR of LYS2	Large deletion mapping by PCR; qPCR
oZX525	R	CAGACTTAGAAAGCTCTTCCCATA	Flanking Cas9 cut site at 5' UTR of LYS2	Large deletion mapping by PCR; qPCR
oZX641	F	CAAAGTGGTGATAGAGTTCA	Cas9 cut site at 5' UTR of LYS2 -652 bp	Large deletion mapping by PCR
oZX642	R	ACTGTAAATCAGCTGGCGTT	Cas9 cut site at 5' UTR of LYS2 -652 bp	Large deletion mapping by PCR
oZX664	F	TCAGATCGGATGTGCTTTA	Cas9 cut site at 5' UTR of LYS2 -3421 bp	Large deletion mapping by PCR
oZX665	R	GAGTGCTGTAAGGATTGT	Cas9 cut site at 5' UTR of LYS2 -3421 bp	Large deletion mapping by PCR
oZX666	F	TGCAGCTCTTTGGAACATG	Cas9 cut site at 5' UTR of LYS2 -4626 bp	Large deletion mapping by PCR
oZX667	R	ACTTGGCTCTCCATTGCTT	Cas9 cut site at 5' UTR of LYS2 -4626 bp	Large deletion mapping by PCR
oZX649	F	CCGTTCGACAGAACAAACC	Cas9 cut site at 5' UTR of LYS2 +1982 bp	Large deletion mapping by PCR
oZX650	R	GCACAGTTCTCCGACATT	Cas9 cut site at 5' UTR of LYS2 +1982 bp	Large deletion mapping by PCR
oZX639	F	TTCGACACTCCTTATTCAGGAC	Cas9 cut site at 5' UTR of LYS2 +2196 bp	Large deletion mapping by PCR
oZX640	R	AACGTCATTGCTCGGACATGT	Cas9 cut site at 5' UTR of LYS2 +2196 bp	Large deletion mapping by PCR
oZX651	F	CATGGGTAAAGAGAAGTCT	Cas9 cut site at 5' UTR of LYS2 +3235 bp	Large deletion mapping by PCR
oZX652	R	CTTCCACAAAGCAATATCGAT	Cas9 cut site at 5' UTR of LYS2 +3235 bp	Large deletion mapping by PCR
oZX425	F	AATGATACGGCGACCACCGAGATCTACACACACTCTTTCCCTACACGACGCTCTTCCGATCTTGCTCATCAATCGTTCGGAC	Flanking Cas9 cut site at 5' UTR of LYS2	P5 primer for Illumina sequencing.
oZX426-oZX435	R	CAAGCAGAAGACGGCATACGAGAT**[INDEX]**GTGACTGGAGTTCAGACGTGTGCTCTTCCGATCTTTCAGGCAGCAAGTGACCAT	Flanking Cas9 cut site at 5' UTR of LYS2	P7 primers for Illumina sequencing. INDEX indicates multiplexing barcode sequence.
oZX671	F	AATGATACGGCGACCACCGAGATCTACACACACTCTTTCCCTACACGACGCTCTTCCGATCTACCGAAGTTATCTGATGTAG	Flanking Cas9 cut site at 5' UTR of URA3	P5 primer for Illumina sequencing.
oZX672-oZX677	R	CAAGCAGAAGACGGCATACGAGAT**[INDEX]**GTGACTGGAGTTCAGACGTGTGCTCTTCCGATCTGCCCCGTAAAATACTGTTAC	Flanking Cas9 cut site at 5' UTR of URA3	P7 primers for Illumina sequencing. INDEX indicates multiplexing barcode sequence.
pr-1850	F	AATGATACGGCGACCACCGAGATCTACACACACTCTTTCCCTACACGACGCTCTTCCGATCTCTCACAGTTTGGCTCCGGTG	Flanking HO cut site at MAT locus	P5 primer for Illumina sequencing.
pr-1851-1855	R	CAAGCAGAAGACGGCATACGAGAT**[INDEX]**GTGACTGGAGTTCAGACGTGTGCTCTTCCGATCTTAAACAACCTCCGCCACGAC	Flanking HO cut site at MAT locus	P7 primers for Illumina sequencing. INDEX indicates multiplexing barcode sequence.
