# Supplementary material for: The CST complex mediates a post-resection non-homologous end joining repair pathway and promotes local deletions in Saccharomyces cerevisiae
Source: Cell Genom. 2025 Jul 16;5(10):100947. doi: 10.1016/j.xgen.2025.100947 (PMC12791002; doi:10.1016/j.xgen.2025.100947)
Supplement: Data S3. The CST complex mediates a post-resection non-homologous end-joining repair pathway and promotes local deletions in Saccharomyces cerevisiae [file mmc4.pdf]

Cell Genomics
Transparent Peer Review Record

The CST complex mediates a post-resection non-homologous end-joining repair pathway and promotes local deletions in Saccharomyces cerevisiae

Author list: Oana Ilioaia, Liébaut Dudragne, Clémentine Brocas, Léa Meneu, Romain Koszul , Karine Dubrana & Zhou Xu

Summary

Initial submission:	Received : 2nd January 2025
	Scientific editor: Judith Nicholson
First round of review:	Number of reviewers: 2<br>Revision invited : January 17th 2025<br>Revision received : May 2nd 2025
Second round of review:	Number of reviewers: 2<br>Accepted : 16th June 2025
Data freely available:	Yes
Code freely available:	Yes

This transparent peer review record is not systematically proofread, type-set, or edited. Special characters, formatting, and equations may fail to render properly. Standard procedural text within the editor's letters has been deleted for the sake of brevity, but all official correspondence specific to the manuscript has been preserved.

Referees' reports, first round of review

Reviewer 1

The Cdc13-Stn1-Ten1 (CST) complex has well established roles at telomeres where it binds to single-stranded DNA (ssDNA), prevents excessive degradation by Exo1 and recruits the DNA Pol alpha-primase complex for fill-in synthesis of overhangs formed after replication or telomerase activity. Recent studies in metazoans indicate an additional role for CST and Pol alpha for fill-in synthesis of resected ends at chromosome internal double-strand breaks (DSBs); thus, facilitating repair by end joining. To date, no studies have shown a similar function for CST in yeast and it is thought that resection initiation commits cells to homology-directed repair.

The main impediment to studying the function of CST is that it is essential for cell viability. In this study, the authors used a previously published system to fuse all 16 linear chromosomes into a single circular chromosome to overcome the lethality caused by loss of telomerase or CST. Using this system, they systematically investigated the role of canonical NHEJ factors and CST in the repair of a Cas9-induced DSB. Although the authors found only a modest reduction in survival of stn1, ten1 and cdc13 mutants to a Cas9-induced DSB, they identified a unique mutational signature associated with CST-mediated end-joining repair. In WT cells, the major classes of events were small indels at the break site and deletions of 5-85 bp, large deletions (>200 bp) were rare. The intermediate size deletions (ID, 5-85 bp) involving 4-15 bp long microhomologies were NHEJ independent but IDs with <4 bp microhomologies at the junction required NHEJ. Cells lacking CST showed no change in the recovery of small indels but a 5-10 decrease in the ID events and an increase in the frequency of large deletions. This phenotype was also observed for the pol1-236 pol12-216 mutant, which is defective for interaction with CST. Importantly, the authors use a qPCR-based assay to show that CST and Pol alpha limit resection at a DSB.

The results from this study are convincing and provide the first evidence for CST function at DSBs to promote end-joining repair in yeast. Importantly, the findings challenge the view that once resection has initiated, then repair by NHEJ cannot occur.

Comments:

In mammalian cells, CST and Pol alpha function with 53BP1/Rad9, Rif1 and Rev7 to counteract end resection. Were rif1 and rev7 tested in this system and if so, do they show a similar mutational signature to stn1?

It is surprising that stn1 has a stronger phenotype than cdc13 and ten1. Is there any evidence for Stn1 recruitment to DSBs independent of Cdc13? Is Stn1 the main subunit that interacts with Pol alpha-primase? Can Stn1 over-expression suppress the cdc13 phenotype, i.e., restore IDs?

The images of plates are dark making it difficult to see clear differences in the number of surviving colonies between some strains. This is particularly problematic for the SSA assay shown in Figure 5. The graph indicates 10% and 50% survival for the WT and stn1 strains, respectively, but survival seems much worse looking at the plate. Could the contrast of the plates be improved or cells concentrated before diluting and plating to provide higher numbers of colonies at the lower dilutions. This concern also applies to some of the spot assays measuring survival to Cas9 expression. The difference between stn1 and NHEJ-deficient mutants, and for the double mutants compared with singles, would be more apparent if more cells were spotted on plates.

Minor comments:

1. Title and abstract: Since CST and Pol alpha have established roles in end protection and end joining in metazoans, it would be important to include in the title and abstract that the current study was performed in yeast.
2. The survival data for the Cas9 experiments are frequencies not rates.
3. Page 8, line 2: The Lemos et al reference should be cited here since they showed insertions at Cas9-induced DSBs in yeast require Pol4.
4. Figure 6: the title state that CST interaction with Pol alpha-primase is critical for ID/LD balance but no data are shown for LDs in the figure.

Reviewer 2

In this manuscript, the authors demonstrated that the Cdc13/Stn1/Ten1 (CST) complex acts after end resection to mediate a backup, imprecise NHEJ repair characterized by medium-sized deletions (5-85 bp). Using Pol1 and Pol12 mutants (defective in the interaction of Polα and CST), they further showed that the interaction between CST and Polα/primase is critical for this class of end joining, suggesting a role for fill-in DNA synthesis in facilitating this type of repair. Collectively, the study proposes a post-resection NHEJ repair mechanism that promotes medium-sized deletions while preventing larger deletions and rearrangements. While these findings are potentially interesting, more thorough analysis is required to fully support the proposed model and clarify the details of the repair process.

1. The data presentation in figure 2 is not sufficiently comprehensive. It is hard to link the percentage described in the text with specific indels in the figures. Instead of using light to dark blue color to indicate the indel frequency, the exact percentage should be presented (e.g., those above certain levels, such as >1% or >5%). In this sentence: "Surprisingly, the remaining IDs (40.2%) were not associated with significant microhomology (< 4 bp) and required Yku80 and Dnl4, indicating that they arose from NHEJ repair (Figure 2C)", I believe that 40.2% remaining IDs refer to those in yku80Δ cells, but they are also mentioned to be Yku80 dependent. Please clarify it. Please also indicate the percentage of the significant indels in Fig.4D.

2. Pol4 has been shown to play important roles in MMEJ in yeast (Lee and Lee, 2007; Meyer et al., 2015). In Fig.2C, the types of deletions accumulated in pol4Δ appear to be different from those in yku80Δ and dnl4Δ. It needs to be clarified whether the observations from pol4Δ in this study support the role of Pol4 in NHEJ (including imprecise NHEJ) or MMEJ. Indel analysis in pol4Δ/stn1Δ, pol4Δ/yku80Δ and pol4Δ/dln4Δ need to be performed.

3. The authors showed that combining stn1Δ with yku80Δ or dnl4Δ does not further decrease survival, leading to the conclusion that Stn1's contribution to the repair that is NHEJ-dependent. However, indel analysis showed that yku80Δ also accumulates relatively big deletions (47-67bp), whereas stn1Δ, cdc13Δ and ten1Δ mutants do not. In stn1Δ/yku80Δ mutant, no big deletions (47-67bp) are accumulated. This suggests that certain Ku-independent MMEJ mechanisms might depend on CST. Please clarify this point. Please also provide indel analysis of stn1Δ/dnl4Δ, ten1Δ/yku80Δ and ten1Δ/dnl4Δ.

4. Considering points 3 and 4, is it possible that CST is involved in both imprecise NHEJ (KU-dependent) and MMEJ (Ku independent)?

5. Complex and long-lived DSBs are more likely to induce end resection. Cas9 often remains bound to DSBs after cleavage, and this may promote more imprecise NHEJ and MMEJ. Use the HO endonuclease to induce DSBs and compare the repair outcomes in WT cells with those observed following Cas9 cleavage, and determine whether the repair outcomes of yku80Δ, dnl4Δ, pol4Δ, and stn1Δ mutants are same or different after Cas9 or HO cleavage.

6. Large deletions are found to accumulate in stn1Δ. Do similar deletions also accumulate in ten1Δ and cdc13Δ mutants?

7. The model that CST promotes end resection by recruiting Polα/primase to ssDNA overhangs for fill-in DNA synthesis was originally proposed in mammalian cells. The authors suggested that this mechanism is conserved in yeast, as evidenced by increased end resection in stn1Δ and in the Pol1 and Pol12 mutants defective in the Polα-CST interaction. To confirm that CST recruits Polα/primase to ssDNA overhangs at DSBs, ChIP analysis of Polα/primase and CST after Cas9 cleavage, as well as ChIP of Polα/primase in the stn1Δ strain, needs to perform.

8. Considering the roles of CST in end joining, do stn1Δ, cdc13Δ and ten1Δ mutants show sensitivity to IR like yku80Δ? Are Stn1, Cdc13 and Ten1 epistatic to Ku80 and Dnl4 in resisting IR?

9. Do chromosome rearrangements, such as translocations, increase in stn1Δ, cdc13Δ and ten1Δ mutants?

10. Ku shows high affinity to blunt ends or ends with short overhangs. How does Ku load onto resected ends?

References:

Lee, K., and Lee, S.E. (2007). Saccharomyces cerevisiae Sae2- and Tel1-dependent single-strand DNA formation at DNA break promotes microhomology-mediated end joining. Genetics 176, 2003-2014.

Meyer, D., Fu, B.X., and Heyer, W.D. (2015). DNA polymerases delta and lambda cooperate in repairing double-strand breaks by microhomology-mediated end-joining in Saccharomyces cerevisiae. Proc Natl Acad Sci U S A 112, E6907-6916.

Authors' response to the first round of review

Point-by-point response to reviewers' comments

Comments are in black and responses in blue. Additional figures for reviewers only are named "Figure_reviewer 1 and 2".

Reviewers' Comments:

Reviewer #1: The Cdc13-Stn1-Ten1 (CST) complex has well established roles at telomeres where it binds to single-stranded DNA (ssDNA), prevents excessive degradation by Exo1 and recruits the DNA Pol alpha-primase complex for fill-in synthesis of overhangs formed after replication or telomerase activity. Recent studies in metazoans indicate an additional role for CST and Pol alpha for fill-in synthesis of resected ends at chromosome internal double-strand breaks (DSBs); thus, facilitating repair by end joining. To date, no studies have shown a similar function for CST in yeast and it is thought that resection initiation commits cells to homology-directed repair.

The main impediment to studying the function of CST is that it is essential for cell viability. In this study, the authors used a previously published system to fuse all 16 linear chromosomes into a single circular chromosome to overcome the lethality caused by loss of telomerase or CST. Using this system, they systematically investigated the role of canonical NHEJ factors and CST in the repair of a Cas9-induced DSB. Although the authors found only a modest reduction in survival of stn1, ten1 and cdc13 mutants to a Cas9-induced DSB, they identified a unique mutational signature associated with CST-mediated end-joining repair. In WT cells, the major classes of events were small indels at the break site and deletions of 5-85 bp, large deletions (>200 bp) were rare. The intermediate size deletions (ID, 5-85 bp) involving 4-15 bp long microhomologies were NHEJ independent but IDs with <4 bp microhomologies at the junction required NHEJ. Cells lacking CST showed no change in the recovery of small indels but a 5-10 decrease in the ID events and an increase in the frequency of large deletions. This phenotype was also observed for the pol1-236 pol12-216 mutant, which is defective for interaction with CST. Importantly, the authors use a qPCR-based assay to show that CST and Pol alpha limit resection at a DSB.

The results from this study are convincing and provide the first evidence for CST function at DSBs to promote end-joining repair in yeast. Importantly, the findings challenge the view that once resection has initiated, then repair by NHEJ cannot occur.

We are grateful to the reviewer for the positive feedback and the constructive comments.

Comments:

In mammalian cells, CST and Pol alpha function with 53BP1/Rad9, Rif1 and Rev7 to counteract end resection. Were rif1 and rev7 tested in this system and if so, do they show a similar mutational signature to stn1?

We thank the reviewer for this insightful suggestion. We performed mutational signature analyses in *rif1*Δ and *rev7*Δ mutants using our experimental system and found mutation profiles very similar to those observed in CST mutants. These results have now been included in the revised manuscript (Fig. S8).

It is surprising that stn1 has a stronger phenotype than cdc13 and ten1. Is there any evidence for Stn1 recruitment to DSBs independent of Cdc13? Is Stn1 the main subunit that interacts with Pol alpha-primase? Can Stn1 over-expression suppress the cdc13 phenotype, i.e., restore IDs?

This is an interesting point. Despite our efforts, we do not have evidence that Stn1 is recruited to DSB independently of Cdc13 as the low abundance of Stn1 did not allow us to detect a significant enrichment of Stn1 at the DSB site (see response to reviewer #2 for details). Both Stn1 and Cdc13 interact with Polα, through Pol12 and Pol1, respectively (Grossi et al., 2004; Qi and Zakian, 2000).

Regarding suppression, we attempted overexpression of Stn1 in a *cdc13Δ* background, but it did not restore ID frequency (Figure_reviewer 1). This suggests that Stn1's function at DSBs likely depends on the full CST complex. In addition, overexpression of Stn1 in WT cells leads to a decrease in ID frequency, but without LDs (Figure_reviewer 1D), thus displaying a unique signature distinct from both WT and *stn1*Δ. We verified that the overexpression using a strong promoter (pTDH3) worked as expected at mRNA and protein levels (Figure_reviewer 1A), confirming that the phenotype was not due to a problem of expression.

This experiment thus reveals a complex dominant negative effect that would need to be studied further to draw solid conclusions. We thus did not include these results in the manuscript.

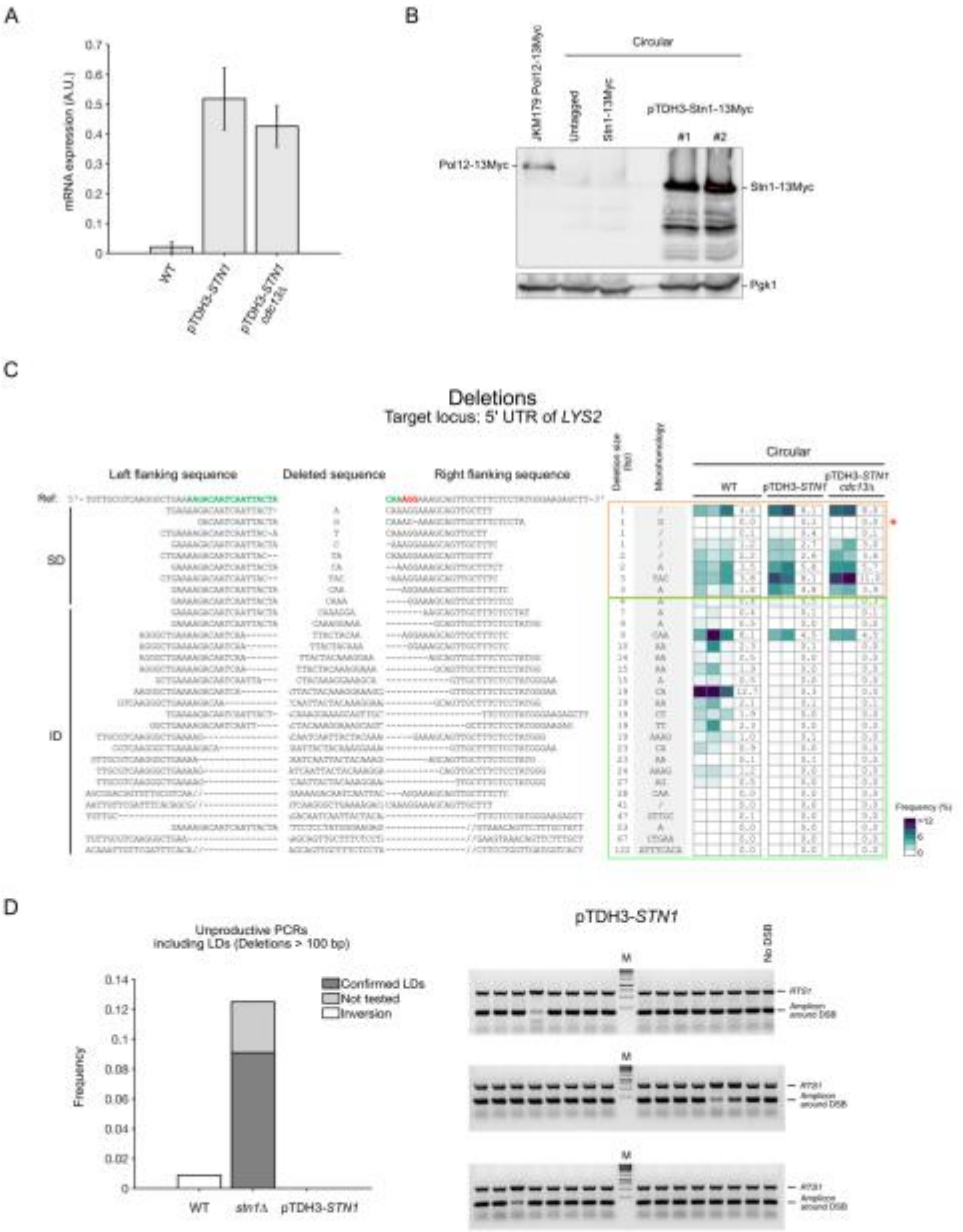

Figure_reviewer 1. Overexpression of Stn1 leads to a unique mutation signature with neither IDs nor LDs.

(A) Transcript levels (in arbitrary units) of *STN1* in WT and overexpressing strains where *STN1* is under the control of the strong promoter of *TDH3* are measured by RT-qPCR. A 20-25-fold increase is measured.

(B) Western blot using strains expressing a Myc-tagged Stn1. The endogenous Stn1 is not detectable due to low abondance. When Stn1-Myc is expressed under the control of the promoter of TDH3, a very strong signal is detected. A strain in which Pol12 is tagged is also

shown in this blot, which was used for our ChIP attempts (see response to reviewer #2). Of note, the mutation signature experiments were performed without Myc tag.
(C) Mutation signature analysis of the indicated strains, showing the deletions. Overexpressing Stn1 leads to decreased IDs.
(D) Analysis of LD frequency in the strain overexpressing Stn1 compared to WT and *stn1*Δ (left panel). No unproductive PCR was observed out of 47 surviving colonies tested (right panel).

The images of plates are dark making it difficult to see clear differences in the number of surviving colonies between some strains. This is particularly problematic for the SSA assay shown in Figure 5. The graph indicates 10% and 50% survival for the WT and stn1 strains, respectively, but survival seems much worse looking at the plate. Could the contrast of the plates be improved or cells concentrated before diluting and plating to provide higher numbers of colonies at the lower dilutions. This concern also applies to some of the spot assays measuring survival to Cas9 expression. The difference between stn1 and NHEJ-deficient mutants, and for the double mutants compared with singles, would be more apparent if more cells were spotted on plates.

We have now increased the brightness and contrast for all spot assay images, and hope that the differences between strains are now more apparent. We agree that growth on spot assays is a compound effect of survival, growth rate and repair kinetics. To directly assess survival, we also performed quantitative survival assays based on normalized colony counts (see Fig. 1C), which are unaffected by growth rate differences. We believe that providing both visual spot assays and quantitative survival assays supports well the effects we describe.

For the SSA assay, we engineered the circular strain to place two *LEU2* repeats 20 kb apart, maximizing the resection length needed for repair and amplifying the impact of increased end resection. The resulting SSA product deletes several non-essential genes, significantly impairing cell growth, especially in minimal media lacking leucine. These growth defects contribute to the slow-growth colony appearance on spot assays but are accounted for by our quantitative survival assays, which robustly confirm our conclusions.

Minor comments:

1. Title and abstract: Since CST and Pol alpha have established roles in end protection and end joining in metazoans, it would be important to include in the title and abstract that the current study was performed in yeast.

We have now added "in *Saccharomyces cerevisiae*" in both the title and the abstract.

2. The survival data for the Cas9 experiments are frequencies not rates.

We have corrected all instances of "rate" to "frequency" throughout the manuscript and figure legends.

3. Page 8, line 2: The Lemos et al reference should be cited here since they showed insertions at Cas9-induced DSBs in yeast require Pol4.

Thank you for pointing this out. We have now cited Lemos et al. in the relevant section.

4. Figure 6: the title state that CST interaction with Pol alpha-primase is critical for ID/LD balance but no data are shown for LDs in the figure.

Thanks for pointing this out. We removed "LD" from the title of Figure 6.

Reviewer #2: In this manuscript, the authors demonstrated that the Cdc13/Stn1/Ten1 (CST) complex acts after end resection to mediate a backup, imprecise NHEJ repair characterized by medium-sized deletions (5-85 bp). Using Pol1 and Pol12 mutants (defective in the interaction of Polα and CST), they further showed that the interaction between CST and Polα/primase is critical for this class of end joining, suggesting a role for fill-in DNA synthesis in facilitating this type of repair. Collectively, the study proposes a post-resection NHEJ repair mechanism that promotes medium-sized deletions while preventing larger deletions and rearrangements. While these findings are potentially interesting, more thorough analysis is required to fully support the proposed model and clarify the details of the repair process.

We thank the reviewer for their thoughtful and constructive comments, which have significantly improved the clarity and strength of our manuscript.

1. The data presentation in figure 2 is not sufficiently comprehensive. It is hard to link the percentage described in the text with specific indels in the figures. Instead of using light to dark blue color to indicate the indel frequency, the exact percentage should be presented (e.g., those above certain levels, such as >1% or >5%). In this sentence: "Surprisingly, the remaining IDs (40.2%) were not associated with significant microhomology (< 4 bp) and required Yku80 and Dnl4, indicating that they arose from NHEJ repair (Figure 2C)", I believe that 40.2% remaining IDs refer to those in yku80Δ cells, but they are also mentioned to be Yku80 dependent. Please clarify it. Please also indicate the percentage of the significant indels in Fig.4D.

We have revised Fig. 2B-C and Fig. 4D to include average percentage of each mutational event per strain, as suggested. To preserve readability, individual replicates columns retain color coding, and full quantitative data are provided in Supplemental Data 1 (and 2) and raw data deposited in the European Nucleotide Archive (ENA).

We acknowledge that our initial phrasing caused confusion. All the percentages mentioned are relative to all mutations and not to specific categories. We realize that phrasing such as "the remaining IDs (40.2%)" might therefore be confusing. We now add "of all mutations" to clarify this wherever ambiguity might arise.

The 40.2% (of all mutations in WT) refers to IDs in WT that are not associated with microhomologies. In *yku80Δ* mutant, these IDs are no longer present and are therefore Yku80-dependent in WT cells. In *yku80Δ*, only IDs with microhomologies are still observed and represent 57.9% of all mutations in *yku80Δ* (whereas they represent 3.1% in the WT).

2. Pol4 has been shown to play important roles in MMEJ in yeast (Lee and Lee, 2007; Meyer et al., 2015). In Fig.2C, the types of deletions accumulated in pol4Δ appear to be different from those in yku80Δ and dnl4Δ. It needs to be clarified whether the observations from pol4Δ in this study support the role of Pol4 in NHEJ (including imprecise NHEJ) or MMEJ. Indel analysis in pol4Δ/stn1Δ, pol4Δ/yku80Δ and pol4Δ/dln4Δ need to be performed.

We agree that a more precise assessment of Pol4's role is valuable. While characterizing Pol4 is not the primary focus of our study, we now include new mutation signature analyses for *pol4*Δ *stn1*Δ and *pol4*Δ *ku80*Δ double mutants. As previously described by others, we observed a complete disappearance of NHEJ mediated insertions, supporting the contribution of Pol4 to these repair events (Fig. 2A, green category). In *pol4*Δ, we also observed a marked decrease in deletions with few or no homology (Figure 2A, light blue category) that are also strongly decreased in *yku80*Δ and *dnl4*Δ mutants, supporting a role for Pol4 in the production of NHEJ-mediated deletions.

In addition, we observed an overall decrease in deletions mediated by microhomologies in absence of Pol4 (Fig. 2A, dark blue category), in contrast to *yku80*Δ in which they are maintained, supporting a role for Pol4 in MMEJ. In *pol4*Δ *yku80*Δ, the subset of Pol4-independent deletions were virtually all eliminated, confirming their reliance on NHEJ (Fig. 2C). Conversely, the two major Ku-independent deletions of 47-67 bp associated with microhomologies of 5 bp were eliminated, implicating Pol4 in these MMEJ-mediated events. Nevertheless, a 122-bp deletion using 8 bp of microhomology observed in one of the replicates of *pol4*Δ *yku80*Δ suggests that some MMEJ events can persist without Pol4. The exact mechanisms underlying this selectivity remain to be defined.

Together, these results confirm that Pol4 is required for most NHEJ-mediated insertions and a significant subset of deletions via both NHEJ and MMEJ.

In *pol4*Δ *stn1*Δ, SDs similar to those observed in *pol4*Δ were preserved while Pol4-independent IDs were completely eliminated, showing that this subset of events requires Stn1 in the absence of Pol4 (Fig. S5) and confirming the general role of Stn1 in mediating IDs.

3. The authors showed that combining stn1Δ with yku80Δ or dnl4Δ does not further decrease survival, leading to the conclusion that Stn1's contribution to the repair that is NHEJ-dependent. However, indel analysis showed that yku80Δ also accumulates relatively big deletions (47-67bp), whereas stn1Δ, cdc13Δ and ten1Δ mutants do not. In stn1Δ/yku80Δ mutant, no big deletions (47-67bp) are accumulated. This suggests that certain Ku-independent MMEJ mechanisms might depend on CST. Please clarify this point. Please also provide indel analysis of stn1Δ/dnl4Δ, ten1Δ/yku80Δ and ten1Δ/dnl4Δ.

Survival assays indicated that *stn1*Δ is epistatic to *yku80*Δ and *dnl4*Δ, supporting CST's role in NHEJ (Fig. 1C). However, mutational signatures reveal that some CST-dependent IDs are Ku-independent and associated with microhomologies, suggesting involvement in MMEJ as well (Fig. S5). Given the low frequency of these MMEJ events, representing 3.1 % of all mutation in the WT, their absence would not significantly affect survival and our epistasis analysis based on survival.

To investigate this further, we performed additional analyses in analyses in *yku80*Δ *ten1*Δ and *yku80*Δ *cdc13*Δ mutants. IDs of 47-67 bp present in *yku80*Δ were strongly reduced or absent in these double mutants, reinforcing the conclusion that CST contributes to the generation of IDs through both NHEJ and MMEJ.

Overall, these new data confirm that CST contributes to DSB repair, mostly through NHEJ and in a minor fraction, through MMEJ. We now clarify in the manuscript that CST's role is best described as promoting intermediate deletions (IDs), regardless of whether they arise via

NHEJ or MMEJ, likely through its influence on ssDNA levels. CST regulates the extent of ssDNA and therefore controls a subset of NHEJ-dependent IDs and MMEJ-dependent IDs. However, the LDs found in *stn1*Δ or in *pol1-236 pol12-216* mutants are mediated by microhomologies and demonstrate that CST does not directly affect MMEJ repair per se, but regulates the size of deletions.

4. Considering points 3 and 4, is it possible that CST is involved in both imprecise NHEJ (KU-dependent) and MMEJ (Ku independent)?

Yes, our findings support CST's involvement in both pathways. CST appears to act upstream of NHEJ and MMEJ repair and to regulate the extent of resection, stabilizing DNA ends to favor the generation of IDs. Since both NHEJ and MMJ can produce IDs, CST contribute to both pathways, but not all events within each pathway are CST-dependent. We have revised the manuscript to reflect this more nuanced interpretation.

5. Complex and long-lived DSBs are more likely to induce end resection. Cas9 often remains bound to DSBs after cleavage, and this may promote more imprecise NHEJ and MMEJ. Use the HO endonuclease to induce DSBs and compare the repair outcomes in WT cells with those observed following Cas9 cleavage, and determine whether the repair outcomes of yku80Δ, dnl4Δ, pol4Δ, and stn1Δ mutants are same or different after Cas9 or HO cleavage.

We thank the reviewer for this suggestion. We performed mutation signature analysis after HO induced DSB in the JKM179 strain (Lee et al., 1998). As with Cas9-induced DSBs, the signature was dominated by short insertions and deletions, with about 11% of mutations being IDs. In the *pol1-236 pol12-216* mutant, these IDs were reduced 4 folds, while SDs and insertions were unaffected, paralleling our Cas9-based findings.

We also analyzed *pol4*Δ in this system and confirmed its requirement for all insertions and some deletions, consistent with Cas9 data. Together, these results show that our conclusions extend to HO-induced breaks as well.

6. Large deletions are found to accumulate in stn1Δ. Do similar deletions also accumulate in ten1Δ and cdc13Δ mutants?

Yes. We have now included data showing that large deletions (>1kb) occur at 4.1% and 8% in *ten1*Δ and *cdc13*Δ mutants, respectively. The increase in *cdc13*Δ was statistically significant (Fisher's exact test, p-value = 0.0077). These results are now shown in Fig. 4A and S4C and discussed accordingly.

7. The model that CST promotes end resection by recruiting Polα/primase to ssDNA overhangs for fill-in DNA synthesis was originally proposed in mammalian cells. The authors suggested that this mechanism is conserved in yeast, as evidenced by increased end resection in stn1Δ and in the Pol1 and Pol12 mutants defective in the Polα-CST interaction. To confirm that CST recruits Polα/primase to ssDNA overhangs at DSBs, ChIP analysis of Polα/primase and CST after Cas9 cleavage, as well as ChIP of Polα/primase in the stn1Δ strain, needs to perform.

We performed ChIP experiments using 13xMyc- or 3xHA-tagged Stn1, Cdc13 and Pol12 after Cas9 or HO-induced DSBs. Unfortunately, enrichment at DSBs was weak and inconsistent across replicates. Stn1 levels are low (Figure_reviewer 1B), making detection

difficult (as previously noted in (Grossi et al., 2004) for example), and Pol12 ChIP signals were dominated by replication-related binding. Overall, CST may bind transiently to DSB and only in a subset of cells at a given time, rendering its detection by ChIP difficult.

Therefore, more refined synchronization and optimized protocols would be necessary to detect transient CST/Polα recruitment to DSBs. However, our resection assays (Fig. 5) and functional interaction data (e.g., *pol1-236 pol12-216*) strongly support CST/Polα cooperation.

8. Considering the roles of CST in end joining, do stn1Δ, cdc13Δ and ten1Δ mutants show sensitivity to IR like yku80Δ? Are Stn1, Cdc13 and Ten1 epistatic to Ku80 and Dnl4 in resisting IR?

As previously reported, *S. cerevisiae* shows robust resistance to IR due to efficient homologous recombination, and neither *yku80*Δ nor *dnl4*Δ mutants exhibit increased sensitivity to IR or radiomimetic drugs (Mages et al., 1996; Siede et al., 1996; Teo and Jackson, 1997).

We nonetheless tested *dnl4*Δ, *stn1*Δ, or *stn1*Δ *dnl4*Δ in spot assays with MMS, zeocin and camptothecin (Figure_reviewer 2). While the circular chromosome strain has increased sensitivity to these drugs compared to linear genome strains (as previously noted in (Shao et al., 2019)), these results revealed no increased sensitivity relative to WT in our circular chromosome strain, in agreement with previous literature. Given their limited insight, we did not include these results in the manuscript.

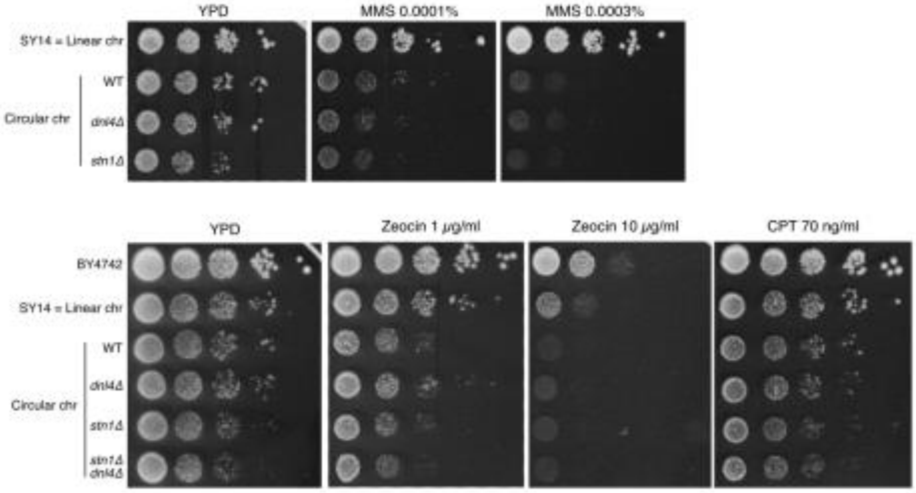

Figure_reviewer 2. Sensivity to radiomimetic drugs. Spot assays with the indicated strains either on control YPD media or in YPD containing the indicated drug.

9. Do chromosome rearrangements, such as translocations, increase in stn1Δ, cdc13Δ and ten1Δ mutants?

We agree this is an important question, though it goes beyond the scope of the current study. Adding the necessary rearrangement reporter system (e.g., *CAN1-URA3)* to our engineered strains would require significant effort.

We did perform long-read Nanopore sequencing of two *stn1*Δ survivors clones with large deletion (~ 8.8 kb), and we did not detect additional structural variations. Although this is a limited sample, it suggests that DSB repair in *stn1*Δ does not necessarily promote widespread genome rearrangements.

10. Ku shows high affinity to blunt ends or ends with short overhangs. How does Ku load onto resected ends?

As detailed in (Zahid et al., 2021), Ku can bind DNA ends, with short ssDNA overhangs and accommodate up to 15-20 nt of ssDNA. Because Ku can thread inward along DNA, it could potentially even load onto longer ssDNA overhangs, especially as these ends are degraded over time (Frank-Vaillant and Marcand, 2002; Zierhut and Diffley, 2008). IDs size of <~120 bp would require Ku to thread into a maximum of 60 nt of ssDNA on each side of the break, which seems plausible considering Ku can contact 25 bp of DNA.

Furthermore, Polα primase activity may generate RNA/DNA hybrids that limits resection and create dsDNA patches accessible to Ku. Ku has also been reported to bind such hybrid structures. Although the exact mechanism of Ku binding leading to IDs remains to be identified, the current understanding of how Ku binds to DNA, including ssDNA, is consistent with the generation of IDs by NHEJ post resection.

We now clarify in the discussion that Ku loading onto partially resected ends is compatible with known structural and biochemical data.

References

Frank-Vaillant, M., and Marcand, S. (2002). Transient stability of DNA ends allows nonhomologous end joining to precede homologous recombination. Mol. Cell *10*, 1189-1199.
Grossi, S., Puglisi, A., Dmitriev, P.V., Lopes, M., and Shore, D. (2004). Pol12, the B subunit of DNA polymerase alpha, functions in both telomere capping and length regulation. Genes Dev. *18*, 992-1006.
Lee, S.E., Moore, J.K., Holmes, A., Umezu, K., Kolodner, R.D., and Haber, J.E. (1998). Saccharomyces Ku70, mre11/rad50 and RPA proteins regulate adaptation to G2/M arrest after DNA damage. Cell *94*, 399-409.
Mages, G.J., Feldmann, H.M., and Winnacker, E.L. (1996). Involvement of the Saccharomyces cerevisiae HDF1 gene in DNA double-strand break repair and recombination. J. Biol. Chem. *271*, 7910-7915.
Qi, H., and Zakian, V.A. (2000). The Saccharomyces telomere-binding protein Cdc13p interacts with both the catalytic subunit of DNA polymerase alpha and the telomerase-associated est1 protein. Genes Dev. *14*, 1777-1788.
Shao, Y., Lu, N., Cai, C., Zhou, F., Wang, S., Zhao, Z., Zhao, G., Zhou, J.Q., Xue, X., and Qin, Z. (2019). A single circular chromosome yeast. Cell Res. *29*, 87-89.

Siede, W., Friedl, A.A., Dianova, I., Eckardt-Schupp, F., and Friedberg, E.C. (1996). The Saccharomyces cerevisiae Ku autoantigen homologue affects radiosensitivity only in the absence of homologous recombination. Genetics *142*, 91-102.
Teo, S.H., and Jackson, S.P. (1997). Identification of Saccharomyces cerevisiae DNA ligase IV: involvement in DNA double-strand break repair. EMBO J. *16*, 4788-4795.
Zahid, S., Seif El Dahan, M., Iehl, F., Fernandez-Varela, P., Le Du, M.H., Ropars, V., and Charbonnier, J.B. (2021). The Multifaceted Roles of Ku70/80. Int J Mol Sci *22*.
Zierhut, C., and Diffley, J.F. (2008). Break dosage, cell cycle stage and DNA replication influence DNA double strand break response. EMBO J. *27*, 1875-1885.

Referees' report, second round of review

Reviewer 1

This is an important and timely study showing conservation of the Rif1-Rev7-CST-Pol alpha pathway for fill-in synthesis at resected double-strand breaks to promote their repair by end joining. The authors responded well to the reviewer's comments and I have no further concerns to be addressed.

Reviewer 2

The authors have adequately addressed reviewers' comments.

Authors' response to the second round of review

N/A
