# Supplementary material for: The CST complex mediates a post-resection non-homologous end joining repair pathway and promotes local deletions in Saccharomyces cerevisiae
Source: Cell Genom. 2025 Jul 16;5(10):100947. doi: 10.1016/j.xgen.2025.100947 (PMC12791002; doi:10.1016/j.xgen.2025.100947)
Supplement: Document S2. Article plus supplemental information [file mmc5.pdf]

Cell Genomics

Article

The CST complex mediates a post-resection non-homologous end joining repair pathway and promotes local deletions in *Saccharomyces cerevisiae*

Graphical abstract

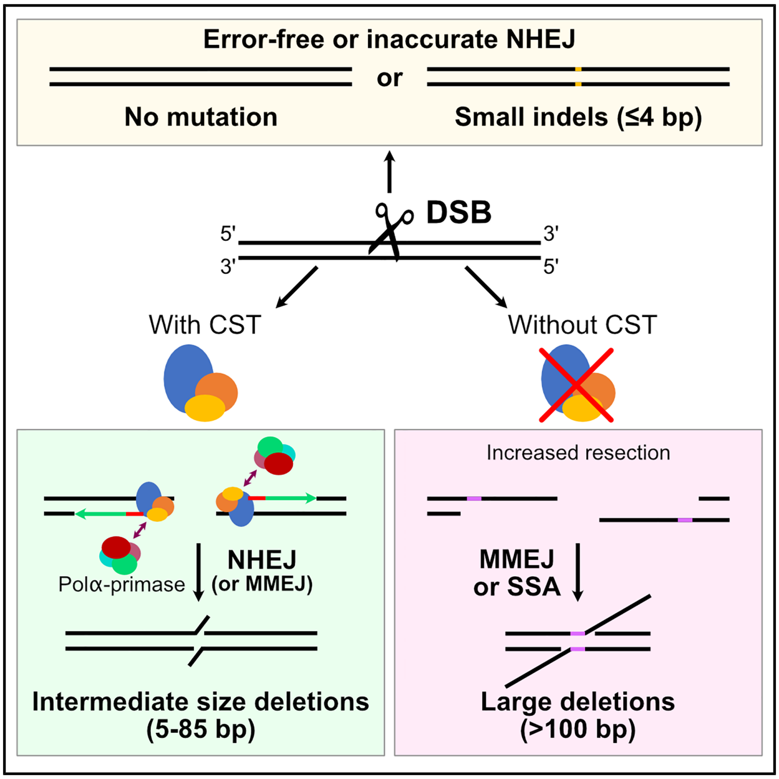

Highlights

- NHEJ can repair a DSB after resection, leading to local deletions of 5–85 bp
- The CST complex in interaction with Polα-primase promotes these deletions
- CST/Polα-primase limits resection and prevents even larger deletions
- CST plays a pivotal role in repair-pathway choice and genome stability

Authors

Oana Ilioaia, Liébaut Dudragne, Clémentine Brocas, Léa Meneu, Romain Koszul, Karine Dubrana, Zhou Xu

Correspondence

zhou.xu@sorbonne-universite.fr

In brief

Repair of DNA double-strand breaks is finely regulated to maintain genome integrity. Here, Ilioaia et al. demonstrate the contribution of the CST complex, known for its function at telomeres, to a back-up repair by non-homologous end joining, promoting local deletions while preventing larger, more deleterious deletions.

Ilioaia et al., 2025, Cell Genomics *5*, 100947
October 8, 2025 © 2025 The Author(s). Published by Elsevier Inc.
https://doi.org/10.1016/j.xgen.2025.100947

CellPress

Cell Genomics

CellPress
OPEN ACCESS

Article

The CST complex mediates a post-resection non-homologous end joining repair pathway and promotes local deletions in *Saccharomyces cerevisiae*

Oana Ilioaia,[1] Liébaut Dudragne,[1] Clémentine Brocas,[2] Léa Meneu,[3,4] Romain Koszul,[3] Karine Dubrana,[2] and Zhou Xu[1,5,*]
[1]Sorbonne Université, CNRS, UMR7238, Institut de Biologie Paris-Seine, Laboratory of Computational, Quantitative and Synthetic Biology, CQSB, Paris 75005, France
[2]Université Paris Cité, INSERM, CEA, Stabilité Génétique Cellules Souches et Radiations, Fontenay-aux-Roses 92260, France
[3]Institut Pasteur, CNRS UMR3525, Université Paris Cité, Unité Régulation Spatiale des Génomes, Paris 75015, France
[4]Sorbonne Université, Collège Doctoral, Paris, France
[5]Lead contact
*Correspondence: zhou.xu@sorbonne-universite.fr
https://doi.org/10.1016/j.xgen.2025.100947

SUMMARY

The repair of a DNA double-strand break (DSB) by non-homologous end joining (NHEJ) generally leaves an intact or minimally modified sequence. Resection exposes single-stranded DNA and directs repair toward homology-dependent pathways and away from NHEJ. Here, we report that in *Saccharomyces cerevisiae*, the Cdc13/Stn1/Ten1 (CST) complex, characterized for its telomeric functions, acts after resection initiation to mediate a back-up NHEJ repair. We found a CST-specific mutation signature after repair characterized by deletions of 5–85 bp that were mostly dependent on NHEJ, with a subset dependent on microhomology-mediated end joining (MMEJ). The interaction between CST and Polα-primase is critical for these intermediate-size deletions, suggesting a role for fill-in synthesis, thus limiting extensive resection, which would otherwise lead to MMEJ-dependent deletions of several kilobases. Collectively, these results depict a complex picture of repair pathway choice where CST facilitates post-resection NHEJ repair, promoting local deletions but guarding against larger and potentially more deleterious deletions and rearrangements.

INTRODUCTION

DNA damage, particularly double-strand breaks (DSBs), poses a major threat to genome integrity. To deal with this threat, the DNA damage response (DDR) is activated to ensure appropriate repair.[1,2] Following a DSB, an important step of the DDR is the processing of the break by resection of the 5′ extremities, which controls the repair pathway choice between two main repair mechanisms[3]: homologous recombination (HR), which requires single-stranded DNA (ssDNA) exposure for homology search and strand annealing, and non-homologous end joining (NHEJ), which directly ligates the DSB ends without extensive processing. Two other mechanisms distinct from HR and relying on sequence homology can join DSB ends: single-strand annealing (SSA) and microhomology-mediated end joining (MMEJ). Both require resection to expose the homologous sequences and lead to deletions but differ in several aspects of their molecular mechanisms and in their genetic requirements.

In contrast, telomeres, the natural extremities of eukaryotic linear chromosomes, resemble one side of a DSB but do not trigger a DDR, which would lead to inappropriate repair and genome instability.[4] They are thus protected by proteins bound to the double-stranded and the single-stranded parts of the telomere that inhibit the DDR. In *Saccharomyces cerevisiae*, the CST complex, composed of Cdc13/Stn1/Ten1, binds and protects the single-stranded $TG_{1–3}$ telomeric repeats, prevents Exo1 from resecting the 5′ strand, and recruits telomerase by direct interaction with Est1.[5–13] In addition, CST recruits Polα-primase for lagging strand synthesis to resynthesize the double-stranded DNA (dsDNA) after replication or telomerase activity.[12,14] Collectively, these roles prevent telomere loss and the associated chromosomal instability to preserve genome integrity.

While CST's role at telomeres is well established, its extra-telomeric functions are less well understood. The human CST complex (CTC1/STN1/TEN1) has been implicated in recovery and genome stability after replication stress by promoting new origin firing or stimulating POLα for replication restart.[15] The CST complex was also shown to stabilize and protect stalled replication forks.[16–18] Recently, the CST complex, together with Polα-primase, has been involved in DSB processing as an effector of the 53BP1-RIF1-Shieldin (SHLD1/SHLD2/SHLD3/REV7) axis, which controls resection and repair pathway choice, by performing fill-in synthesis on 3′ overhangs and facilitating NHEJ repair.[19–23] Additionally, CST was reported to promote DNA repair and survival in response to oxidative damage.[24,25]

In budding yeast, CST's interaction with Polα-primase was suggested to regulate transcription during replication.[26] Additionally, Cdc13 localizes at DSBs in a Mre11- and Rad51-dependent

© 2025 The Author(s). Published by Elsevier Inc.
This is an open access article under the CC BY license (http://creativecommons.org/licenses/by/4.0/).

manner, suggesting it associates with resection-mediated ssDNA.[11,27–29] Cdc13 can then recruit telomerase, promoting telomere addition even in the absence of telomeric sequences, albeit with less efficiency.[11,30–32]

However, whether the CST complex contributes to DSB repair in budding yeast is not known. Direct genetic approaches to address this question are hindered by the essentiality of CST's telomeric functions. In this work, we set up an experimental system with an inducible Cas9 DSB in a yeast strain with a single circular chromosome devoid of telomeres, in which the CST is no longer essential for survival. Through a comprehensive analysis of the mutation signature after DSB repair, we demonstrate that the CST complex, and prominently Stn1, contributes substantially to NHEJ-mediated repair of the DSB through its interaction with Polα-primase. More specifically, we show that CST acts after resection initiation and is critical for intermediate-size deletions (IDs; 5–85 bp) formed through NHEJ or MMEJ. Altogether, we reveal an important role of the CST complex in DSB repair as the mediator of a back-up NHEJ pathway that acts after resection.

RESULTS

Inducible Cas9 DSB in a single circular chromosome strain

To uncouple the role of the CST complex at a DSB from its telomeric functions, we generated a yeast strain devoid of telomeres (Figure 1A). We took advantage of a strain where all 16 chromosomes are fused into a single linear one (SY14)[33] to build a strain with a single circular chromosome. To do so, we induced two simultaneous Cas9 cuts at the two remaining subtelomeres and recombined them with a chimeric oligonucleotide template homologous to both sequences. The successful chromosome fusion and the absence of telomeres were confirmed by Southern blot (Figures S1A and S1B). Chromosome conformation capture analysis by Hi-C confirmed the circular nature of the single chromosome and showed no other significant structural difference between the single linear and single circular chromosomes (Figure S1C).

As also shown previously by others,[34,35] a single circular chromosome strain is viable without telomerase, confirming that telomere maintenance was no longer needed (Figure 1B). Similarly, telomere protection was no longer essential since the deletion of any of the CST genes did not impair cell survival (Figure 1B), as observed previously.[34]

To investigate DSB repair, we targeted, in the single circular chromosome strain, a unique site in the 5′ UTR of the *LYS2* gene using a plasmid expressing a specific guide RNA and a galactose-inducible Cas9 (Figure 1A). DSB induction was triggered by plating cells on galactose-containing media, and survival was quantified by spot assays and colony-forming units relative to control cells plated on glucose. Since Cas9 is continuously expressed in galactose-containing media, repair of this DSB requires a mutational event to prevent further cutting by Cas9 and allow cell survival. Following DSB induction, the survival of the wild-type (WT) circular chromosome strain (mean ± SD = 1.41% ± 0.82%) was similar to the survival of the WT linear chromosome strain (1.55% ± 0.35%) (Figures 1C and 1D), indicating that chromosome circularization did not significantly alter DNA repair efficiency. We thus used the circular chromosome strain together with this single Cas9-inducible DSB as an experimental system to investigate the specific roles of the CST complex in DSB repair.

Repair of the Cas9 DSB occurs through NHEJ and MMEJ

We then characterized the mechanisms underlying repair in our experimental system. Survival after DSB induction primarily depended on the NHEJ pathway components ligase 4 (encoded by *DNL4*) and Yku80, as expected (Figures 1C and 1D). Survival was also largely dependent on Pol4, which is required for small fill-ins around the break and contributes significantly to the inaccurate NHEJ pathway.[36–38]

In mammalian cells, repair of Cas9 DSBs has also been reported to produce large deletions (LDs) ranging from ~100 bp to several kb.[39,40] To evaluate the frequency of this outcome in our experimental system, we analyzed the colonies surviving DSB induction by multiplex PCR with one pair of primers flanking the DSB (amplicon size of 176 bp) and another one in an unrelated region of the genome to control for PCR efficiency, expecting that LDs would lead to unproductive PCRs. A PCR product around the cut site was amplified in 114 out of the 115 (99.1%) WT colonies tested (Figure S2A). Limited variations of the size of the amplicon were observed and were consistent with local insertions and deletions introduced by NHEJ or MMEJ repair. We further investigated the single unproductive PCR by PCR mapping and junction sequencing and found that, instead of an LD, it corresponded to an inversion of a 127-bp sequence close to the cut site associated with limited deletions at the boundaries (Figure S2B). Thus, no LD was found among the 115 survivor colonies tested.

We then investigated the mutational signature at the Cas9 DSB site by high-throughput sequencing of a 231-bp amplicon across the DSB site from thousands of survivors using a method developed by the Tijsterman lab.[21,41] Such an approach has proved extremely powerful at characterizing the spectrum of DSB repair outcomes and dissecting their genetic determinants.[21,38,42,43] Analysis of the repaired sequences revealed that WT cells with a circular chromosome mostly exhibited a mixture of deletions and insertions, with minor fractions of other types of mutations (deletion with insert, templated insertion, and single-nucleotide variant [SNV]), similar to the linear chromosome strain (Figures 2A and S2C). In contrast to WT, NHEJ-deficient mutants (*dnl4Δ*, *yku80Δ*, and *pol4Δ*) showed a dramatic disruption of the mutational signature, with a strong decrease in insertions and deletions in survivors, confirming repair by inaccurate NHEJ in WT (Figures 2A and S2C).

Upon closer examination of the sequences, we identified three predominant types of mutations in the WT circular strain at the repaired cut site: (1) insertion of a single base, (2) deletion of 1–4 bases, and (3) deletion of 5–85 bases (Figures 2B and 2C).

We observed high-frequency insertions of a single nucleotide adjacent to an existing identical nucleotide at the Cas9 cut site (A in 19.5% ± 9.8% and C in 7.0% ± 3.8%) (Figure 2B). As proposed by Lemos and colleagues,[38] these insertions may be caused by the occasional non-blunt cutting by Cas9, leaving a 5′ overhang of one nucleotide, which would subsequently be filled by Pol4 and then ligated. In line with this hypothesis, the *pol4Δ* mutant

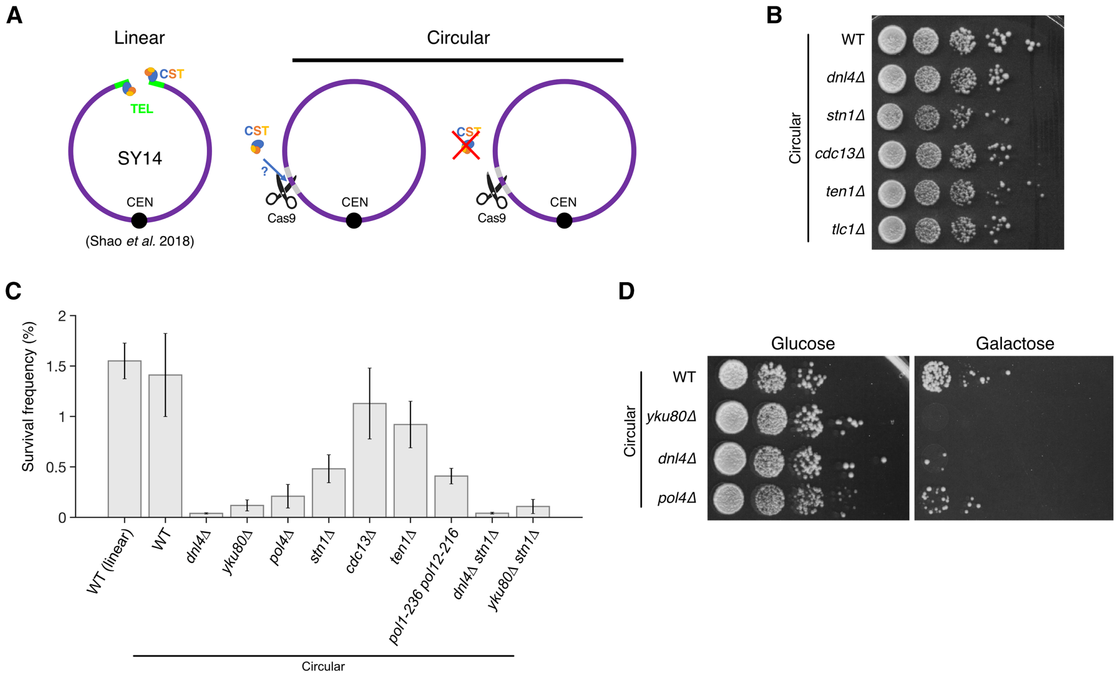

Figure 1. A yeast strain with a single circular chromosome and an inducible DSB as an experimental system

(A) Schematic representation of the yeast strain with a single linear chromosome (SY14, left)[33] and the one built in this work with a single circular chromosome and an inducible Cas9 DSB (middle and right), allowing for viable deletion of any CST subunit gene.

(B) Spot assay with the indicated strains showing cell viability on rich YPD media.

(C) Survival frequency after DSB induction for the indicated strains. Means and standard deviations are shown for n = 3–6 independent experiments.

(D) Spot assay showing cell survival and growth with (galactose-containing plate, right) or without (glucose-containing plate, left) Cas9 induction for the indicated strains.

See also Figure S1.

showed a much lower frequency of these one-base insertion events (A: 3.3% ± 4.2%; C: 0.026% ± 0.016%) (Figure 2B). These insertions were also dependent on Yku80 and Dnl4, confirming that they resulted from NHEJ repair.

In WT cells, small deletion (SD) repair events (1–4 bp) occurred near the cut site and showed few (<4 bp) or no microhomologies at their boundaries, consistent with NHEJ-associated deletions. This was confirmed by their near-complete absence in *yku80Δ* and *dnl4Δ* mutants, with one notable exception: the deletion of a G from the PAM sequence, which occurred at very low frequencies in WT cells (Figure 2C, red star). The position of this mutation, 4–5 bp away from the cut site, suggested that it was unrelated to the DSB itself and probably arose spontaneously before DSB induction in an NHEJ-independent manner. It was then selected because it impaired Cas9 cleavage and became more prominent in *yku80Δ* and *dnl4Δ* mutants due to their low survival rates. In the *pol4Δ* mutant, SDs were still detected to some extent, with variability between experiments.

IDs ranging from 5 to 85 bp were generally bidirectional and frequently removed the PAM and a substantial portion of the guide sequence, with some deletions being particularly favored (e.g., deletion of 9 bp using CAA as microhomology and deletion of 19 bp using CA as microhomology), suggesting sequence-specific preference (Figure 2C). 3.1% of all mutations corresponded to IDs exhibiting 4 or more bp of tandem microhomologies, suggestive of MMEJ repair. MMEJ usage was supported by their enrichment in the *yku80Δ* mutant (57.9%), their dependence on ligase 4, and their partial dependence on Pol4 (Figures 2C and S2C).[44–46] To more precisely test the role of Pol4 in MMEJ, we analyzed the double *yku80Δ pol4Δ* mutant and observed that the highly enriched MMEJ-associated deletions of 47–67 bp found in *yku80Δ* were completely eliminated (Figure 2C). In one of the two independent *yku80Δ pol4Δ* experiments, however, another 122-bp deletion associated with an 8-bp microhomology was detected, suggesting that Pol4 might be dispensable for some MMEJ events.

Surprisingly, the other IDs (40.2% of all mutations) found in the WT were not associated with significant microhomology (<4 bp) and required Yku80 and Dnl4, indicating that they arose from NHEJ repair (Figure 2C). A subset of these IDs, including 4 distinct 19-bp deletions, remained in the *pol4Δ* mutant but were still Yku80 dependent, as evidenced by their absence in the double *yku80Δ pol4Δ* mutant. The deletion size of 5–85 bp in IDs suggested that repair occurred after some processing of the break, most likely by short-range resection.

Overall, the Cas9 DSB led to both NHEJ- and MMEJ-dependent mutations, including insertions and both SDs (1–4 bp deletions) and IDs (5–85 bp deletions). This experimental setup allowed us to investigate CST's contribution to these repair events.

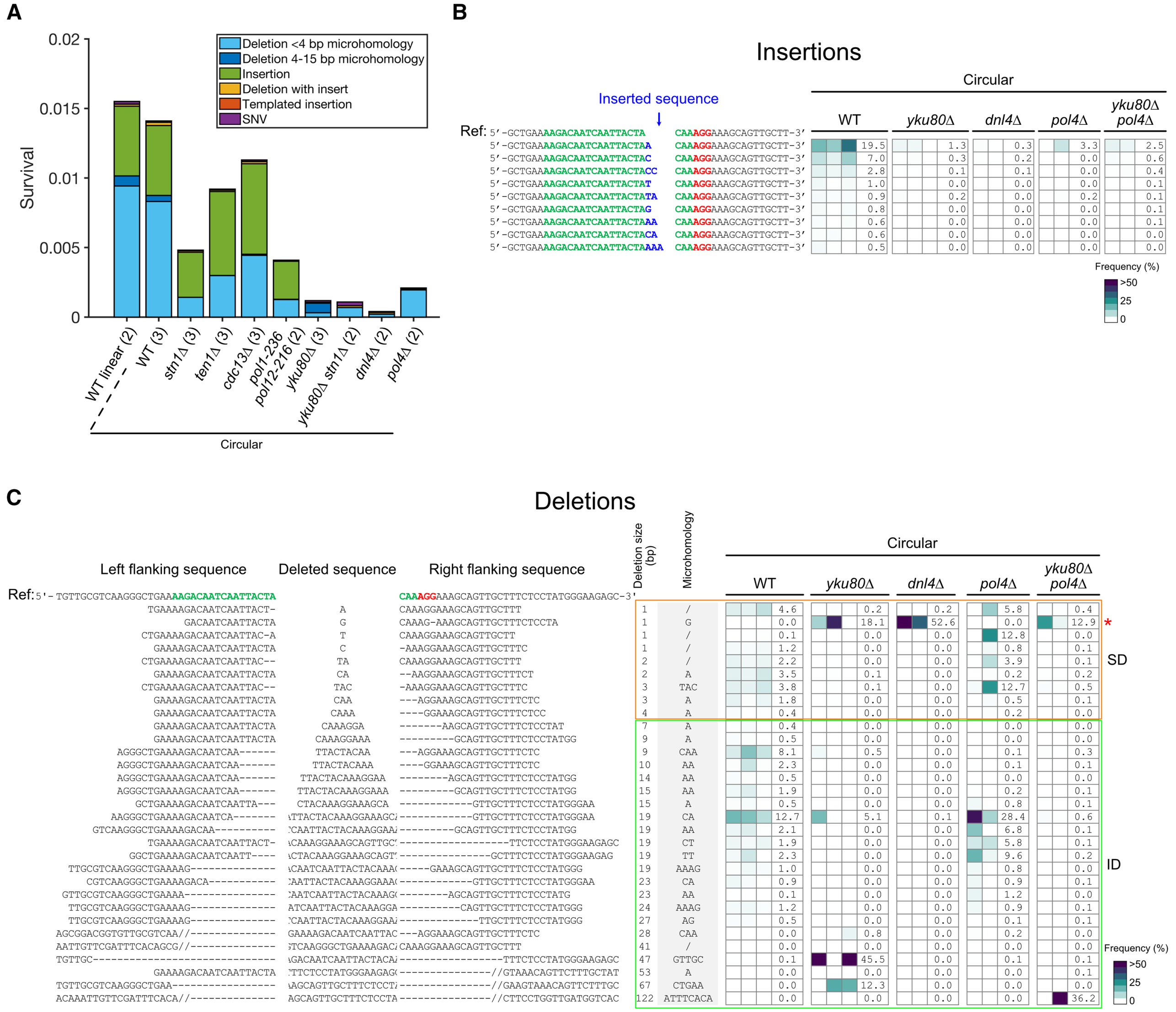

Figure 2. NHEJ and MMEJ mediate repair of the Cas9 DSB

(A) Deep sequencing analysis of mutation signature after DSB repair in the indicated strains, with fractions of different mutation types shown as stacked bars, normalized by survival frequency. Between ~200 and ~3,000 colonies surviving Cas9 DSB induction were collected in each experiment. Multiplex high-throughput Illumina sequencing of the amplicon around the DSB was performed. Analysis and clustering were done using SIQ.[21,41] Parentheses show the number of independent experiments.

(B) Heatmap of the frequency of each repair outcome with an insertion for individual experiments (in columns) with the indicated strains. For each strain, a column indicates the average frequency (in %) of each insertion. The inserted sequences are shown in blue, the sequence targeted by the guide RNA in green, and the PAM sequence in red. The insertions were ordered according to their increasing average frequency in the wild-type circular strain. Only insertions that appear with an average frequency of >0.5% are shown. The color bar shows the frequency scale. See Data S1 and S2 for the unfiltered data.

(C) Heatmap of the frequency of each repair outcome with a deletion for individual experiments (in columns) with the indicated strains. The deleted (cropped for size constraints) and flanking sequences are indicated. The deletions are ordered according to their increasing sizes. The "microhomology" box shows whether microhomologies were found at the boundaries of the deletion, with the sequence indicated. For each strain, a column indicates the average frequency (in %) of each deletion. Only deletions that appear with a frequency of >1% in at least one experiment are shown. Red asterisks: deletion of one G located 4–5 bp away from the cut site, probably not due to the repair of the DSB. The color bar shows the frequency scale. See Data S1 and S2 for the unfiltered data.

See also Figure S2.

CST contributes to inaccurate NHEJ repair

Since Cdc13 can recruit telomerase and could thus allow the propagation of a relinearized chromosome as a viable repair outcome, we first wondered whether telomere healing contributed to survival after DSB induction in our system. However, the telomerase-negative *tlc1Δ* mutant did not affect survival (Figure S3A), which, in addition to the observation that the only unproductive PCR around the DSB in WT survivors could be

assigned to an inversion (Figures S2A and S2B), indicated that telomere healing was not a significant survival pathway in this experimental setting.

To investigate the implication of the CST complex in DSB repair, we deleted each of the 3 subunit genes and found that survival after DSB induction decreased, with the *stn1Δ* mutant showing the strongest effect (Figures 1C and 3A). We found a similar result when the DSB was induced at another locus, i.e., the 5′ UTR of *URA3* (Figure S3B). Combining *STN1* deletion with *dnl4Δ* or *yku80Δ* did not further decrease survival, indicating that Stn1's contribution to repair was mostly NHEJ dependent (Figures 1C and 3A). CST is thus an important contributor to inaccurate NHEJ.

To test whether CST also affects error-free NHEJ, we performed a plasmid religation assay. Circular plasmid transformation efficiency was similar in WT, *stn1Δ*, and *dnl4Δ* (Figure 3B). However, upon linearization, transformation efficiency dropped by ~200-fold in *dnl4Δ*, as expected, but not in *stn1Δ*, indicating that Stn1 did not play a significant role in error-free NHEJ.

Thus, the CST complex does not affect error-free NHEJ but plays a telomerase-independent role in DSB repair, mostly through inaccurate NHEJ.

CST does not affect NHEJ-mediated insertions

To precisely dissect the specific NHEJ pathway in which CST plays a role, we asked whether CST affected NHEJ-mediated insertions. Examination of the mutation signature from high-throughput sequencing in CST deletion mutants revealed that the mutation signature at the repaired cut site differed from both WT and NHEJ mutants (Figures 2A and S2C). The overall frequency of insertions adjusted for survival was comparable to that of WT or slightly decreased in *stn1Δ* (Figure 2A). Detailed analysis confirmed that CST mutants did not alter the distribution of the most frequent insertions compared to WT, with single-nucleotide insertions of A or C remaining the most frequent (Figure 3C). This finding was corroborated at a different DSB locus (5′ UTR of *URA3*) where the distribution of insertions remained similar between WT and *stn1Δ* (Figure S3C). We thus conclude that CST does not influence NHEJ-mediated small insertions.

CST is specifically required for IDs

We next investigated CST's contribution to all 3 deletion size ranges we defined: LDs, IDs, and SDs. Using the multiplex PCR assay, we found that 11 out of 88 (12.5%) *stn1Δ* colonies led to unproductive PCR results (Figures 4A and S4A). Sequencing a random subset of 8 of these clones revealed LDs of 924 (*n* = 1), 7,551 (*n* = 5), 8,777 (*n* = 1) and 8,778 (*n* = 1) bp, involving microhomologies of 5, 22, 11, and 11 bp, respectively (Figure S4B), most likely resulting from MMEJ repair. In *cdc13Δ* and *ten1Δ*, we also detected more LDs compared to in the WT, with frequencies of 4 out of 50 (8%) and 2 out of 49 (4.1%), respectively, although the Fisher's exact test did not quite reach statistical significance for *ten1Δ* (p = 0.088) (Figures 4A and S4C). Thus, CST's activity limits MMEJ-mediated LDs.

In stark contrast to these LDs, the deletions captured by high-throughput sequencing were significantly decreased overall in the 3 CST mutants, although to different extents, with *stn1Δ* again showing the strongest effect (Figure 2A). However, not all deletions required the CST complex. SDs were not decreased in CST mutants, whereas IDs decreased by 5- to 10-fold in CST mutants compared to the WT (Figures 4B–4D), an observation that we confirmed at another DSB locus (Figures S4D and S4E). Interestingly, the NHEJ-dependent IDs still present in *pol4Δ* were Stn1 dependent, as shown using the *pol4Δ stn1Δ* double mutant (Figure S5), indicating that Stn1's role in promoting IDs is not Pol4 dependent. Among IDs, those with larger microhomologies (4–15 bp) were drastically reduced in CST mutants compared to the WT, suggesting decreased MMEJ repair (Figures 2A and S2C). We took advantage of the observation that some microhomology-associated IDs of 47–67 bp were enriched in *yku80Δ* (Figures 2C and S2C) to further test the role of CST in their formation. We generated the double mutants *yku80Δ stn1Δ*, *yku80Δ cdc13Δ*, and *yku80Δ ten1Δ* and found a strong decrease of Yku80-independent microhomology-associated IDs from 57.9% to 0%–6.7% in all 3 double mutants (Figures S2C and S5).

Altogether, these data indicate that CST's activity leads to distinct outcomes depending on the size of the deletions: no effect on SDs, stimulation of IDs, and inhibition of LDs. Since CST promotes IDs regardless of the mechanism (NHEJ or MMEJ) used to complete repair, one hypothesis would be that CST acts upstream of repair per se, most likely by limiting the formation of ssDNA, the extent of which would define the deletion size and thus the formation of IDs or LDs. Considering CST's interaction with Polα-primase and its role in post-replication fill-in synthesis at resected telomeres, we wondered whether CST functions after DSB resection to recruit Polα-primase and regulate ssDNA.

The CST complex limits resection by recruiting Polα-primase for fill-in synthesis

To test the relationship between CST and resection, we first asked whether limiting ssDNA formation could compensate for *stn1Δ*'s survival defect after DSBs. We thus mutated the first step of resection dependent on the Mre11/Rad50/Xrs2 (MRX) complex and Sae2, either by deleting *SAE2* or by generating the nuclease-dead mutant *mre11-H125N*. As previously observed, survival by NHEJ repair significantly increased in these mutants (Figure 5A).[44,47] Deletion of *STN1* in these mutants did not decrease survival (Figure 5A), indicating that Stn1 is not required when resection is already limited. These results also suggest that CST acts downstream of resection in DSB repair.

To determine whether the recruitment of Polα-primase by CST would affect the extent of ssDNA, we introduced point mutations in *POL1* (*pol1-236*: D236N) and *POL12* (*pol12-216*: G325D), which specifically disrupt the interaction between Polα and CST,[12,14,49,50] and directly measured ssDNA accumulation 1 kb from the cut site by a restriction digest/qPCR method.[48] As a positive control, the *yku80Δ* mutant showed more ssDNA than WT after DSB induction, consistent with increased resection (Figure 5B).[51,52] In *stn1Δ* and *pol1-236 pol12-216*, this assay also measured higher levels of ssDNA compared to the WT (Figure 5B). Furthermore, using a SSA reporter, in which 82 bp of homology have been inserted at ~10 kb on both sides of the

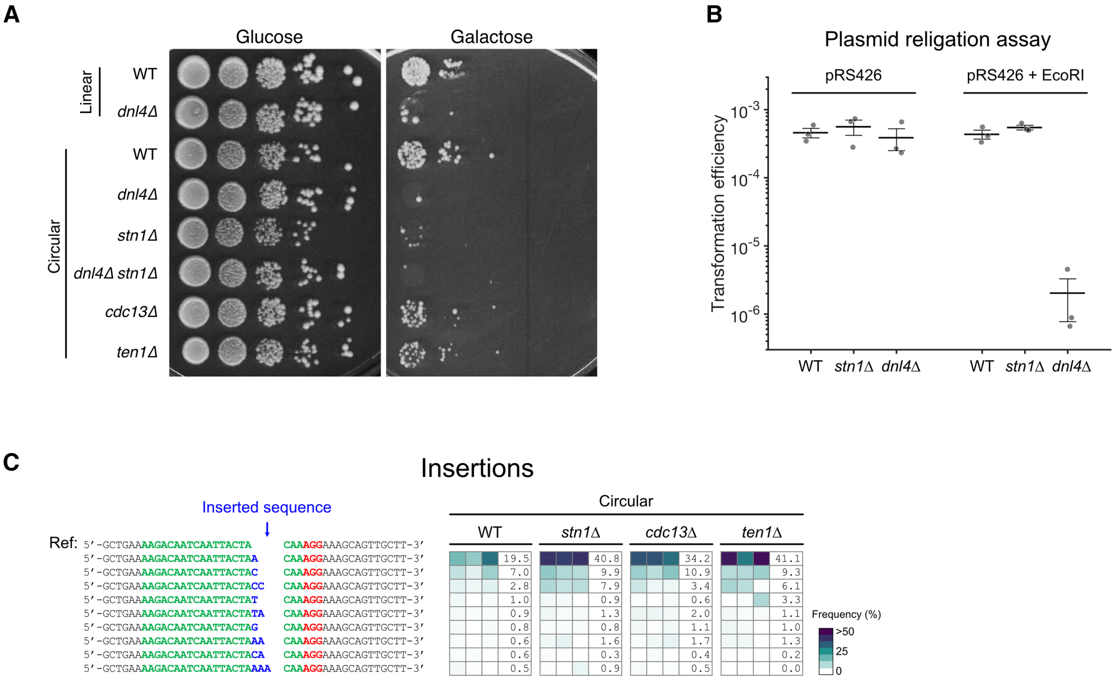

Figure 3. CST contributes to inaccurate repair of the DSB

(A) Spot assay as in Figure 1D with the indicated strains, including CST deletion mutants.

(B) Plasmid religation assay. Transformation efficiency corresponds to the number of colonies formed on the selective plate without uracil normalized by the number of plated cells, as assessed on a non-selective YPD plate. Each dot represents an independent transformation experiment. Error bars show the standard error of the mean, and the middle bar represents the mean. The strains were transformed either with the circular plasmid pRS426 or with the same plasmid linearized by EcoRI digestion ("pRS426 + EcoRI").

(C) Heatmap of the frequency of each repair outcome with an insertion for individual experiments (in columns) with the indicated strains as in Figure 2B. For each strain, a column indicates the average frequency (in %) of each insertion. The data for the WT strain are reused from Figure 2B. See Data S1 and S2 for the unfiltered data.

See also Figure S3.

DSB and would allow the reconstitution of a functional *LEU2* gene after repair, we observed ∼5-fold increased survival on plates lacking leucine in the *stn1Δ* strain and a more modest but statistically significant ∼40% increase in the *pol1-236 pol12-216* mutant, consistent with enhanced resection (Figures 5C and 5D).

These results indicate that the CST complex acts after resection initiation mediated by the MRX/Sae2 complex to subsequently limit the extent of ssDNA by fill-in synthesis through its interaction with Polα-primase.

CST's interaction with Polα-primase controls the balance between IDs and LDs

Because of its effect on ssDNA at DSBs, we predicted that CST's interaction with Polα-primase was important for repair pathway choice. First, survival after DSB induction was decreased in the *pol1-236 pol12-216* mutant to an extent similar to *stn1Δ*, and no additive effect was observed when combined with *stn1Δ*, suggesting that CST and Polα act together in repair (Figures 1C and 6A).

We next tested whether CST's opposite effects on IDs and LDs involved Polα-primase. As observed for CST mutants, the multiplex PCR assay followed by junction mapping revealed that in 4 out of 87 (4.6%) *pol1-236 pol12-216* survivor colonies, unproductive PCRs were due to LDs of 7,551 bp mediated by a 22-bp microhomology (Figures 4A and S6).

Using high-throughput sequencing of the repaired cut site in survivors, we found that the mutation signature of the *pol1-236 pol12-216* mutant closely resembled that of *stn1Δ* and the CST mutants in general (Figures 6B and 6C). More specifically, the distributions of NHEJ-mediated insertions and SDs were not altered in *pol1-236 pol12-216* compared to the WT and *stn1Δ* (Figures 6B–6D). In contrast, both NHEJ- and MMEJ-mediated IDs were strongly reduced compared to the WT and similar to *stn1Δ* (Figures 6C, 6E, 2A, and S2C).

To further test the generality of these observations, we used strains with linear genomes (i.e., SY14 with a single chromosome and the parental BY4742 with 16 chromosomes), in which *pol1-236 pol12-216* mutants are viable. The same mutational signature as in the WT with the circular chromosome was found in SY14 and BY4742, indicating that DSB repair was not altered in the single circular chromosome strain compared to linear genomes with telomeres (Figures 6B and 6C). In both SY14 and BY4742 backgrounds, upon introducing the *pol1-236 pol12-216* mutation, no major change in the insertions and SDs was observed compared to the WT (Figures 6B–6D). Instead, the

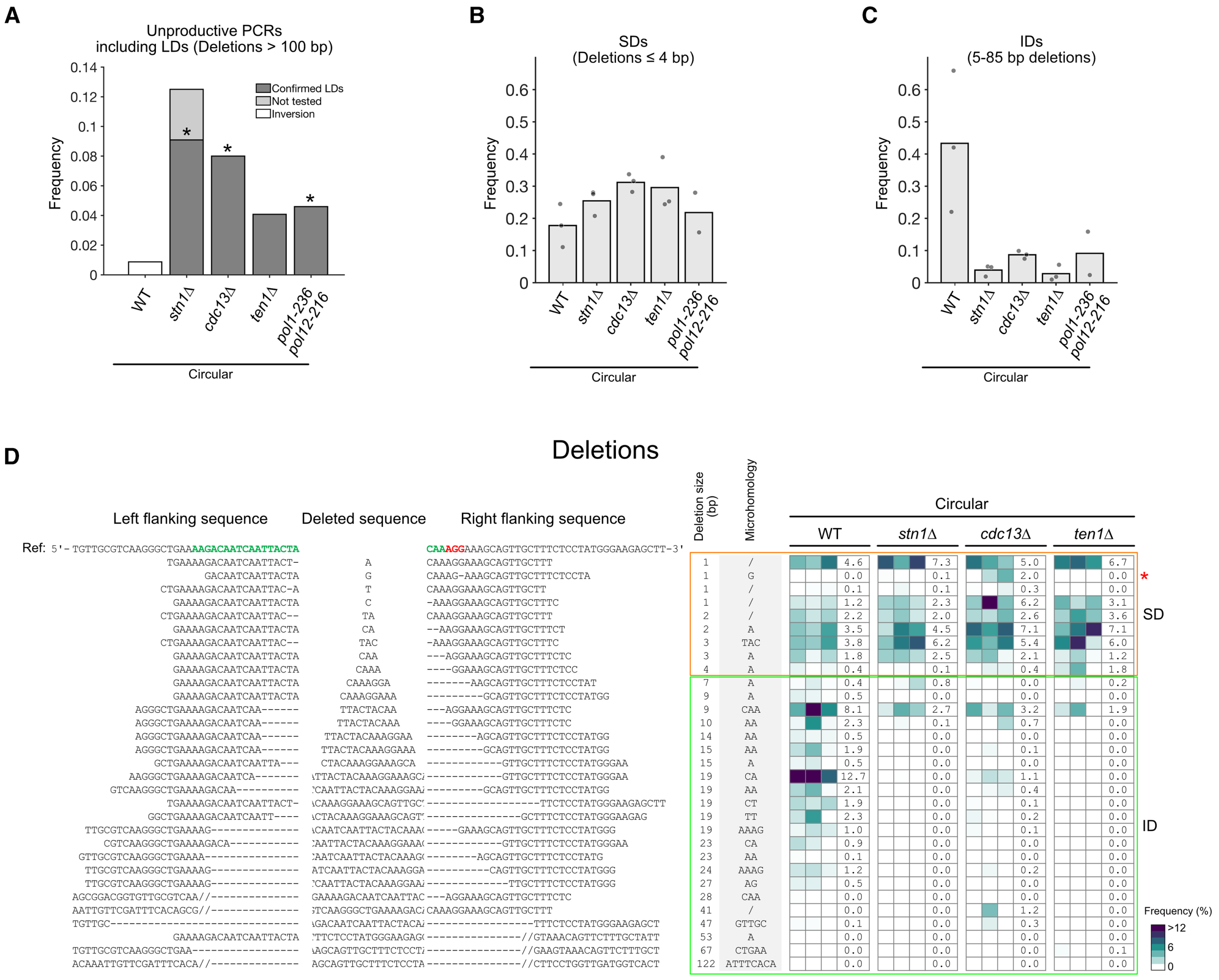

Figure 4. CST specifically affects the balance of intermediate- and large-size deletions

(A) Frequency of unproductive PCRs around the cut site in the indicated strains. PCR mapping and sequencing of the junction for the tested colonies revealed an inversion in the WT (see Figure S2B) and LDs in *stn1Δ*, *cdc13Δ*, *ten1Δ*, and *pol1-236 pol12-216* (see Figures S2A, S4A, S4C, and S6). **p* < 0.05 compared to WT using two-tailed Fisher's exact test on LD frequencies (*n* = 115, 88, 50, 49, and 87 for WT, *stn1Δ*, *cdc13Δ*, *ten1Δ*, and *pol1-236 pol12-216*, respectively).

(B) Frequency of SDs, i.e., deletions ≤ 4 bp, for the indicated strains. Each dot represents an independent experiment.

(C) Frequency of IDs, i.e., 5–85 bp deletions, for the indicated strains.

(D) Heatmap of the frequency of each repair outcome with a deletion for individual experiments (in columns) with the indicated strains, represented as in Figure 2C except for the color bar, which uses a different scaling. For each strain, a column indicates the average frequency (in %) of each deletion. The data for the WT strain are reused from Figure 2C. See Data S1 and S2 for the unfiltered data.

See also Figures S4–S6.

mutation signatures were characterized by a marked decrease of IDs (Figures 6C and 6E), mimicking the effects of *pol1-236 pol12-216* and *stn1Δ* mutations in the circular strain.

To evaluate if our conclusions are relevant beyond Cas9-generated DSBs, we also introduced the *pol1-236 pol12-216* mutant in a widely studied strain (JKM179) in which the HO endonuclease is under the control of a galactose-inducible promoter and the HML/HMR loci are deleted to prevent HR. In the WT strain, the mutation signature was dominated by insertions of CA (18%) and ACA (5.4%) and by deletions of ACA (37%) and A (7.0%) (Figures S7A–S7C), consistent with previous studies.[43,53,54] Interestingly, we were able to detect rare insertions of 50–300 bp sequences captured from mitochondrial DNA and Ty1 retrotransposons (Data S1), as reported before.[55,56] We found that, similarly to the repair of the Cas9 DSB, while all insertions were Pol4 dependent, a subset of deletions were still present in the *pol4Δ* mutant, including the deletions of ACA, GCA, and GC (Figures S7B–S7E). Previous works also noted the presence of IDs (>4 bp), which we detected at a frequency of 11% in our analysis (Figures S7C and S7E). Remarkably, the frequency of IDs was decreased 4-fold in the *pol1-236 pol12-216* mutant (2.8%), whereas the SDs and

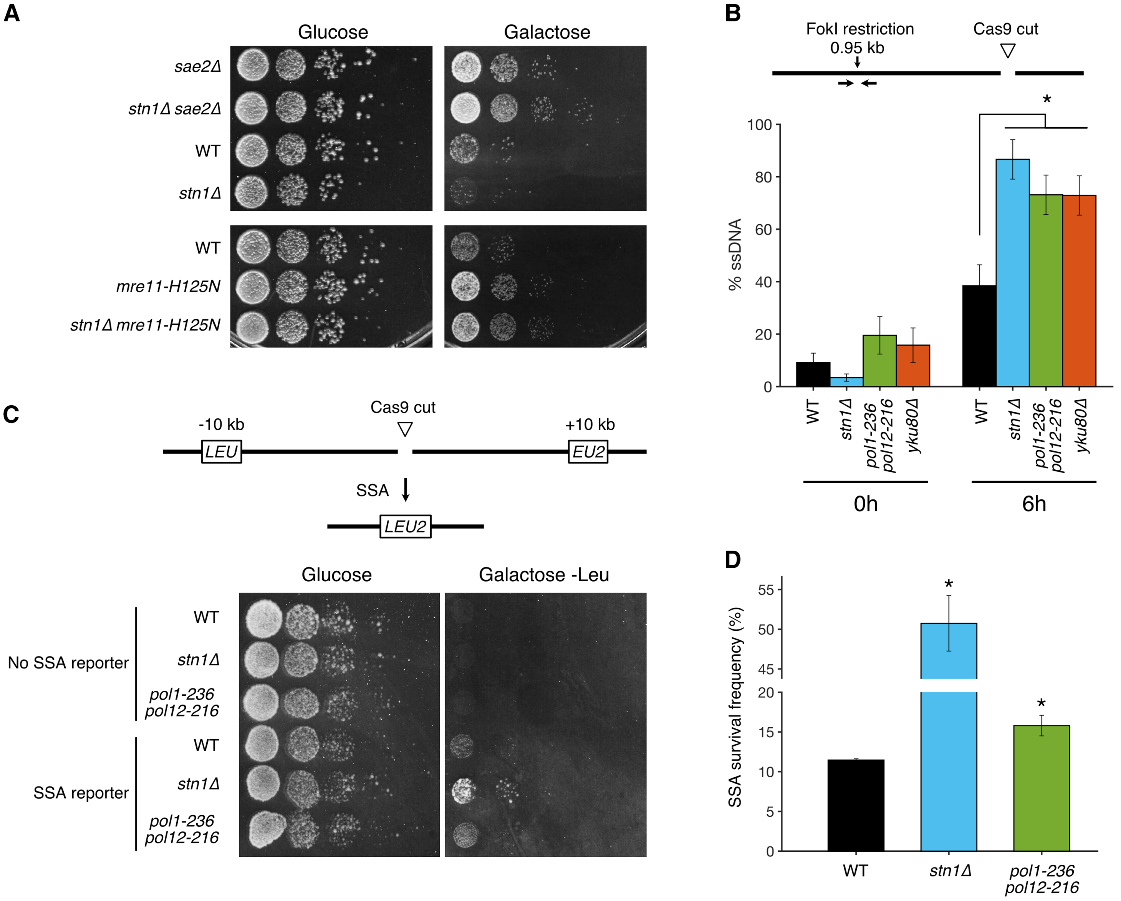

Figure 5. The CST complex limits resection through its interaction with Polα-primase

(A) Spot assay with the indicated strains showing cell survival and growth with (galactose-containing plate, right) or without (glucose-containing plate, left) Cas9 induction.

(B) Quantification of ssDNA in the indicated strains ($n = 5$ independent experiments for WT and $n = 3$ for the others) at 0 and 6 h after DSB induction by qPCR after FokI digestion of the locus 0.95 kb away from the DSB, following the method described in Zierhut and Diffley[48] and using within time-point normalization. The bar plots show the mean, and error bars represent the standard deviation. $^*p < 0.05$ compared to WT using a two-tailed Student's t test.

(C) SSA assay using reconstitution of *LEU2* as a readout by plating on media without leucine ("-Leu"), with (right) or without (left) Cas9 induction.

(D) Quantification of SSA survival frequency ($n = 3$ independent experiments). The bar plots show the mean, and error bars represent the standard deviation. $^*p < 0.05$ compared to WT using a two-tailed Student's t test.

insertions were preserved (Figures S7C–S7E), demonstrating that the effect mediated by CST's interaction with Polα-primase is not restricted to blunt Cas9-induced DSBs.

Altogether, our findings indicate that CST and Polα-primase act together in DSB repair to promote both NHEJ- and MMEJ-mediated IDs. In the absence of CST or when its interaction with Polα is disrupted, these IDs can no longer be formed, and repair is either impossible, provoking cell death, or will instead be associated with LDs.

Rev7 and Rif1 also participate in ID formation

In metazoans, CST functions in the 53BP1-RIF1-Shieldin pathway to promote NHEJ. While no budding yeast orthologs of SHLD1, SHLD2, and SHLD3 have been found, REV7, a subunit of Polζ involved in translesion synthesis but also a component of the Shieldin complex, is conserved in yeast.[57] Rif1, the ortholog of RIF1 in yeast, was initially identified as a Rap1-binding factor regulating telomere length.[58] To test whether a similar pathway is conserved in budding yeast despite the absence of the Shieldin complex, we analyzed the mutation signature after Cas9 DSB repair in *rev7Δ* and *rif1Δ* mutants. Similar to CST mutants, the overall mutation landscape of *rev7Δ* and *rif1Δ* mutants showed a global decrease in deletions, including the ones associated with microhomologies of >4 bp, and, conversely, a relative increase in small insertions (Figures S8A–S8C). Closer analysis of deletions showed that SDs were maintained in *rif1Δ*, and interestingly, they were enriched ∼2.5-fold in *rev7Δ* compared to the WT (Figures S8C and S8D). We found a specific decrease of IDs in *rev7Δ* and *rif1Δ* mutants to an extent similar to *stn1Δ* (Figures S8C and S8E). The overall similarity between CST/Polα-primase mutants and *rev7Δ/rif1Δ* suggests that the 53BP1-RIF1-Shieldin pathway might be partially conserved despite the absence of some factors in yeast.

CellPress
OPEN ACCESS

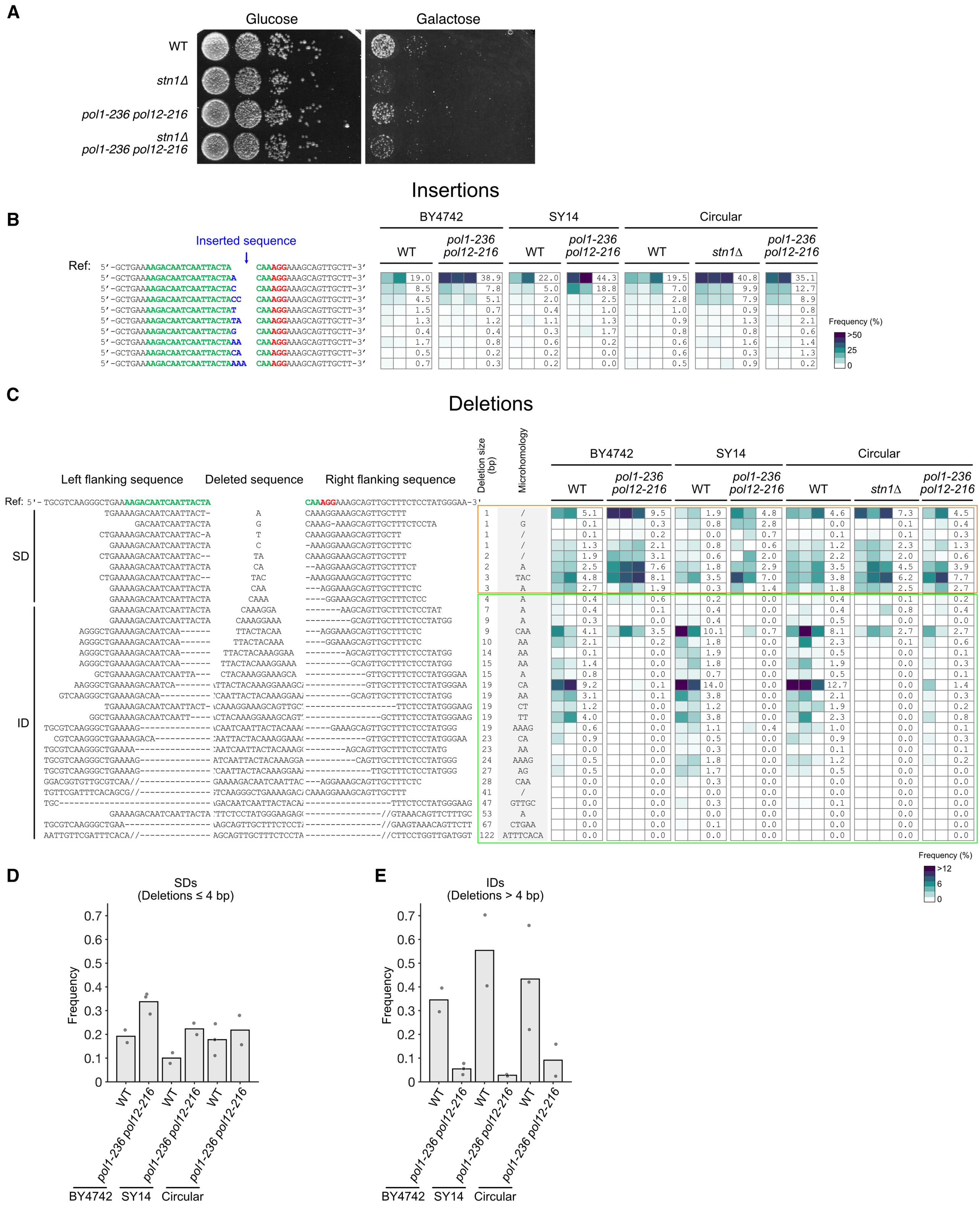

Figure 6. CST's interaction with Polα-primase is critical for IDs

(A) Spot assay with the indicated strains showing cell survival and growth with (galactose-containing plate, right) or without (glucose-containing plate, left) Cas9 induction.

(legend continued on next page)

DISCUSSION

In this work, we report that the CST complex and Polα-primase participate in DSB repair by NHEJ and MMEJ by counteracting resection. Collectively, our findings lead to the following mechanistic model (Figure 7). Once the DSB is formed, recruitment of the MRX and Ku complexes allows the ligation of the two ends through NHEJ, leading to error-free repair. Additional processing of the DSB, in particular if the DSB is not blunt or has other, more complex structures, followed by ligation, creates limited local mutations, such as small insertions and SDs, or SNVs. In all these cases, the CST appears not to be involved, as no extensive ssDNA has been exposed yet. However, the MRX-Sae2 complex also initiates resection, which can kinetically compete with NHEJ. The CST complex, together with Polα-primase, can then be recruited to the ssDNA, which would initiate fill-in synthesis, limit the extent of ssDNA, and, importantly, create a stable structure composed of dsDNA and hybrid RNA-DNA close to the cut site. This structure can then be amenable to NHEJ repair and lead to IDs (~5–85 bp). We propose that the size of the deletion is determined by the position of the CST complex on the resected DNA with respect to the DSB site and where Polα-primase initiates RNA primer synthesis. Rev7 and Rif1 are also involved in the formation of IDs, and consistent with this, a recent study has suggested that Rev7 promotes NHEJ by interacting with MRX.[59] How Rev7 and Rif1 are coordinated with the CST complex in DSB repair will need further investigation to better assess the potentially partial conservation of a 53BP1-RIF1-Shieldin-CST- Polα-primase pathway from metazoans to yeast.

When the CST complex is absent, no double-stranded structure is formed near the break site, and resection is not counteracted. NHEJ repair is then no longer available as a repair outcome. Nonetheless, homologies exposed during resection can mediate MMEJ- or SSA-dependent LDs (0.1–20 kb). We assume that other homology-dependent repair mechanisms, e.g., HR or break-induced replication, could also be used depending on the sequence context and the presence of homology elsewhere in the genome. In the experimental setting we use, where HR is not an available outcome, we also evidence a decrease in cell survival, most prominently in *stn1Δ* and *pol1-236 pol12-216*, indicating that extensive resection is not always salvaged and becomes toxic. Interestingly, *stn1Δ* displayed a stronger impact than *cdc13Δ* on survival (Figure 1C). We propose that even in the absence of Cdc13, Stn1 and Ten1 can be recruited to the DSB through their oligonucleotide/oligosaccharide-binding (OB) folds or by an alternative mechanism, albeit with lesser efficiency, which is reminiscent of the ability of Stn1 and Ten1 to associate with and protect telomeres in a Cdc13-independent manner.[60,61]

At the genome level, while the CST complex promotes local IDs, it also prevents larger-scale deletions, thereby limiting genome instability overall. Interestingly, in mouse cells, Cas9-based genome editing frequently leads to unexpected LDs of hundreds of bp to several kb, associated with microhomologies.[39,40] It would be of great interest to investigate whether, in mammalian cells, the CST-dependent pathway modulates the rate of such unintended outcomes to optimize genome editing approaches.

CST's role in DSB repair in linear chromosome strains

We reached our conclusions using a unique experimental system designed to uncouple CST's roles at DSBs from its telomere-specific functions, i.e., without the confounding effects of telomere deprotection and without affecting cell survival. That being said, we were able to generalize our results to the context of linear chromosomes by taking advantage of the interaction mutant *pol1-236 pol12-216*, which is viable in strains with linear chromosomes (BY4742 and SY14). Indeed, the mutational signature obtained in this mutant after DSB induction in the two linear chromosome strains and in the circular chromosome strain was indistinguishable from that of *stn1Δ*. Importantly, these results suggest that the CST complex is able to act at a DSB even in the presence of telomeres, excluding that titration at telomeres would prevent CST from acting at a DSB. We were also able to extend our conclusions to the widely used HO cut, which generates a 4-nucleotide overhang instead of the mostly blunt Cas9 cut, and thus found that the IDs resulting from the repair of the HO cut also required CST/Polα-primase interaction. We thus conclude that the function of the CST complex uncovered here is likely to be verified for any DSB structure and should also be relevant in the more physiological context of a strain with linear chromosomes.

CST-dependent post-resection NHEJ leads to IDs

By leveraging an approach that enables the determination at high resolution of mutation signatures of DSB repair in several genetic contexts,[21,42] our results evidence IDs generated through NHEJ, which was unexpected since they would require resection initiation. Indeed, binding of Ku to DNA ends protects from resection, and conversely, resection displaces Ku,[3] leading to the view that resection is a commitment step into homology-dependent mechanisms and away from NHEJ. Here, we show that even after resection, NHEJ can still be used for repair in a CST- and Polα-primase-dependent manner and generate ~5–85 bp deletions. The size of the deletions suggests that they require MRX-Sae2-mediated short-range resection, which was recently mapped at base resolution at discrete positions <119 bp away from the DSB.[62] Consistently, in the nuclease-dead *mre11-H125N* and in *sae2Δ*

(B) Heatmap of the frequency of each repair outcome with an insertion for individual experiments (in columns) with the indicated strains, as in Figure 2B. Strains include the parental BY4742 with 16 chromosomes, SY14 with a single linear chromosome, and the strain with a single circular chromosome, either WT or bearing the *pol1-236 pol12-216* mutation. For each strain, a column indicates the average frequency (in %) of each insertion. The data for the circular chromosome strain (WT and *stn1Δ*) are the same as in Figure 3C. See Data S1 and S2 for the unfiltered data.

(C) Heatmap of the frequency of each repair outcome with a deletion for individual experiments with the indicated strains, as in Figure 2C. For each strain, a column indicates the average frequency (in %) of each deletion. The data for the circular chromosome strain (WT and *stn1Δ*) are the same as in Figure 4D. See Data S1 and S2 for the unfiltered data.

(D) Frequency of SDs for the indicated strains. Each dot represents an independent experiment.

(E) Frequency of IDs for the indicated strains.

See also Figures S7 and S8.

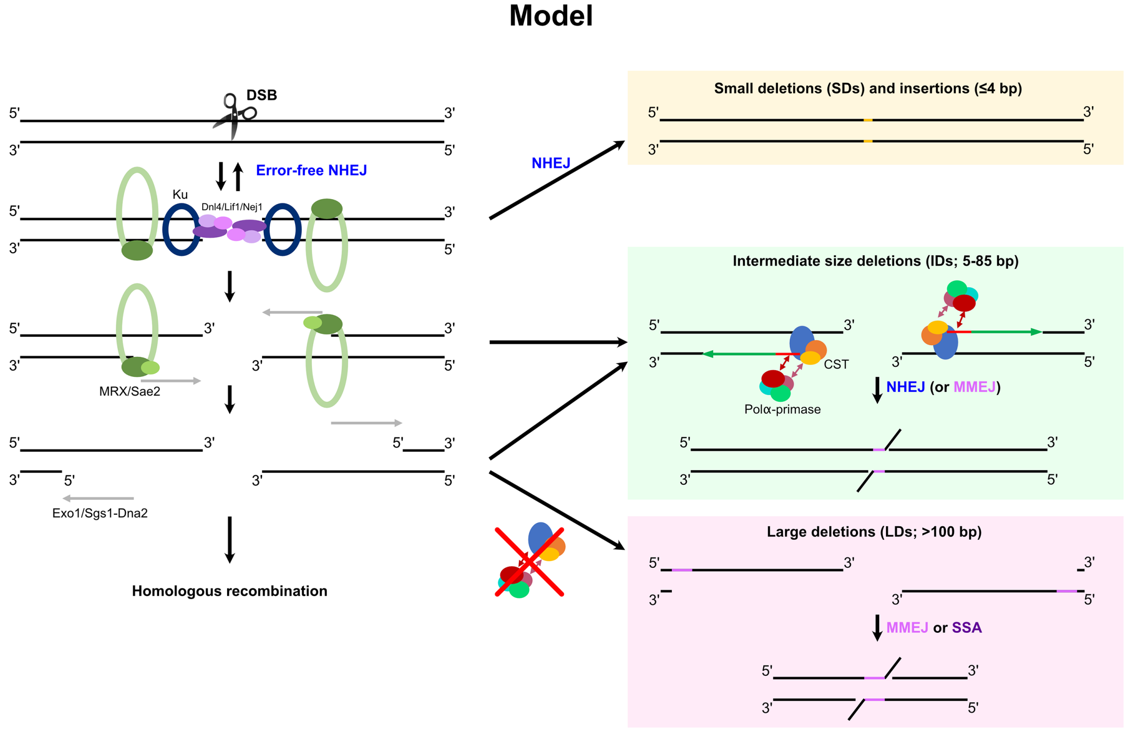

Figure 7. Mechanistic model of CST's function in DSB repair

The left side shows the canonical repair of a DSB by error-free NHEJ and HR, regulated by the initiation of resection. The right side defines the major mutational events found in this work: (1) before resection initiation, NHEJ can lead to the formation of SDs, small insertions, and SNVs; (2) after resection initiation, the CST complex recruits Polα-primase for fill-in synthesis in a back-up NHEJ pathway or for MMEJ repair, thus leading to IDs; and (3) in the absence of CST or if the interaction between CST and Polα-primase is impaired, extensive resection promotes MMEJ- or SSA-mediated LDs and other rearrangements.

mutants, *STN1* deletion no longer affects survival after DSBs. In line with these results, the recruitment of Cdc13 to a DSB was previously found to be Mre11 dependent.[28]

Mechanistically, CST and Polα-primase counteract resection by limiting the extent of ssDNA. We propose that Polα-primase, recruited by CST, creates a double-stranded hybrid structure close to the DSB and facilitates NHEJ through two possible ways. First, the Ku complex requires double-stranded ends, which can include such RNA-DNA hybrids, but it is also versatile enough to accommodate some 3′ ended ssDNA.[63] After its initial eviction by the resection machinery, the Ku complex can thus reload onto the structure formed by Polα-primase activity. Second, this structure might delay the extensive degradation of the 3′ ssDNA, and the enhanced stability would promote NHEJ.[48,64] We also found that NHEJ would often use the base pairing of 2–4 bp,[65,66] thus facilitating synapsis. Finally, the resolution of the local structure would require degradation of the 3′ flap, degradation of the RNA primer, and fill-in synthesis of the resulting gap and other remaining ssDNA stretches. Future investigations will be aimed at characterizing these downstream steps to obtain a full picture of how these deletions are formed.

Limitations of the study

While we provide a clear mutation signature associated with CST and Polα-primase, a molecular characterization of the activity of these factors at the DSB would strengthen our model. For instance, although Cdc13 was previously detected by chromatin immunoprecipitation at the HO cut,[28] experimentally localizing CST and Polα-primase at the Cas9 and HO cut would indicate their direct physical involvement in repair. Such an approach would also help investigate the stronger effect of *STN1* deletion as compared to the other subunits of the complex, which suggests that Stn1 could act at the DSB in a Cdc13-independent manner. Future investigation will thus aim to provide a better understanding of how CST is recruited to the DSB and how its activity is coordinated with other processing factors.

RESOURCE AVAILABILITY

Lead contact

Requests for further information and resources should be directed to the lead contact, Zhou Xu (zhou.xu@sorbonne-universite.fr).

Materials availability

All strains and plasmids generated in this study are available from the lead contact upon request.

Data and code availability

- The high-throughput sequencing raw data for the mutation signature analyses and Hi-C maps are publicly available as of the date of publication at the European Nucleotide Archive under project accession number ENA: PRJEB88514. In addition to the raw sequencing data, all processed sequencing data are published as Data S1 and S2.
- This paper does not report original code.

ACKNOWLEDGMENTS

We are grateful to Jin-Qiu Zhou for providing the SY13, SY14, and BY4742 strains. We thank Teresa Teixeira for discussions, material, and reagents and Jim Haber for plasmids and strains. We thank Stéphane Marcand for his comments on the manuscript. This work benefited from equipment and services from the iGenSeq core facility at Institut du Cerveau (ICM), supervised by Yannick Marie. We thank the Biomics core sequencing facility of the Institut Pasteur. Research in Z.X.'s lab was supported by Ville de Paris (Program Émergence[s]), the Emergence grant of Sorbonne Université, Ligue Contre le Cancer (Subvention Recherche Scientifique 2022), and Fondation ARC pour la Recherche sur le Cancer (ARCPJA202160003865 and ARCPGA2023110007341_7967). Research in K.D.'s lab was funded by Fondation ARC pour la Recherche sur le Cancer (ARCPJA2022070005353), Université Paris Cité IdEx ANR-18-IDEX-0001, and EDF. This work was also supported by the European Research Council (ERC) under the Horizon 2020 Program (ERC grant agreement 771813) and Agence Nationale pour la Recherche (ANR-22-CE12-0013-01) to R.K.

AUTHOR CONTRIBUTIONS

Investigation, O.I., L.D., C.B., L.M., and Z.X.; formal analysis, O.I., L.D., L.M., R.K., and Z.X.; conceptualization, K.D. and Z.X.; supervision, R.K., K.D., and Z.X.; resources, R.K., K.D., and Z.X.; writing – original draft, O.I., K.D., and Z.X.; writing – review & editing, all authors.

DECLARATION OF INTERESTS

The authors declare no competing interests.

STAR★METHODS

Detailed methods are provided in the online version of this paper and include the following:

- KEY RESOURCES TABLE
- EXPERIMENTAL MODEL AND STUDY PARTICIPANT DETAILS
  - Yeast strains and plasmids
  - Circular chromosome strain
- METHOD DETAILS
  - Spot assay and survival assay
  - Resection assays by quantitative PCR
  - Plasmid religation assay
  - Southern blot
  - Mutation signature analysis
  - Hi-C procedure and sequencing
  - Hi-C processing
- QUANTIFICATION AND STATISTICAL ANALYSIS

SUPPLEMENTAL INFORMATION

Supplemental information can be found online at https://doi.org/10.1016/j.xgen.2025.100947.

Received: January 2, 2025
Revised: May 2, 2025
Accepted: June 16, 2025
Published: July 16, 2025

REFERENCES

1. Harrison, J.C., and Haber, J.E. (2006). Surviving the Breakup: The DNA Damage Checkpoint. Annu. Rev. Genet. *40*, 209–235.
2. Finn, K., Lowndes, N.F., and Grenon, M. (2012). Eukaryotic DNA damage checkpoint activation in response to double-strand breaks. Cell. Mol. Life Sci. *69*, 1447–1473. https://doi.org/10.1007/s00018-011-0875-3.
3. Symington, L.S., and Gautier, J. (2011). Double-strand break end resection and repair pathway choice. Annu. Rev. Genet. *45*, 247–271. https://doi.org/10.1146/annurev-genet-110410-132435.
4. Wellinger, R.J., and Zakian, V.A. (2012). Everything you ever wanted to know about Saccharomyces cerevisiae telomeres: beginning to end. Genetics *191*, 1073–1105. https://doi.org/10.1534/genetics.111.137851.
5. Churikov, D., Corda, Y., Luciano, P., and Géli, V. (2013). Cdc13 at a crossroads of telomerase action. Front. Oncol. *3*, 39. https://doi.org/10.3389/fonc.2013.00039.
6. Garvik, B., Carson, M., and Hartwell, L. (1995). Single-stranded DNA arising at telomeres in cdc13 mutants may constitute a specific signal for the RAD9 checkpoint. Mol. Cell Biol. *15*, 6128–6138.
7. Nugent, C.I., Hughes, T.R., Lue, N.F., and Lundblad, V. (1996). Cdc13p: a single-strand telomeric DNA-binding protein with a dual role in yeast telomere maintenance. Science *274*, 249–252.
8. Grandin, N., Reed, S.I., and Charbonneau, M. (1997). Stn1, a New Saccharomyces Cerevisiae Protein, Is Implicated in Telomere Size Regulation in Association With Cdc13. Genes Dev. *11*, 512–527.
9. Grandin, N., Damon, C., and Charbonneau, M. (2001). Ten1 functions in telomere end protection and length regulation in association with Stn1 and Cdc13. EMBO J. *20*, 1173–1183.
10. Pennock, E., Buckley, K., and Lundblad, V. (2001). Cdc13 delivers separate complexes to the telomere for end protection and replication. Cell *104*, 387–396.
11. Bianchi, A., Negrini, S., and Shore, D. (2004). Delivery of yeast telomerase to a DNA break depends on the recruitment functions of Cdc13 and Est1. Mol. Cell *16*, 139–146.
12. Qi, H., and Zakian, V.A. (2000). The Saccharomyces telomere-binding protein Cdc13p interacts with both the catalytic subunit of DNA polymerase alpha and the telomerase-associated est1 protein. Genes Dev. *14*, 1777–1788.
13. Gao, H., Cervantes, R.B., Mandell, E.K., Otero, J.H., and Lundblad, V. (2007). RPA-like proteins mediate yeast telomere function. Nat. Struct. Mol. Biol. *14*, 208–214.
14. Grossi, S., Puglisi, A., Dmitriev, P.V., Lopes, M., and Shore, D. (2004). Pol12, the B subunit of DNA polymerase alpha, functions in both telomere capping and length regulation. Genes Dev. *18*, 992–1006.
15. Stewart, J.A., Wang, Y., Ackerson, S.M., and Schuck, P.L. (2018). Emerging roles of CST in maintaining genome stability and human disease. Front. Biosci. *23*, 1564–1586. https://doi.org/10.2741/4661.
16. Chastain, M., Zhou, Q., Shiva, O., Fadri-Moskwik, M., Whitmore, L., Jia, P., Dai, X., Huang, C., Ye, P., and Chai, W. (2016). Human CST Facilitates Genome-wide RAD51 Recruitment to GC-Rich Repetitive Sequences in Response to Replication Stress. Cell Rep. *16*, 2048. https://doi.org/10.1016/j.celrep.2016.08.008.
17. Lyu, X., Lei, K.H., Biak Sang, P., Shiva, O., Chastain, M., Chi, P., and Chai, W. (2021). Human CST complex protects stalled replication forks by directly blocking MRE11 degradation of nascent-strand DNA. EMBO J. *40*, e103654. https://doi.org/10.15252/embj.2019103654.
18. Jaiswal, R.K., Lei, K.H., Chastain, M., Wang, Y., Shiva, O., Li, S., You, Z., Chi, P., and Chai, W. (2023). CaMKK2 and CHK1 phosphorylate human STN1 in response to replication stress to protect stalled forks from aberrant resection. Nat. Commun. *14*, 7882. https://doi.org/10.1038/s41467-023-43685-2.
19. Mirman, Z., Lottersberger, F., Takai, H., Kibe, T., Gong, Y., Takai, K., Bianchi, A., Zimmermann, M., Durocher, D., and de Lange, T. (2018). 53BP1-RIF1-shieldin counteracts DSB resection through CST- and Polalpha-dependent fill-in. Nature *560*, 112–116. https://doi.org/10.1038/s41586-018-0324-7.
20. Barazas, M., Annunziato, S., Pettitt, S.J., de Krijger, I., Ghezraoui, H., Roobol, S.J., Lutz, C., Frankum, J., Song, F.F., Brough, R., et al. (2018). The CST Complex Mediates End Protection at Double-Strand Breaks and

CellPress
OPEN ACCESS

Promotes PARP Inhibitor Sensitivity in BRCA1-Deficient Cells. Cell Rep. *23*, 2107–2118. https://doi.org/10.1016/j.celrep.2018.04.046.

21. Schimmel, J., Muñoz-Subirana, N., Kool, H., van Schendel, R., and Tijsterman, M. (2021). Small tandem DNA duplications result from CST-guided Pol alpha-primase action at DNA break termini. Nat. Commun. *12*, 4843. https://doi.org/10.1038/s41467-021-25154-w.
22. Mirman, Z., Sasi, N.K., King, A., Chapman, J.R., and de Lange, T. (2022). 53BP1-shieldin-dependent DSB processing in BRCA1-deficient cells requires CST-Polalpha-primase fill-in synthesis. Nat. Cell Biol. *24*, 51–61. https://doi.org/10.1038/s41556-021-00812-9.
23. King, A., Reichl, P.I., Metson, J.S., Parker, R., Munro, D., Oliveira, C., Sommerova, L., Becker, J.R., Biggs, D., Preece, C., et al. (2025). Shieldin and CST co-orchestrate DNA polymerase-dependent tailed-end joining reactions independently of 53BP1-governed repair pathway choice. Nat. Struct. Mol. Biol. *32*, 86–97. https://doi.org/10.1038/s41594-024-01381-9.
24. Hara, T., Nakaoka, H., Miyoshi, T., and Ishikawa, F. (2023). The CST complex facilitates cell survival under oxidative genotoxic stress. PLoS One *18*, e0289304. https://doi.org/10.1371/journal.pone.0289304.
25. Wysong, B.C., Schuck, P.L., Sridharan, M., Carrison, S., Murakami, Y., Balakrishnan, L., and Stewart, J.A. (2024). Human CST Stimulates Base Excision Repair to Prevent the Accumulation of Oxidative DNA Damage. J. Mol. Biol. *436*, 168672. https://doi.org/10.1016/j.jmb.2024.168672.
26. Calvo, O., Grandin, N., Jordán-Pla, A., Miñambres, E., González-Polo, N., Pérez-Ortín, J.E., and Charbonneau, M. (2019). The telomeric Cdc13-Stn1-Ten1 complex regulates RNA polymerase II transcription. Nucleic Acids Res. *47*, 6250–6268. https://doi.org/10.1093/nar/gkz279.
27. Horigome, C., Oma, Y., Konishi, T., Schmid, R., Marcomini, I., Hauer, M. H., Dion, V., Harata, M., and Gasser, S.M. (2014). SWR1 and INO80 chromatin remodelers contribute to DNA double-strand break perinuclear anchorage site choice. Mol. Cell *55*, 626–639. https://doi.org/10.1016/j.molcel.2014.06.027.
28. Oza, P., Jaspersen, S.L., Miele, A., Dekker, J., and Peterson, C.L. (2009). Mechanisms that regulate localization of a DNA double-strand break to the nuclear periphery. Genes Dev. *23*, 912–927. https://doi.org/10.1101/gad.1782209.
29. Zhang, W., and Durocher, D. (2010). De novo telomere formation is suppressed by the Mec1-dependent inhibition of Cdc13 accumulation at DNA breaks. Genes Dev. *24*, 502–515. https://doi.org/10.1101/gad.1869110.
30. Kramer, K.M., and Haber, J.E. (1993). New telomeres in yeast are initiated with a highly selected subset of TG1-3 repeats. Genes Dev. *7*, 2345–2356.
31. Diede, S.J., and Gottschling, D.E. (1999). Telomerase-mediated telomere addition in vivo requires DNA primase and DNA polymerases alpha and delta. Cell *99*, 723–733.
32. Diede, S.J., and Gottschling, D.E. (2001). Exonuclease activity is required for sequence addition and Cdc13p loading at a de novo telomere. Curr. Biol. *11*, 1336–1340.
33. Shao, Y., Lu, N., Wu, Z., Cai, C., Wang, S., Zhang, L.L., Zhou, F., Xiao, S., Liu, L., Zeng, X., et al. (2018). Creating a functional single-chromosome yeast. Nature *560*, 331–335. https://doi.org/10.1038/s41586-018-0382-x.
34. Wu, Z.J., Liu, J.C., Man, X., Gu, X., Li, T.Y., Cai, C., He, M.H., Shao, Y., Lu, N., Xue, X., et al. (2020). Cdc13 is predominant over Stn1 and Ten1 in preventing chromosome end fusions. eLife *9*, e53144. https://doi.org/10.7554/eLife.53144.
35. Shao, Y., Lu, N., Cai, C., Zhou, F., Wang, S., Zhao, Z., Zhao, G., Zhou, J.Q., Xue, X., and Qin, Z. (2019). A single circular chromosome yeast. Cell Res. *29*, 87–89. https://doi.org/10.1038/s41422-018-0110-y.
36. Wilson, T.E., and Lieber, M.R. (1999). Efficient processing of DNA ends during yeast nonhomologous end joining. Evidence for a DNA polymerase beta (Pol4)-dependent pathway. J. Biol. Chem. *274*, 23599–23609. https://doi.org/10.1074/jbc.274.33.23599.
37. Tseng, H.M., and Tomkinson, A.E. (2002). A physical and functional interaction between yeast Pol4 and Dnl4-Lif1 links DNA synthesis and ligation in nonhomologous end joining. J. Biol. Chem. *277*, 45630–45637. https://doi.org/10.1074/jbc.M206861200.
38. Lemos, B.R., Kaplan, A.C., Bae, J.E., Ferrazzoli, A.E., Kuo, J., Anand, R.P., Waterman, D.P., and Haber, J.E. (2018). CRISPR/Cas9 cleavages in budding yeast reveal templated insertions and strand-specific insertion/deletion profiles. Proc. Natl. Acad. Sci. USA *115*, E2040–E2047. https://doi.org/10.1073/pnas.1716855115.
39. Kosicki, M., Tomberg, K., and Bradley, A. (2018). Repair of double-strand breaks induced by CRISPR-Cas9 leads to large deletions and complex rearrangements. Nat. Biotechnol. *36*, 765–771. https://doi.org/10.1038/nbt.4192.
40. Owens, D.D.G., Caulder, A., Frontera, V., Harman, J.R., Allan, A.J., Bucakci, A., Greder, L., Codner, G.F., Hublitz, P., McHugh, P.J., et al. (2019). Microhomologies are prevalent at Cas9-induced larger deletions. Nucleic Acids Res. *47*, 7402–7417. https://doi.org/10.1093/nar/gkz459.
41. van Schendel, R., Schimmel, J., and Tijsterman, M. (2022). SIQ: easy quantitative measurement of mutation profiles in sequencing data. NAR Genom. Bioinform. *4*, lqac063. https://doi.org/10.1093/nargab/lqac063.
42. Hussmann, J.A., Ling, J., Ravisankar, P., Yan, J., Cirincione, A., Xu, A., Simpson, D., Yang, D., Bothmer, A., Cotta-Ramusino, C., et al. (2021). Mapping the genetic landscape of DNA double-strand break repair. Cell *184*, 5653–5669. https://doi.org/10.1016/j.cell.2021.10.002.
43. Emerson, C.H., Lopez, C.R., Ribes-Zamora, A., Polleys, E.J., Williams, C. L., Yeo, L., Zaneveld, J.E., Chen, R., and Bertuch, A.A. (2018). Ku DNA End-Binding Activity Promotes Repair Fidelity and Influences End-Processing During Nonhomologous End-Joining in Saccharomyces cerevisiae. Genetics *209*, 115–128. https://doi.org/10.1534/genetics.117.300672.
44. Lee, K., and Lee, S.E. (2007). Saccharomyces cerevisiae Sae2- and Tel1-dependent single-strand DNA formation at DNA break promotes microhomology-mediated end joining. Genetics *176*, 2003–2014. https://doi.org/10.1534/genetics.107.076539.
45. Ma, J.L., Kim, E.M., Haber, J.E., and Lee, S.E. (2003). Yeast Mre11 and Rad1 proteins define a Ku-independent mechanism to repair double-strand breaks lacking overlapping end sequences. Mol. Cell Biol. *23*, 8820–8828.
46. Meyer, D., Fu, B.X.H., and Heyer, W.D. (2015). DNA polymerases delta and lambda cooperate in repairing double-strand breaks by microhomology-mediated end-joining in Saccharomyces cerevisiae. Proc. Natl. Acad. Sci. USA *112*, E6907–E6916. https://doi.org/10.1073/pnas.1507833112.
47. Deng, S.K., Gibb, B., de Almeida, M.J., Greene, E.C., and Symington, L.S. (2014). RPA antagonizes microhomology-mediated repair of DNA double-strand breaks. Nat. Struct. Mol. Biol. *21*, 405–412. https://doi.org/10.1038/nsmb.2786.
48. Zierhut, C., and Diffley, J.F.X. (2008). Break dosage, cell cycle stage and DNA replication influence DNA double strand break response. EMBO J. *27*, 1875–1885. https://doi.org/10.1038/emboj.2008.111.
49. Puglisi, A., Bianchi, A., Lemmens, L., Damay, P., and Shore, D. (2008). Distinct roles for yeast Stn1 in telomere capping and telomerase inhibition. EMBO J. *17*, 2328–2339.
50. Sun, J., Yang, Y., Wan, K., Mao, N., Yu, T.Y., Lin, Y.C., DeZwaan, D.C., Freeman, B.C., Lin, J.J., Lue, N.F., and Lei, M. (2011). Structural bases of dimerization of yeast telomere protein Cdc13 and its interaction with the catalytic subunit of DNA polymerase alpha. Cell Res. *21*, 258–274. https://doi.org/10.1038/cr.2010.138.
51. Lee, S.E., Moore, J.K., Holmes, A., Umezu, K., Kolodner, R.D., and Haber, J.E. (1998). Saccharomyces Ku70, mre11/rad50 and RPA proteins regulate adaptation to G2/M arrest after DNA damage. Cell *94*, 399–409.
52. Clerici, M., Mantiero, D., Guerini, I., Lucchini, G., and Longhese, M.P. (2008). The Yku70-Yku80 complex contributes to regulate double-strand

break processing and checkpoint activation during the cell cycle. EMBO Rep. *9*, 810–818.

53. Moore, J.K., and Haber, J.E. (1996). Cell cycle and genetic requirements of two pathways of nonhomologous end-joining repair of double-strand breaks in Saccharomyces cerevisiae. Mol. Cell Biol. *16*, 2164–2173.
54. Shim, E.Y., Ma, J.L., Oum, J.H., Yanez, Y., and Lee, S.E. (2005). The yeast chromatin remodeler RSC complex facilitates end joining repair of DNA double-strand breaks. Mol. Cell Biol. *25*, 3934–3944. https://doi.org/10.1128/MCB.25.10.3934-3944.2005.
55. Moore, J.K., and Haber, J.E. (1996). Capture of retrotransposon DNA at the sites of chromosomal double- strand breaks. Nature *383*, 644–646.
56. Ricchetti, M., Fairhead, C., and Dujon, B. (1999). Mitochondrial DNA repairs double-strand breaks in yeast chromosomes. Nature *402*, 96–100.
57. Setiaputra, D., and Durocher, D. (2019). Shieldin - the protector of DNA ends. EMBO Rep. *20*, e47560. https://doi.org/10.15252/embr.201847560.
58. Hardy, C.F., Sussel, L., and Shore, D. (1992). A RAP1-interacting protein involved in transcriptional silencing and telomere length regulation. Genes Dev. *6*, 801–814.
59. Badugu, S., Dhyani, K.M., Thakur, M., and Muniyappa, K. (2024). Saccharomyces cerevisiae Rev7 promotes non-homologous end-joining by blocking Mre11 nuclease and Rad50's ATPase activities and homologous recombination. eLife *13*, RP96933. https://doi.org/10.7554/eLife.96933.
60. Petreaca, R.C., Chiu, H.C., Eckelhoefer, H.A., Chuang, C., Xu, L., and Nugent, C.I. (2006). Chromosome end protection plasticity revealed by Stn1p and Ten1p bypass of Cdc13p. Nat. Cell Biol. *8*, 748–755. https://doi.org/10.1038/ncb1430.
61. Ge, Y., Wu, Z., Chen, H., Zhong, Q., Shi, S., Li, G., Wu, J., and Lei, M. (2020). Structural insights into telomere protection and homeostasis regulation by yeast CST complex. Nat. Struct. Mol. Biol. *27*, 752–762. https://doi.org/10.1038/s41594-020-0459-8.
62. Bazzano, D., Lomonaco, S., and Wilson, T.E. (2021). Mapping yeast mitotic 5' resection at base resolution reveals the sequence and positional dependence of nucleases in vivo. Nucleic Acids Res. *49*, 12607–12621. https://doi.org/10.1093/nar/gkab597.
63. Zahid, S., Seif El Dahan, M., Iehl, F., Fernandez-Varela, P., Le Du, M.H., Ropars, V., and Charbonnier, J.B. (2021). The Multifaceted Roles of Ku70/80. Int. J. Mol. Sci. *22*, 4134. https://doi.org/10.3390/ijms22084134.
64. Frank-Vaillant, M., and Marcand, S. (2002). Transient stability of DNA ends allows nonhomologous end joining to precede homologous recombination. Mol. Cell *10*, 1189–1199.
65. Kramer, K.M., Brock, J.A., Bloom, K., Moore, J.K., and Haber, J.E. (1994). Two different types of double-strand breaks in Saccharomyces cerevisiae are repaired by similar RAD52-independent, nonhomologous recombination events. Mol. Cell Biol. *14*, 1293–1301.
66. Roth, D.B., and Wilson, J.H. (1986). Nonhomologous recombination in mammalian cells: role for short sequence homologies in the joining reaction. Mol. Cell Biol. *6*, 4295–4304. https://doi.org/10.1128/mcb.6.12.4295-4304.1986.
67. Longtine, M.S., McKenzie, A., 3rd, Demarini, D.J., Shah, N.G., Wach, A., Brachat, A., Philippsen, P., and Pringle, J.R. (1998). Additional modules for versatile and economical PCR-based gene deletion and modification in Saccharomyces cerevisiae. Yeast *14*, 953–961.
68. Anand, R., Beach, A., Li, K., and Haber, J. (2017). Rad51-mediated double-strand break repair and mismatch correction of divergent substrates. Nature *544*, 377–380. https://doi.org/10.1038/nature22046.
69. Christianson, T.W., Sikorski, R.S., Dante, M., Shero, J.H., and Hieter, P. (1992). Multifunctional yeast high-copy-number shuttle vectors. Gene *110*, 119–122.
70. Fallet, E., Jolivet, P., Soudet, J., Lisby, M., Gilson, E., and Teixeira, M.T. (2014). Length-dependent processing of telomeres in the absence of telomerase. Nucleic Acids Res. *42*, 3648–3665. https://doi.org/10.1093/nar/gkt1328.
71. Coutelier, H., Xu, Z., Morisse, M.C., Lhuillier-Akakpo, M., Pelet, S., Charvin, G., Dubrana, K., and Teixeira, M.T. (2018). Adaptation to DNA damage checkpoint in senescent telomerase-negative cells promotes genome instability. Genes Dev. *32*, 1499–1513. https://doi.org/10.1101/gad.318485.118.
72. Langmead, B., and Salzberg, S.L. (2012). Fast gapped-read alignment with Bowtie 2. Nat. Methods *9*, 357–359. https://doi.org/10.1038/nmeth.1923.
73. Cournac, A., Marie-Nelly, H., Marbouty, M., Koszul, R., and Mozziconacci, J. (2012). Normalization of a chromosomal contact map. BMC Genom. *13*, 436. https://doi.org/10.1186/1471-2164-13-436.
74. Serizay, J., Matthey-Doret, C., Bignaud, A., Baudry, L., and Koszul, R. (2024). Orchestrating chromosome conformation capture analysis with Bioconductor. Nat. Commun. *15*, 1072. https://doi.org/10.1038/s41467-024-44761-x.

STAR★METHODS

KEY RESOURCES TABLE

REAGENT or RESOURCE	SOURCE	IDENTIFIER
Chemicals, peptides, and recombinant proteins		
Nucleic Acid Detection Blocking Buffer	ThermoFisher Scientific	Cat#89880A
Streptavidin, Alkaline Phosphatase Conjugate	Invitrogen	Cat#S921
PerfectHyb™ Plus Hybridization Buffer	Sigma-Aldrich	Cat#H7033-125ML
CDP-*Star*™ Substrate	ThermoFisher Scientific	Cat#T2146
Dynabeads™ Streptavidin C1	Fisher Scientific	Cat#10202333
Critical commercial assays		
Master Mix Fast SYBR™ Green	ThermoFisher Scientific	Cat#4385612
Arima HiC kit	Arima Genomics	Cat#A510008
Invitrogen Collibri PS DNA Library Prep Kit	ThermoFisher Scientific	Cat#A38612024
Deposited data		
New data: high-throughput sequencing raw data for the mutation signature analyses and Hi-C maps	This paper	ENA Accession number: PRJEB88514
Experimental models: Organisms/strains		
S. cerevisiae: Strain background: BY4742. And derivatives (including SY13 and SY14).	Shao, Y., Lu, N., Wu, Z., Cai, C., Wang, S., Zhang, L.L., Zhou, F., Xiao, S., Liu, L., Zeng, X. et al. (2018). Creating a functional single-chromosome yeast. Nature *560*, 331–335.	N/A
S. cerevisiae: Strain background: BY4742. Other mutant strains, including the circular chromosome strains and derivatives.	This paper	N/A
S. cerevisiae: Strain background: JKM179. Derived mutant strains.	This paper	N/A
Oligonucleotides		
See Table S3	This paper	N/A
Software and algorithms		
SIQ software	van Schendel, R., Schimmel, J., and Tijsterman, M. (2022). SIQ: easy quantitative measurement of mutation profiles in sequencing data. NAR Genom Bioinform *4*, lqac063	https://siq.researchlumc.nl/SIQPlotteR/
Hicstuff	Cyril Matthey-Doret, Lyam Baudry, Amaury Bignaud, Axel Cournac, Remi-Montagne, Nadège Guiglielmoni, Théo Foutel Rodier and Vittore F. Scolari. 2020. hicstuff: Simple library/pipeline to generate and handle Hi-C data. Zenodo. http://doi.org/10.5281/zenodo.4066363	https://github.com/koszullab/hicstuff
OHCA	Serizay, J., Matthey-Doret, C., Bignaud, A., Baudry, L., and Koszul, R. (2024). Orchestrating chromosome conformation capture analysis with Bioconductor. Nat Commun *15*, 1072.	https://bioconductor.org/books/release/OHCA/

EXPERIMENTAL MODEL AND STUDY PARTICIPANT DETAILS

Yeast strains and plasmids

All *S. cerevisiae* (RRID: 4932) strains used in this work are listed in Table S1. Most are from the BY4742 background, except for JKM179 and derivatives. The strain SY14 with a single linear chromosome, SY13 with 2 chromosomes and the parental BY4742 are kind gifts from Prof. Jin-Qiu Zhou and colleagues.[33] Deletion strains were created using standard PCR-based methods.[67] Point mutations were generated using Cas9-mediated gene editing.[68] Strains were grown in rich YPD (yeast extract, peptone, dextrose) or synthetic complete (SC) media at 30°C. All plasmids used in this work are listed in Table S2.

Circular chromosome strain

To create the single circular chromosome strain, we used plasmid pJH2970 (gift from Jim Haber[68]) containing the Cas9 gene and a site to clone a guide RNA sequence as a base to build plasmid pZX026 in which the sequences to express two guide RNAs were cloned. They were designed to target the subtelomere of chromosome X-R at position 743778 and the subtelomere of chromosome XVI-L at position 17419 (using the initial chromosome numbers and coordinates before they were fused). Transformation of strain SY14 with pZX026 simultaneously with a chimeric double-stranded DNA (Table S3) homologous to both subtelomeres allowed the recovery of transformants that have cut the two subtelomeres and recombined them together, thus yielding a strain with a single circular chromosome, yZX168 (Figures S1A–S1C).

METHOD DETAILS

Spot assay and survival assay

Strains transformed with plasmid pZX013 or pZX010 to target Cas9 to the 5′ UTR of *LYS2* or the 5′ UTR of *URA3*, respectively, were first grown overnight in YPD containing hygromycin (200 μg/mL) at 30°C, then diluted at optical density $OD_{600\ nm} = 0.7$ in YPLG (yeast extract, peptone, 2% lactic acid, 3% glycerol) media and grown for an additional 24h. For the strains bearing the HO cut (JKM179 and derivatives), the HO gene is already under the control of the *GAL10* promoter and no plasmid was transformed into the strains. Cas9 or HO expression was induced by plating the cells on rich solid media (or SC media lacking the appropriate amino acid) containing 2% galactose or by addition of 2% galactose in the liquid media. As a control, we plated cells from the same culture on 2% glucose-containing plates or added 2% glucose in the liquid media. For spot assays, 10-fold serial dilutions of the liquid culture were performed before depositing 5 μL per spot. For survival assays, after a first estimate of the survival frequency for each strain, the appropriate dilution of the culture was plated on galactose-containing plates so as to obtain between 50 and 500 surviving colonies. An additional 100-fold dilution was plated on YPD plates to calculate the frequency.

Resection assays by quantitative PCR

The yeast strains transformed with plasmid pZX013 were grown as for a survival assay and the Cas9 DSB was induced in liquid media for 6h by addition of 2% galactose. The quantitative resection assay was performed as described in.[48] Briefly, genomic DNA was extracted by standard phenol chloroform method and digested by FokI, which cleaves 0.95 kb away from the DSB site but is unable to cleave ssDNA. Using primers flanking the FokI restriction site, qPCR measured the amount of ssDNA relative to the DNA present at each timepoint, by following the formula: $\%resected = (100/((1 + 2^{\Delta C_t})/2))/f$, where ΔC_t is the difference in cycles between FokI-digested and undigested samples, and f is the fraction cut by Cas9 determined by qPCR with primers flanking the cut site. All DNA samples were normalized using qPCR primers targeting *ACT1*.

Plasmid religation assay

The plasmid religation assay was performed as reported in.[34] Briefly, plasmid pRS426[69] containing *URA3* as a selection marker was linearized *in vitro* with EcoRI and the linear form was migrated by gel electrophoresis, excised from the gel and purified. 60 ng of linear or circular plasmid was transformed into the WT circular chromosome strain, *dnl4Δ* mutant and *stn1Δ* mutant, using selective plates lacking uracil. In parallel, to measure plating efficiency, a 6.8×10^4-fold dilution was plated on non-selective YPD media. To calculate the transformation efficiency, the number of colonies on selective media was divided by the number of colonies on YPD multiplied by the dilution factor.

Southern blot

To verify subtelomere fusion after creating the circular chromosome, a Southern blot was performed as in,[70] but the genomic DNA was digested with HindIII and NdeI, and the radiolabeled probe was generated by random priming on a purified PCR fragment overlapping the fusion site (primers used: oT1735 and oT1736, see Table S3). The terminal restriction fragment (XhoI restriction) Southern blot, used to detect telomeres, was performed as described in,[71] except that instead of a radioactive probe, an oligonucleotide probe biotinylated at both ends was used (5′-GGGTGTGGGTGTGTGTGGTGGG-3′; Eurofins Genomics) and detected by chemiluminescence. More specifically, genomic DNA was extracted from yeast samples using a phenol chloroform purification method. Between 500 ng and 1 μg of DNA was then digested with the restriction enzymes, the products were ethanol-precipitated, resuspended in loading buffer and resolved on a 1% agarose gel. The gel was then soaked in a denaturation bath (0.4 NaOH, 1 M NaCl) for

20 min and transferred by capillarity to a nylon membrane (Hybond XL, GE Healthcare). The membrane was hybridized using the PerfectHyb protocol (Merck). After hybridization of the probe, the membrane was washed 3 × 5 min in wash buffer (58 mM Na_2HPO_4, 17 mM NaH_2PO_4, 68 mM NaCl, 0.1% SDS). The membrane was next processed for detection with 3 successive incubations (5, 5 and 30 min) in blocking buffer (Thermo Scientific, Nucleic Acid Detection Blocking Buffer) before a 30 min incubation with alkaline phosphatase-conjugated streptavidin (Invitrogen) diluted in blocking buffer (0.4 μg/mL). The membrane was then washed again 3 × 5 min in wash buffer, incubated 2 × 2 min in assay buffer (0.1 M Tris, 0.1 M NaCl pH9.5) and 5 min in CDP-Star substrate (Applied Biosystems) before imaging with a GelDoc system (BioRad).

Mutation signature analysis

Strains transformed with plasmid pZX013 or pZX010 were cultivated and plated on galactose plates as for survival assays, except that more cells were plated so as to obtain hundreds to thousands of surviving colonies. After pooling of the colonies and genomic DNA extraction, a 231-bp amplicon around the Cas9 DSB site was generated using primer containing adapters and multiplexing barcodes for Illumina sequencing (Table S3). The libraries were sequenced on an Illumina MiSeq (2 x 250 bp) platform. Analysis of the mutation signature around the Cas9 DSB was performed as described in,[21] using the SIQ software.[41] The resulting analyses are reported in Data S2 and the SIQ classification and characterization of mutational events were used to quantify each type of mutation (Data S1).

Hi-C procedure and sequencing

10^7 cells in 150 mL of YPD media were fixed with 3% formaldehyde for 20 min at 30°C before the reaction was quenched by adding glycine to 0.125 M final concentration for 20 min at room temperature. Hi-C experiments were performed with a Hi-C kit (Arima Genomics) with a double DpnII + HinfI restriction digestion following manufacturer instructions. Samples were purified using AMPure XP beads (Beckman A63882), recovered in 120 μL H_2O and sonicated using Covaris (~300 bp) in Covaris microTUBE (Covaris, 520045). Biotinylated DNA was loaded on Dynabeads™ Streptavidin C1 (Fisher Scientific, 10202333). Preparation of the samples for paired-end sequencing on an Illumina NextSeq500 (2x35 bp) was performed using Invitrogen Collibri PS DNA Library Prep Kit for Illumina and following manufacturer instructions. Paired-end sequencing on an Illumina NextSeq500 (2 x 35 bp) was performed.

Hi-C processing

Reads were aligned and contact maps generated and processed using Hicstuff (https://github.com/koszullab/hicstuff). Briefly, pairs of reads were aligned iteratively and independently using Bowtie2[72] in its most sensitive mode against the reference genome CP029160.1 (https://www.ncbi.nlm.nih.gov/nuccore/CP029160;[33]). Each uniquely mapped read was assigned to a restriction fragment. Quantification of pairwise contacts between restriction fragments was performed with default parameters: uncuts, loops and circularization events were filtered as described in.[73] PCR duplicates (defined as multiple pairs of reads positioned at the exact same position) were discarded. Pairs were binned at 16 kb resolution and contact maps (in mcool format) were generated using OHCA.[74]

QUANTIFICATION AND STATISTICAL ANALYSIS

Analysis softwares are described in the method details of the corresponding experiment and are listed in the key resources table. All statistical analyses were performed using MATLAB R2020b. The specific statistical tests used are indicated in the corresponding figure legend with the number of independent samples n. Statistical significance was defined using a *p*-value threshold at 0.05. When relevant, descriptive statistics (mean and standard deviation) are indicated in the figure legends.

Cell Genomics, Volume *5*

Supplemental information

The CST complex mediates a post-resection non-homologous end joining repair pathway and promotes local deletions in *Saccharomyces cerevisiae*

Oana Ilioaia, Liébaut Dudragne, Clémentine Brocas, Léa Meneu, Romain Koszul, Karine Dubrana, and Zhou Xu

SUPPLEMENTAL INFORMATION

Title: The CST complex mediates a post-resection non-homologous end-joining repair pathway and promotes local deletions in *Saccharomyces cerevisiae*

Authors: Oana Ilioaia[1], Liébaut Dudragne[1], Clémentine Brocas[2], Léa Meneu[3,4], Romain Koszul[3], Karine Dubrana[2] & Zhou Xu[1,*]

Affiliations:

[1]Sorbonne Université, CNRS, UMR7238, Institut de Biologie Paris-Seine, Laboratory of Computational, Quantitative and Synthetic Biology, CQSB, 75005 Paris, France.

[2]Université Paris Cité, Inserm, CEA, Stabilité Génétique Cellules Souches et Radiations, F-92260 Fontenay-aux-Roses, France.

[3]Institut Pasteur, CNRS UMR3525, Université Paris Cité, Unité Régulation Spatiale des Génomes, 75015 Paris, France.

[4]Sorbonne Université, Collège Doctoral.

*Lead contact

Correspondence:

zhou.xu@sorbonne-universite.fr

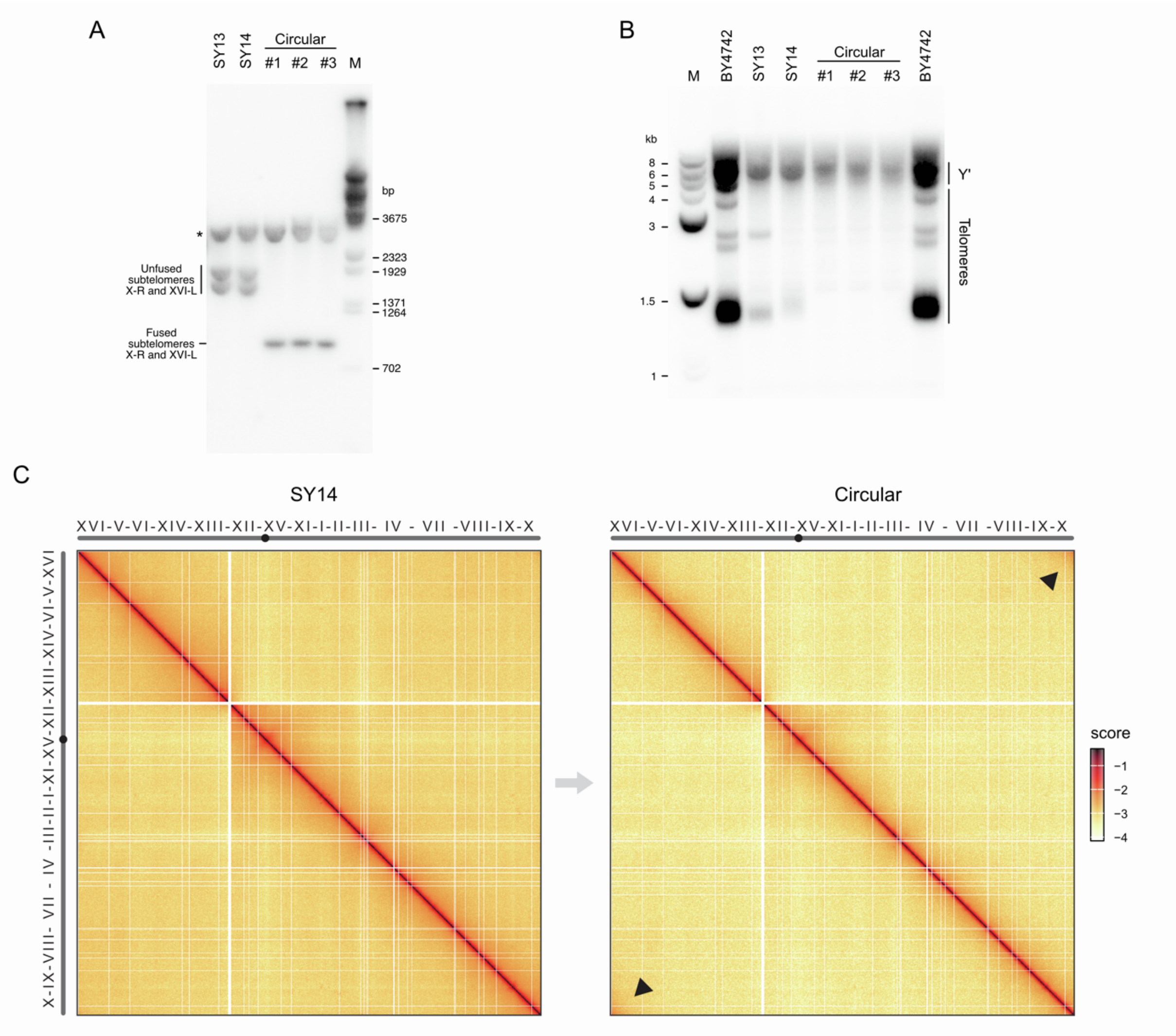

Supplemental Figure S1. Single chromosome circularization. Related to Figure 1.

(A) Southern blot for detecting the fusion between the two subtelomeres of SY14. HindIII- and NdeI-digested genomic DNA of the indicated strains was migrated, transferred and probed with a chimeric radiolabeled oligonucleotide complementary to both subtelomeres. Three independent cultures of the circular chromosome strain were tested. M: molecular weight marker (λ DNA, BstEII digest). *: non-specific band.

(B) Terminal restriction fragment Southern blot. XhoI-digested genomic DNA of the indicated strains was migrated, transferred and probed with a telomeric probe. M: molecular weight marker.

(C) Normalized Hi-C contact maps of the linear strain SY14 (left) and the single circular chromosome strain (right) with 16-kb resolution. Low to high interaction frequencies are depicted by a color

spectrum from light yellow to red. Arrows indicate contacts associated with chromosome circularization.

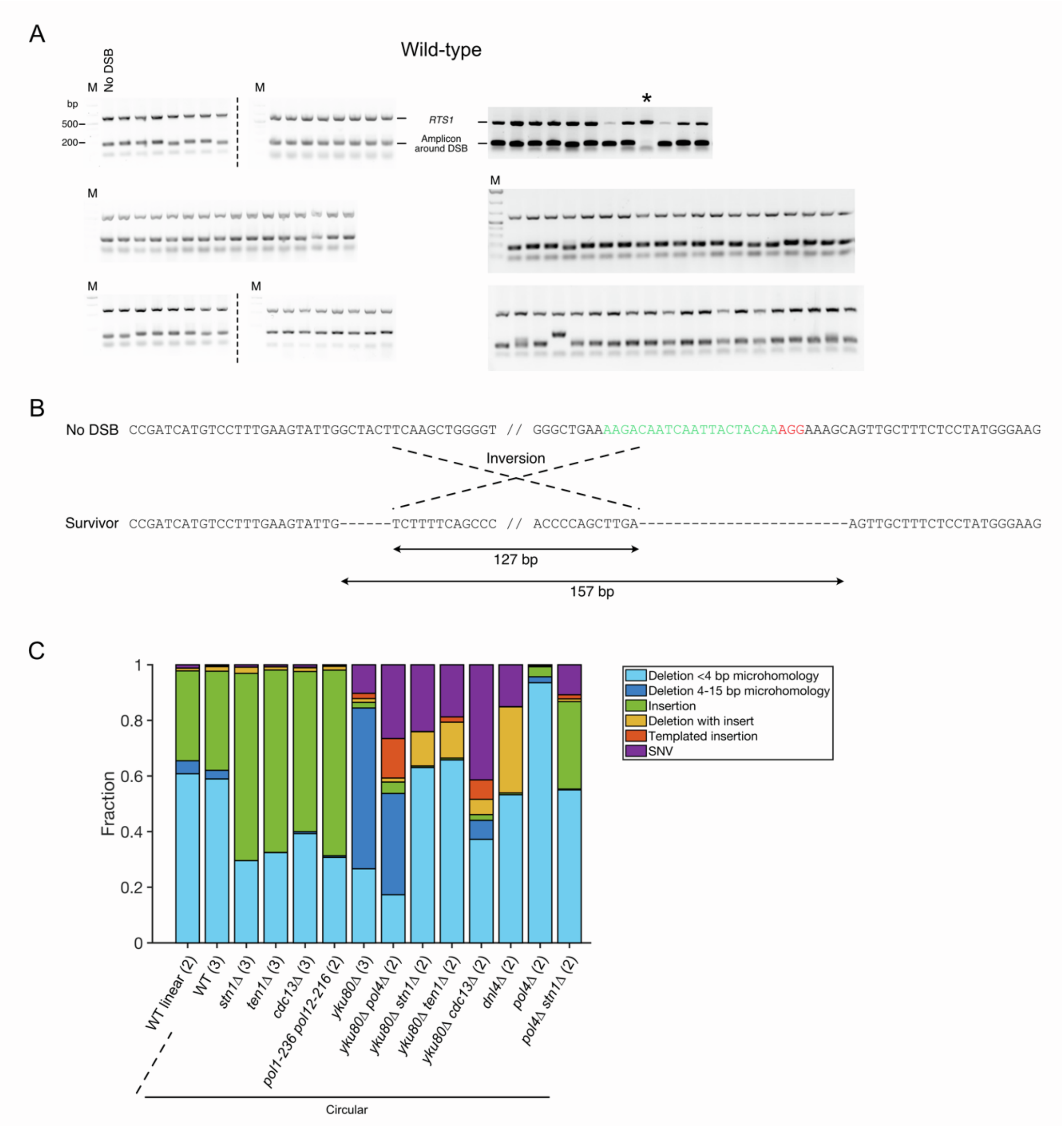

Supplemental Figure S2. Sequencing of an amplicon around DSB captures nearly all repair event. Related to Figure 2.

(A) Multiplex PCR showing a 176-bp fragment around the DSB ("Amplicon around DSB") and a fragment in *RTS1*. *: unproductive PCR around the DSB. M: molecular weight marker.

(B) Schematic representation of the single event not captured by PCR in (A). PCR mapping and sequencing of the junction revealed an inversion.

(C) Mutation signature of the indicated strains as shown in Figure 2A, but represented in fraction of each type, without normalization by survival rate.

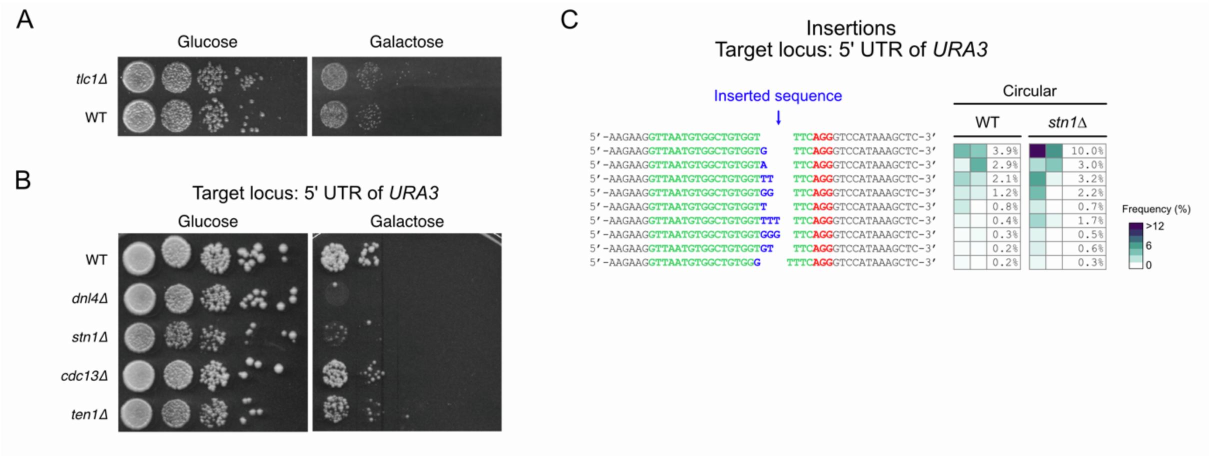

Supplemental Figure S3. CST's role is telomerase-independent and verified at another DSB locus. Related to Figure 3.

(A) Spot assay as in Figure 1D with the indicated strains. The image comes from the same plate as Figure 5A and thus shares the same WT.

(B) Spot assay as in Figure 1D with the indicated strains and with Cas9 targeted to the 5' UTR of *URA3* with a specific guide RNA.

(C) Heatmap of the frequency of each insertion outcome for Cas9 targeted at the 5' UTR of *URA3*, for individual experiments in WT and *stn1Δ* strains. Representation as in Figure 2B, except that only events with frequency > 0.002 are shown. See Supplemental Data 1 and 2 for the unfiltered data.

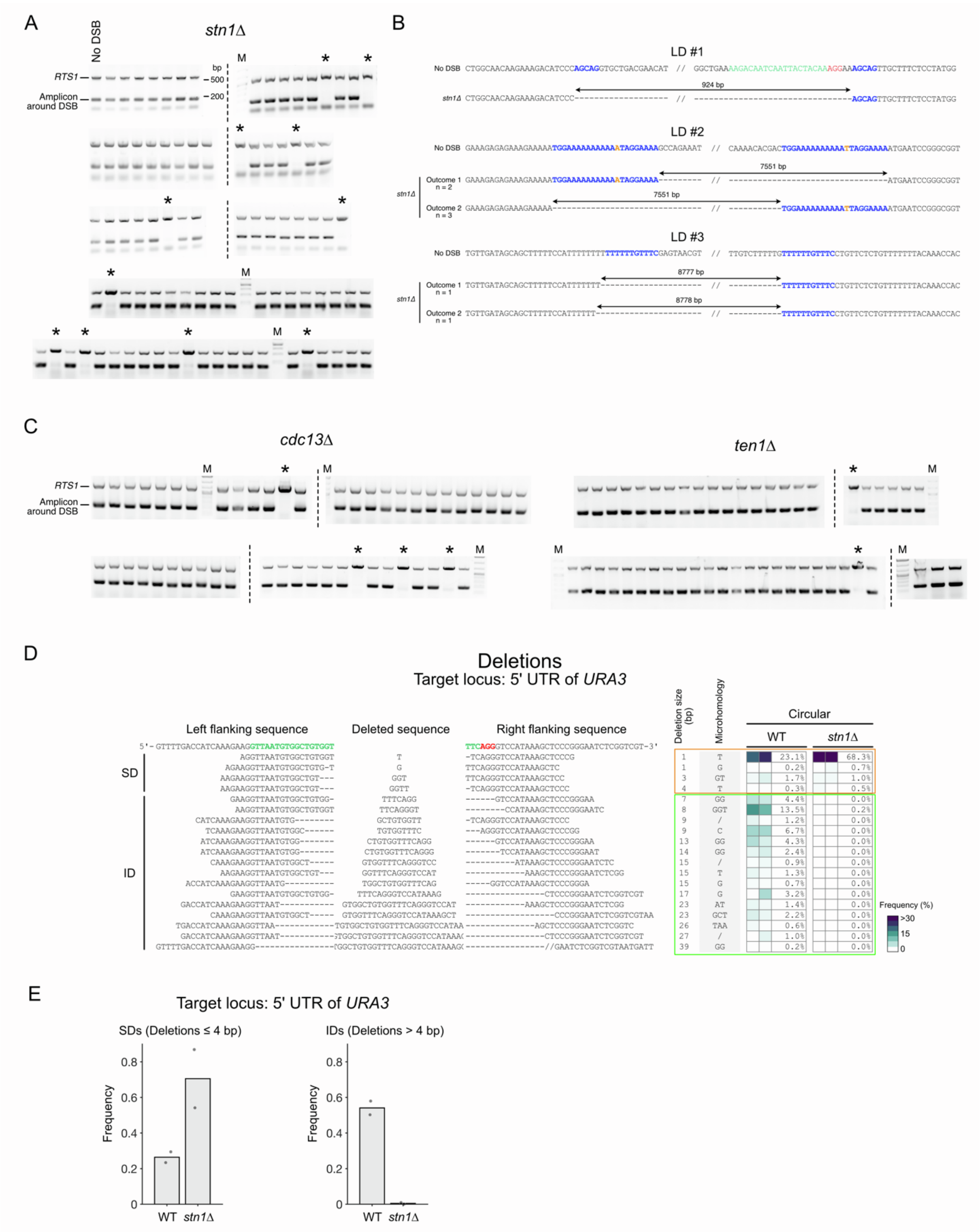

Supplemental Figure S4. LDs in CST mutants and mutation signature analysis at the DSB targeted to the 5' UTR of *URA3*. Related to Figure 4.

(A) Multiplex PCR in *stn1Δ* strain showing a 176-bp fragment around the DSB ("Amplicon around DSB") and a fragment in *RTS1*. M: molecular weight marker. * indicate unproductive PCRs around the cut site.

(B) Sequences at the junctions of 8 large deletions detected in (A), revealing LDs of 4 different sizes. The microhomologies used are shown in blue. For LD #2, the microhomology is 22-bp long with a mismatch (in orange). Mismatch repair would eventually resolve the mispairing, leading to the 2 observed outcomes. For LD #3, additional deletion of 1 or 2 bp leads to the 2 observed outcomes.

(C) Multiplex PCR as in (A) but performed on surviving colonies of *cdc13Δ* and *ten1Δ* mutants.

(D) Heatmap of the frequency of each repair outcome with a deletion for individual experiments (in columns) with the indicated strains, for Cas9 targeted at the 5'UTR of *URA3*. Only deletions that appear with a frequency of > 0.018 in at least one experiment are shown. See Supplemental Data 1 and 2 for the unfiltered data.

(E) Frequency of SDs (left) and IDs (right) for WT and *stn1Δ*, derived from (C), when the DSB is induced at the 5'UTR of *URA3*. Each dot represents an independent experiment.

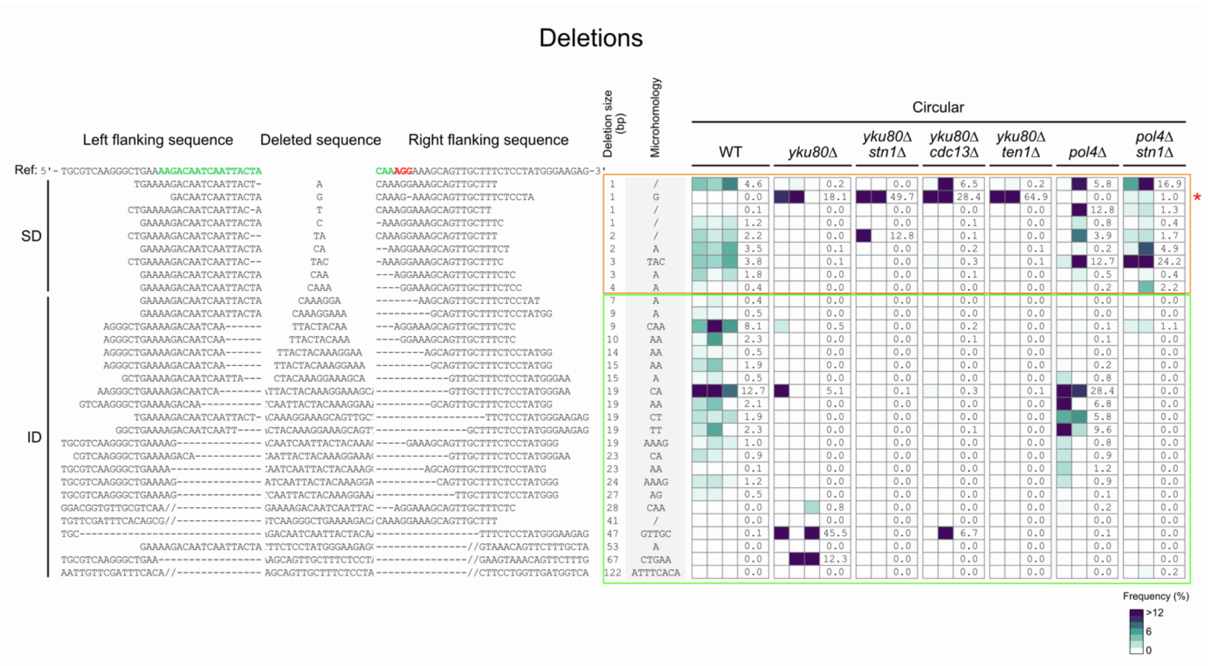

Supplemental Figure S5. CST is required for IDs present in *yku80Δ* and *pol4Δ* mutants. Related to Figure 4.

Heatmap of the frequency of each repair outcome with a deletion for individual experiments (in columns) with the indicated strains. For each strain, a column indicates the average frequency (in %) of each deletion. Red asterisk: deletion of one G located 4-5 bp away from the cut site, likely not due to the repair of the DSB. The data for the WT, *yku80Δ* and *pol4Δ* strains are reused from Figure 2C, but with a different frequency scale. See Supplemental Data 1 and 2 for the unfiltered data.

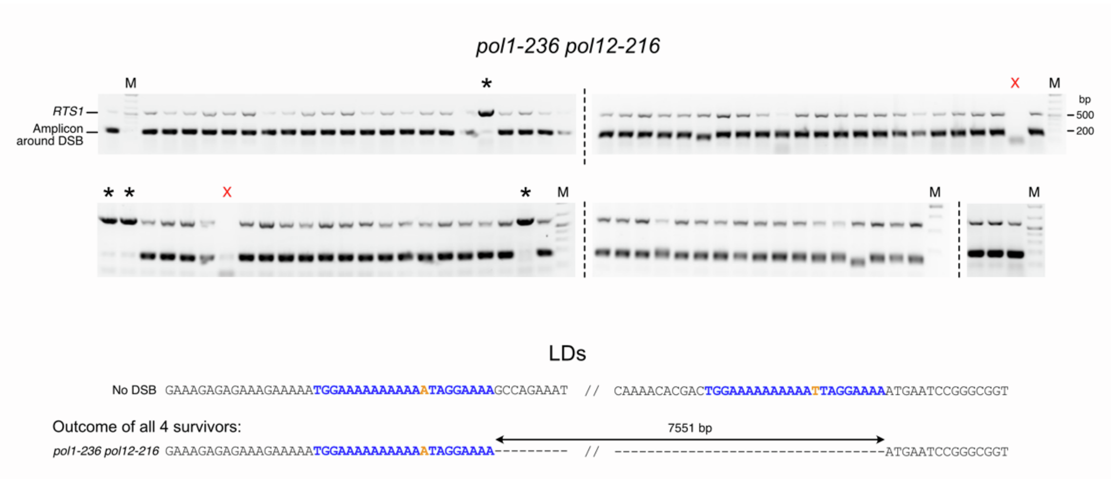

Supplemental Figure S6. Multiplex PCR assay in *pol1-236 pol12-216* mutant. Related to Figure 4.

Multiplex PCR in *pol1-236 pol12-216* strain showing a 176-bp fragment around the DSB (“Amplicon around DSB”) and a fragment in *RTS1*. * indicate unproductive PCRs around the DSB. M: molecular weight marker. Red Xs show failed PCR amplification for the control site; the corresponding samples are thus removed from analysis. (Lower part) All 4 unproductive PCRs corresponded to the same LD as in outcome 1 of LD #2 in *stn1Δ* survivor clones, in Figure S4B.

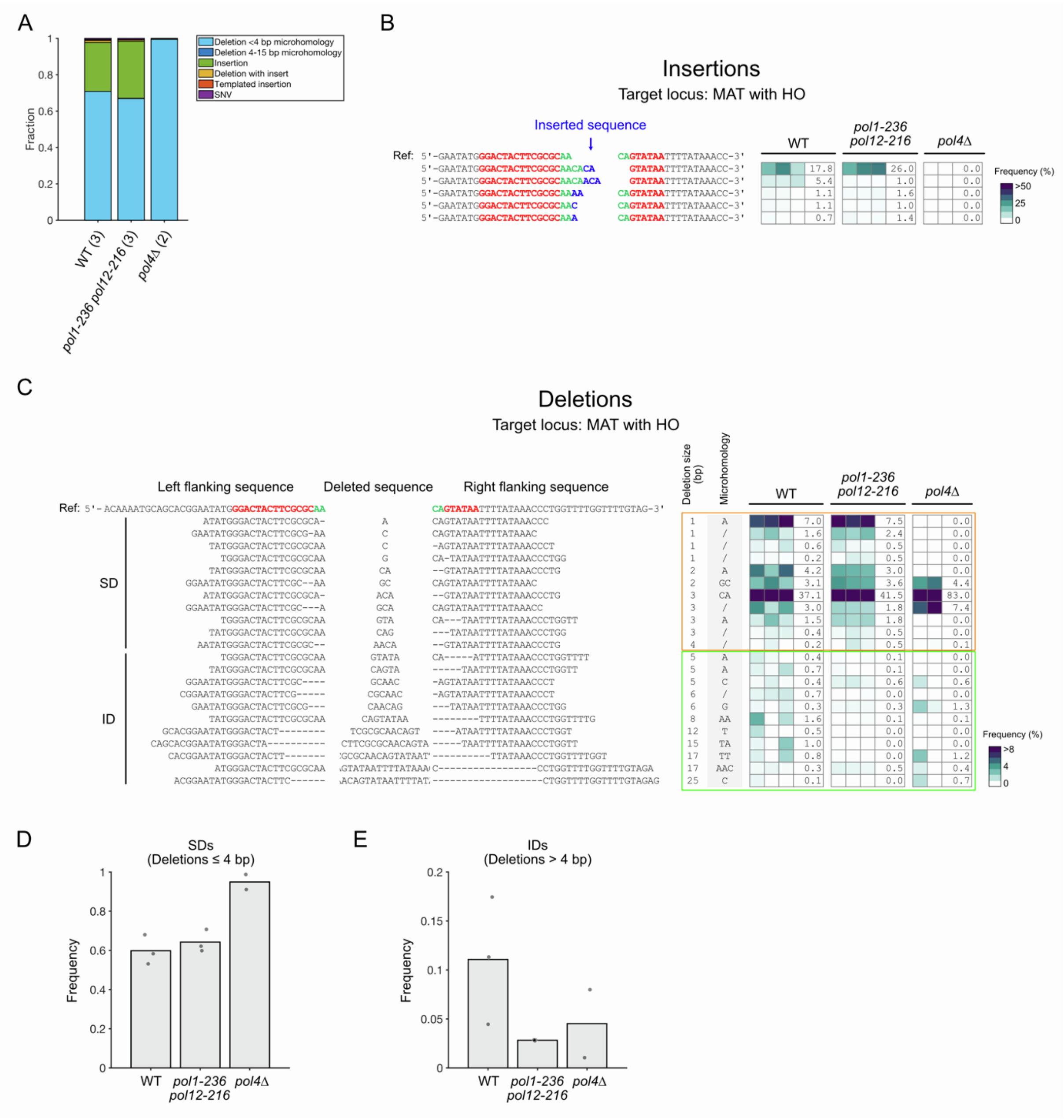

Supplemental Figure S7. CST's interaction with Polα-primase specifically affects the IDs formed after repair of an HO cut. Related to Figure 6.

(A) Mutation signature of the indicated strains in the JKM179 background allowing the induction of an HO cut at the MAT locus. Representation as in Supplemental Figure S2C.

(B) Heatmap of the frequency of each repair outcome after an HO cut with an insertion for individual experiments (in columns) with the indicated strains, represented as in Figure 2B. For each strain, a column indicates the average frequency (in %) of each insertion. HO's recognition site is colored in red

and green, with the green part corresponding to the staggered cut region. See Supplemental Data 1 and 2 for the unfiltered data.

(C) Heatmap of the frequency of each repair outcome after an HO cut with a deletion for individual experiments (in columns) with the indicated strains, represented as in Figure 2C, except for the color bar which uses a different scaling. For each strain, a column indicates the average frequency (in %) of each deletion. See Supplemental Data 1 and 2 for the unfiltered data.

(D) Frequency of SDs, *i.e.* deletions ≤ 4 bp, for the indicated strains. Each dot represents an independent experiment.

(E) Frequency of IDs, *i.e.* 5-85 bp deletions, for the indicated strains.

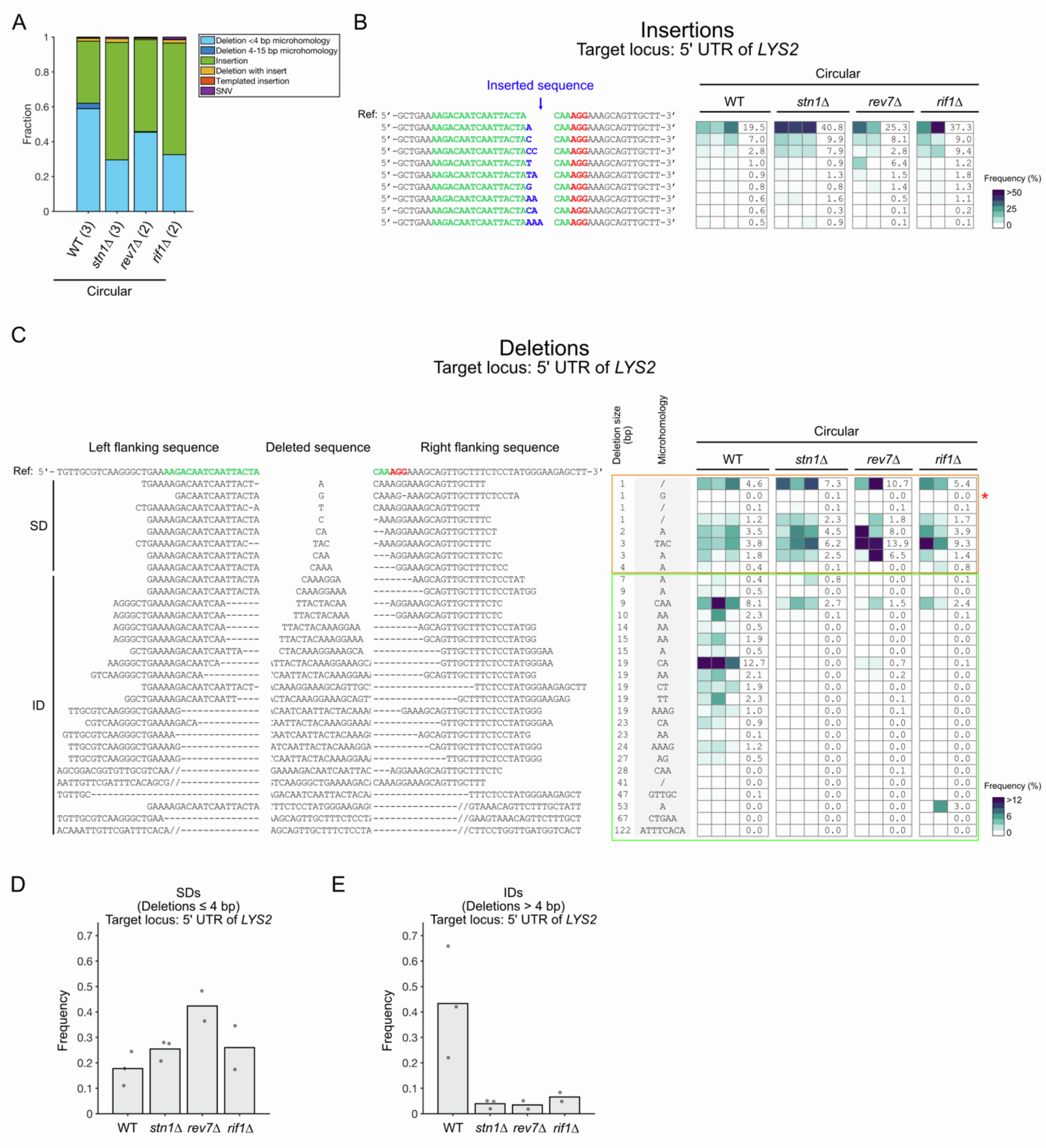

Supplemental Figure S8. Rev7 and Rif1 participate in ID formation. Related to Figure 6.

(A) Mutation signature of the indicated strains in the circular chromosome strain background. Representation as in Supplemental Figure S2C.

(B) Heatmap of the frequency of each repair outcome with an insertion for individual experiments (in columns) with the indicated strains, represented as in Figure 2B. For each strain, a column indicates the average frequency (in %) of each insertion. Data for WT and *stn1Δ* are reused from Figure 3C. See Supplemental Data 1 and 2 for the unfiltered data.

(C) Heatmap of the frequency of each repair outcome with a deletion for individual experiments (in columns) with the indicated strains, represented as in Figure 2C, except for the color bar which uses a different scaling. For each strain, a column indicates the average frequency (in %) of each deletion. Data for WT and *stn1Δ* are reused from Figure 4C. See Supplemental Data 1 and 2 for the unfiltered data.

(D) Frequency of SDs, *i.e.* deletions ≤ 4 bp, for the indicated strains. Each dot represents an independent experiment.

(E) Frequency of IDs, *i.e.* 5-85 bp deletions, for the indicated strains.

Supplemental Table S1. List of strains. Related to STAR Methods.

Strain	Genotype	Figures	Reference
BY4742	*MATα his3Δ1 leu2Δ0 lys2Δ0 ura3Δ0*; 16 linear chromosomes	6, S1	Shao et al., 2018
SY13	*MATα his3Δ1 leu2Δ0 lys2Δ0 ura3Δ0*; 2 linear chromosomes	S1	Shao et al., 2018
SY14	*MATα his3Δ1 leu2Δ0 lys2Δ0 ura3Δ0*; single linear chromosome	1-3, 6, S1-S2	Shao et al., 2018
yZX168	*MATα his3Δ1 leu2Δ0 lys2Δ0 ura3Δ0*; single circular chromosome	1-6, S1-S6, S8	This work
yZX170	yZX168 *stn1::HIS3*	1-6, S2-S4, S8	This work
yZX226	yZX168 *cdc13::HIS3*	1-4, S2-S4	This work
yZX384	yZX168 *ten1:::HIS3*	1-4, S2-S4	This work
yZX207	yZX168 *dnl4::LEU2*	1-3, S2-S3	This work
yZX208	yZX168 *stn1::HIS3 dnl4::LEU2*	1, 3	This work
yZX285	yZX168 *tlc1::HIS3*	1, S3	This work
yZX283	yZX168 *pol4::HIS3*	1-2, S2, S5	This work
yZX274	yZX168 *yku80::LEU2*	1-2, 5, S2, S5	This work
yZX271	yZX168 *stn1::HIS3 yku80::LEU2*	1-4, S2, S5	This work
yZX292	yZX168 *pol1-D236N pol12-G325D*	1-2, 4-6, S2, S6	This work
yZX206	SY14 *dnl4::LEU2*	3	This work
yZX320	yZX168 *mre11-H125N*	5	This work
yZX321	yZX168 *stn1::HIS3 mre11-H125N*	5	This work
yZX408	yZX168 *sae2::LEU2*	5	This work
yZX415	yZX168 *stn1::HIS3 sae2::LEU2*	5	This work
yZX353	yZX168 *ChrII:460851-460853::LEU2[nt1-559] ChrII:480990-480988::LEU2[nt478-1089] **	5	This work
yZX368	yZX168 *stn1::HIS3 ChrII:460851-460853::LEU2[nt1-559] ChrII:480990-480988::LEU2[nt478-1089] **	5	This work
yZX455	yZX168 *pol1-D236N pol12-G325D ChrII:460851-460853::LEU2[nt1-559] ChrII:480990-480988::LEU2[nt478-1089] **	5	This work
yZX421	yZX168 *stn1::HIS3 pol1-D236N pol12-G325D*	6	This work
yZX308	SY14 *pol1-D236N pol12-G325D*	6	This work
yZX392	BY4742 *pol1-D236N pol12-G325D*	6	This work
yZX485	yZX168 *cdc13::HIS3 yku80::LEU2*	S2, S5	This work
yZX481	yZX168 *ten1::HIS3 yku80::LEU2*	S2, S5	This work
yZX483	yZX168 *pol4::HIS3 yku80::LEU2*	2, S2	This work
yZX490	yZX168 *pol4::HIS3 stn1::kanMX6*	S2, S5	This work
yZX486	yZX168 *rif1::LEU2*	S8	This work
yZX488	yZX168 *rev7::kanMX6*	S8	This work
JKM179	*MATα ura3-52 trp1::hisG' leu2-3,112 lys5 ade3::GAL10:HO hoΔ hmlΔ::ADE1 hmrΔ::ADE1 ade1-100MX6*	S7	Lee et al., 1998
yZX386	JKM179 *pol1-D236N pol12-G325D*	S7	This work
yKD2176	JKM179 *pol4::kanMX6 leu2-3::lacI-GFP-LEU2 4.4kb-MAT::lacOp-TRP1 NUP49::NUP49-mCherry-URA3*	S7	This work

Supplemental Table S2. List of plasmids. Related to START Methods.

Plasmid	Vector	Selection marker	Insert	Purpose	Reference for vector
pZX010	bRA66	*HPH1*	Guide RNA sequence: GTTAATGTGGCTGTGGTTTC	GAL1-driven Cas9 expression targeting 5' UTR of *URA3*	Anand et al. 2017
pZX013	bRA66	*HPH1*	Guide RNA sequence: AAGACAATCAATTACTACAA	GAL1-driven Cas9 expression targeting 5' UTR of *LYS2*	Anand et al. 2017
pZX026	pJH2970	*HIS3*	2 guide RNA sequences: AGCCATAATAGCATCCAGAT and TGAAACGCTGCCGTAAGCAG	Cas9 cut at subtelomeres of ChrX-R and ChrXVI-L for SY14 circularization	Anand et al. 2017
pRS426		*URA3*	None	Plasmid religation assay	Christianson et al. 1992

Supplemental Table S3. List of primers. Related to STAR Methods.

Primer name	Forward/Reverse	Sequence	Target locus	Use
oT1721	F	GGAAAGTTTCCACCAGACGCTAAGTGGTAGCCATAATAGCATCCACTTACGGCAGCGTTTCACTTTGTTTGGAGAACGGTTGTTAACTTG	Chimera between subtelomeres X-R and XVI-L.	Repair donor sequence for chromosome circularization
oT1722	R	CAAGTTAACAACCGTTCTCCAAACAAAGTGAAACGCTGCCGTAAGTGGATGCTATTATGGCTACCACTTAGCGTCTGGTGGAAACTTTCC	Chimera between subtelomeres X-R and XVI-L.	Repair donor sequence for chromosome circularization
oT1735	F	GCTTATTCTCAAATGGTGAC	XVI-L subtelomere (17.5 kb from end)	PCR to verify chromosome circularization and generate Southern blot probe
oT1736	R	ACTTCCCAATCATGAGGATC	X-R subtelomere (2.1 kb from end)	PCR to verify chromosome circularization and generate Southern blot probe
oZX513	F	CCACCTTGTCAGATTCGAAGG	Locus 0.95 kb away from Cas9 cut site at 5' UTR of LYS2	qPCR
oZX514	R	GCATTTACCGAAGTTTACTCCG	Locus 0.95 kb away from Cas9 cut site at 5' UTR of LYS2	qPCR
oT976	F	CTGGTATGTGTAAAGCCGGT	ACT1	qPCR
oT977	R	ACGTAGGAGTCTTTTTGACCCA	ACT1	qPCR
oZX108	F	CGCAACAGCCATCACAATCTC	RTS1	Control PCR for deletion mapping
oZX099	R	ATGTTCACCACATGAGCGTA	RTS1	Control PCR for deletion mapping
oZX526	F	AAGTATGCTCATCAATCGTTCGG	Flanking Cas9 cut site at 5' UTR of LYS2	Large deletion mapping by PCR; qPCR
oZX525	R	CAGACTTAGAAAGCTCTTCCCATA	Flanking Cas9 cut site at 5' UTR of LYS2	Large deletion mapping by PCR; qPCR
oZX641	F	CAAAGTGGTGATAGAGTTCA	Cas9 cut site at 5' UTR of LYS2 -652 bp	Large deletion mapping by PCR
oZX642	R	ACTGTAAATCAGCTGGCGTT	Cas9 cut site at 5' UTR of LYS2 -652 bp	Large deletion mapping by PCR
oZX664	F	TCAGATCGGATGTGCTTTA	Cas9 cut site at 5' UTR of LYS2 -3421 bp	Large deletion mapping by PCR
oZX665	R	GAGTGCTGTAAGGATTGT	Cas9 cut site at 5' UTR of LYS2 -3421 bp	Large deletion mapping by PCR
oZX666	F	TGCAGCTCTTTGGAACATG	Cas9 cut site at 5' UTR of LYS2 -4626 bp	Large deletion mapping by PCR
oZX667	R	ACTTGGCTCTCCATTGCTT	Cas9 cut site at 5' UTR of LYS2 -4626 bp	Large deletion mapping by PCR
oZX649	F	CCGTTCGACAGAACAAACC	Cas9 cut site at 5' UTR of LYS2 +1982 bp	Large deletion mapping by PCR
oZX650	R	GCACAGTTCTCCGACATT	Cas9 cut site at 5' UTR of LYS2 +1982 bp	Large deletion mapping by PCR
oZX639	F	TTCGACACTCCTTATTCAGGAC	Cas9 cut site at 5' UTR of LYS2 +2196 bp	Large deletion mapping by PCR
oZX640	R	AACGTCATTGCTCGGACATGT	Cas9 cut site at 5' UTR of LYS2 +2196 bp	Large deletion mapping by PCR
oZX651	F	CATGGGTAAAGAGAAGTCT	Cas9 cut site at 5' UTR of LYS2 +3235 bp	Large deletion mapping by PCR
oZX652	R	CTTCCACAAAGCAATATCGAT	Cas9 cut site at 5' UTR of LYS2 +3235 bp	Large deletion mapping by PCR
oZX425	F	AATGATACGGCGACCACCGAGATCTACACACACTCTTTCCCTACACGACGCTCTTCCGATCTTGCTCATCAATCGTTCGGAC	Flanking Cas9 cut site at 5' UTR of LYS2	P5 primer for Illumina sequencing.
oZX426-oZX435	R	CAAGCAGAAGACGGCATACGAGAT**[INDEX]**GTGACTGGAGTTCAGACGTGTGCTCTTCCGATCTTTCAGGCAGCAAGTGACCAT	Flanking Cas9 cut site at 5' UTR of LYS2	P7 primers for Illumina sequencing. INDEX indicates multiplexing barcode sequence.
oZX671	F	AATGATACGGCGACCACCGAGATCTACACACACTCTTTCCCTACACGACGCTCTTCCGATCTACCGAAGTTATCTGATGTAG	Flanking Cas9 cut site at 5' UTR of URA3	P5 primer for Illumina sequencing.
oZX672-oZX677	R	CAAGCAGAAGACGGCATACGAGAT**[INDEX]**GTGACTGGAGTTCAGACGTGTGCTCTTCCGATCTGCCCCGTAAAATACTGTTAC	Flanking Cas9 cut site at 5' UTR of URA3	P7 primers for Illumina sequencing. INDEX indicates multiplexing barcode sequence.
pr-1850	F	AATGATACGGCGACCACCGAGATCTACACACACTCTTTCCCTACACGACGCTCTTCCGATCTCTCACAGTTTGGCTCCGGTG	Flanking HO cut site at MAT locus	P5 primer for Illumina sequencing.
pr-1851-1855	R	CAAGCAGAAGACGGCATACGAGAT**[INDEX]**GTGACTGGAGTTCAGACGTGTGCTCTTCCGATCTTAAACAACCTCCGCCACGAC	Flanking HO cut site at MAT locus	P7 primers for Illumina sequencing. INDEX indicates multiplexing barcode sequence.
